# Noninvasive 3D mapping of tissue stress using reverberant shear wave

**DOI:** 10.1038/s41467-026-76509-0

**Published:** 2026-08-11

**Authors:** Shengyuan Ma, Suhao Qiu, Shun Yao, Wei Jin, Changwei Gu, Xiaona Li, Hexiang Sun, Yiwen Shen, Wenting Rui, Yuting Bao, Peijun Zhao, Qi Yue, Zhenwei Yao, Nidan Qiao, Qingfang Sun, Jun Liu, Fuhua Yan, Xi-Qiao Feng, Guang-Zhong Yang, Yuan Feng

**Affiliations:** 1 School of Biomedical Engineering, Shanghai Jiao Tong University, Shanghai, China; 2 Shanghai Key Laboratory of Flexible Medical Robotics, Tongren Hospital, Institute of Medical Robotics, Shanghai Jiao Tong University, Shanghai, China; 3 National Engineering Research Center of Advanced Magnetic Resonance Technologies for Diagnosis and Therapy (NERC-AMRT), Shanghai Jiao Tong University, Shanghai, China; 4 Department of Neurosurgery, Huashan Hospital, Shanghai Medical College, Fudan University, Shanghai, China; 5 Department of Neurology, Shanghai Sixth People’s Hospital affilated to Shanghai Jiaotong University School of Medicine, Shanghai, China; 6 Department of Neursurgery, RuiJin Hospital LuWan Branch, Shanghai Jiaotong University School of Medicine, Shanghai, China; 7 Department of radiology, Central Laboratory, RuiJin Hospital LuWan Branch, Shanghai Jiaotong University School of Medicine, Shanghai, China; 8 Department of Radiology, Huashan Hospital, Fudan University, Shanghai, China; 9 Department of Neurosurgery, Ruijin Hospital affiliated to Shanghai Jiao Tong University School of Medicine, Shanghai, China; 10 Department of Neurology and Institute of Neurology, Ruijin Hospital affiliated to Shansghai Jiao Tong University School of Medicine, Shanghai, China; 11 Department of Radiology, Ruijin Hospital affiliated to Shanghai Jiao Tong University School of Medicine, Shanghai, China; 12 Institute of Biomechanics and Medical Engineering, Department of Engineering Mechanics, Tsinghua University, Beijing, China

**Keywords:** Biomedical engineering, Magnetic resonance imaging, Acoustics, Computational science

## Abstract

Mechanical stress is a fundamental aspect of soft tissues that influences cellular behavior and tissue integrity, yet noninvasive imaging of stress in vivo remains a major challenge. Conventional magnetic resonance elastography (MRE) measures tissue stiffness but not stress. Employing a physics-informed model, we analyze reverberant shear wave fields recorded by MRE to capture the 3D mapping of tissue stress. By decomposing reverberant waves into anisotropic traveling wave components, we establish a direct relationship between wave speed, polarization, and local stress, without requiring wave direction knowledge. This method was validated with numerical simulations and phantom experiments. Applied to patients with meningiomas and pituitary adenomas, it generated high-resolution stress maps consistent with anatomy and physiology. Cortical stress measurements enabled intracranial pressure estimation, uncovering trends related to patient age and pathology. Our results demonstrate that noninvasive stress imaging provides a novel quantitative biomarker for mechanobiological research and suggests new possibilities for clinical applications.

## Abstract (web summary)

Mechanical stress shapes soft tissue health, but imaging it non-invasively in vivo has remained a challenge. Here, authors used physics-informed MRI wave analysis to map 3D tissue stress, enabling non-invasive intracranial pressure and tumor-induced stress estimates in brain tumor patients.

## Introduction

Mechanical stress is a fundamental regulator of tissue development, homeostasis, and disease progression^1–3^. It is ubiquitous across organ systems, including arteries, the heart, brain, and intestines, where it governs morphogenesis, structural stability, and physiological function^4^. Under normal conditions, tissues maintain a dynamic stress equilibrium that coordinates growth and function through mechanobiological feedback^5,6^. For instance, residual stress in arterial walls offsets hemodynamic loads to preserve vascular integrity^7^; stress produced by differential growth between cortical layers may drive brain folding^8^; and intestinal villi formation relies on stress generated by mismatched expansion of mucosal and muscular layers^9^. Disruptions to this mechanical microenvironment are increasingly recognized as hallmarks of disease. In tumors, abnormal proliferation and matrix remodeling elevate interstitial and solid stress, distorting local tissue mechanics^10^. These stress accumulations compress blood and lymphatic vessels, causing hypoxia and impairing immune infiltration^11,12^. Moreover, solid stress can activate oncogenic pathways such as β-catenin signaling^13^, promote tumor invasiveness, and even exert neurotoxic effects. Therapeutically, high solid stress poses a dual challenge: physically limiting the transport of drugs and immune cells^14^, and inducing immunosuppressive states through mechanotransduction pathways^11^. These findings underscore the importance of measuring in vivo stress, not only to understand tissue regulation but also to guide mechanical-targeted therapies.

Existing approaches fall into two categories: invasive and noninvasive. Invasive techniques, such as microdroplet deformation^12^, or biopsy-based mechanical testing^15–17^, can directly probe stress but are inherently destructive, unsuitable for delicate tissues like the brain, and incapable of longitudinal monitoring. However, the noninvasive quantification of stress in soft tissues, despite its profound clinical and research significance, remains a challenge. Noninvasive elastography methods detect acoustoelastic effects of stress-induced stiffness variations^18–20^, but typically infer stress indirectly through wave speed changes, facing key technical limitations. Direction-dependent methods^21^ require precise control of shear wave propagation^22,23^, a requirement that is often impractical in vivo. Other strategies introduce geometric or material constraints, such as assuming tumor spheroids^24,25^ or poroelastic parameters^26,27^, but these assumptions limit generalizability. Directional filtering approaches^28^ often discard valuable information, while data-driven models^29^ are hampered by limited training data and poor cross-context reliability. A fundamental limitation of prior work lies in the assumption of single-mode wave propagation, which contradicts the inherently reverberant nature of in vivo shear wave fields. As a result, accurate stress estimation in realistic environments has remained a longstanding challenge.

Here, we introduce a physics-informed framework that enables direct, noninvasive mapping of tissue stress from reverberant shear wave fields. By analytically decomposing complex wavefields into anisotropic traveling wave components, we resolve the relationship between wave speed, polarization, and local stress—without requiring knowledge of wave propagation direction. This operator-based model bridges elastodynamic behavior with hyperelastic stress, preserving the full mechanical information embedded in the wavefield. The method is validated in numerical simulations, gelatin phantoms, and clinical brain imaging. In particular, we demonstrate its ability to produce high-resolution stress maps in patients with meningiomas and pituitary adenomas, and to estimate intracranial pressure trends across age and pathology. Together, these results represent a step change in stress imaging: a generalizable, quantitative, and noninvasive technique for mapping mechanical stress in soft tissues in vivo.

## Results

### Noninvasive mapping of stress using reverberant shear waves

**Fig. 1:**
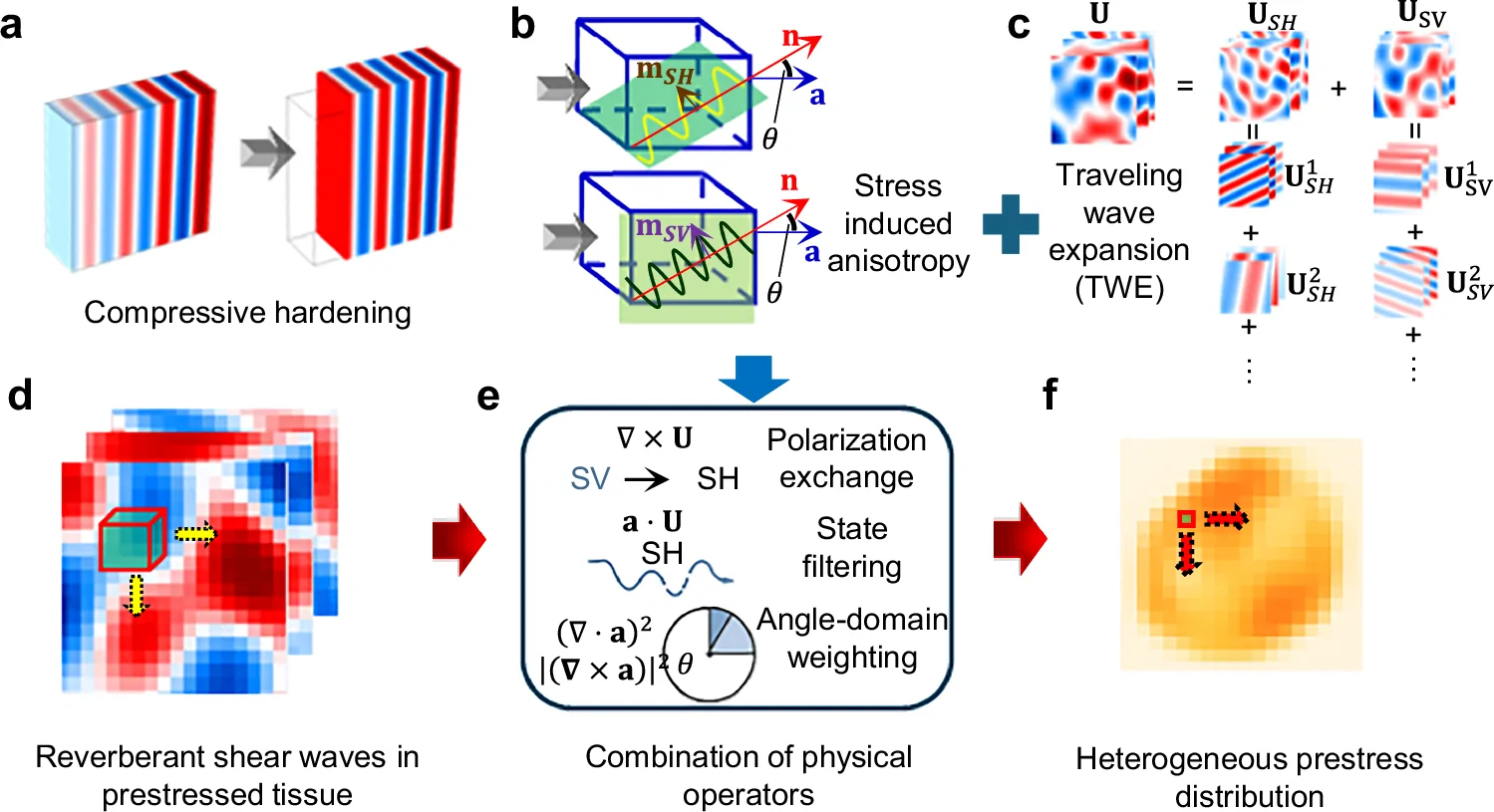
Overview of heterogeneous stress mapping using reverberant shear wave fields. **a** Schematic of wave-based mechanical imaging: tissue under compression becomes stiffer, and conventional shear wave elastography detects this via propagating waves along controlled excitation paths. **b** Under uniaxial stress in a Mooney–Rivlin hyperelastic material, shear waves exhibit direction- and polarization-dependent velocities. SV (shear vertical) and SH (shear horizontal) components show angular variation in speed depending on the propagation direction ${{{\boldsymbol{n}}}}$ relative to the stress axis ${{{\boldsymbol{a}}}}$. **c** In the reverberant regime, the shear wavefield is modeled as a superposition of SV and SH components traveling in multiple directions, forming the basis of the traveling wave expansion (TWE) model. **d** Complex reverberant shear wavefields encode spatially varying stress-induced anisotropy. **e** A set of physics‑based operators is derived from shear wave behavior under stress and the traveling wave expansion model, and their application within a 4×4×4 kernel (as in **d**) yields a local stress estimate. **f** A spatial map of heterogeneous stress is reconstructed by sliding the kernel throughout the reverberant shear wave field.

Stress alters the effective mechanical behavior of soft tissue by introducing anisotropy in wave propagation^19^. In particular, when a hyperelastic medium is subjected to uniaxial loading, small-amplitude shear waves traveling in different directions exhibit varying speeds depending on their polarization and alignment relative to the principal stress axis^30^. This acoustoelastic effect manifests as measurable shear wave anisotropy and serves as the physical basis for stress estimation^31^. A noninvasive imaging framework was developed to enable three-dimensional mapping of stress distributions in soft biological tissues by analyzing naturally reverberant shear wave fields acquired with magnetic resonance elastography (MRE) (Supplementary Movie 1). Unlike conventional elastography approaches that rely on controlled unidirectional wave excitation and modal isolation, this method explicitly leverages the reverberant nature of in vivo wave propagation (Fig. 1a). The composite wavefield is treated as a superposition of polarized traveling components that encode spatially varying stress-dependent mechanical properties (Fig. 1b), thereby eliminating the need for directional excitation or source characterization.

The brain is a soft, structurally complex tissue. While numerous models have been proposed to characterize its biomechanical behavior^32^, isotropy and incompressibility remain standard simplifying assumptions. Building on these assumptions, we propose a physics-informed traveling wave expansion (TWE) model. Derived from the hyperelastic Mooney–Rivlin constitutive model^33,34^, the TWE framework describes the reverberant wavefield as a linear superposition of shear-vertical (SV) and shear-horizontal (SH) wave components^35,36^ propagating along multiple directions (Fig. 1c). These components are governed by a stress-dependent constitutive model that links their velocities and polarizations to the underlying local stress state. To obtain this relationship from the measured displacement field (Fig. 1d), a structured set of spatial differential operators is applied. These operators are designed to enhance polarization contrast, suppress interfering components, and modulate angular sensitivity across directions (Fig. 1e). Operator-based filtering and analysis are performed locally within a sliding three-dimensional kernel (typically 4×4×4 voxels) (Fig. 1d, f), enabling voxel-wise computation of stretch ratio λ and uniaxial stress magnitude σ. Given either known or geometry-inferred principal stress directions, the processed wavefield is analytically inverted using acoustoelastic theory to directly yield local stress estimates. The inversion procedure simultaneously solves for local deformation and constitutive parameters, eliminating the need for prior knowledge of wave-propagation direction or material constants. This design enables robust stress reconstruction in heterogeneous and geometrically complex tissues. Importantly, the method does not rely on material-specific elastic constants or assumed propagation geometry, and it maintains stability in scattering or heterogeneous media. Full methodological details are provided in the Methods section. The resulting framework supports in vivo mapping of heterogeneous stress across diverse tissue types and loading conditions. In practice, anatomical features such as tumor boundaries or cortical surfaces may serve to infer stress directions, providing geometric constraints where internal orientations are unknown. This generalizability enables the wide applicability of the method in both experimental and clinical settings.

### Validation in pressurized hemispherical shell simulations

**Fig. 2:**
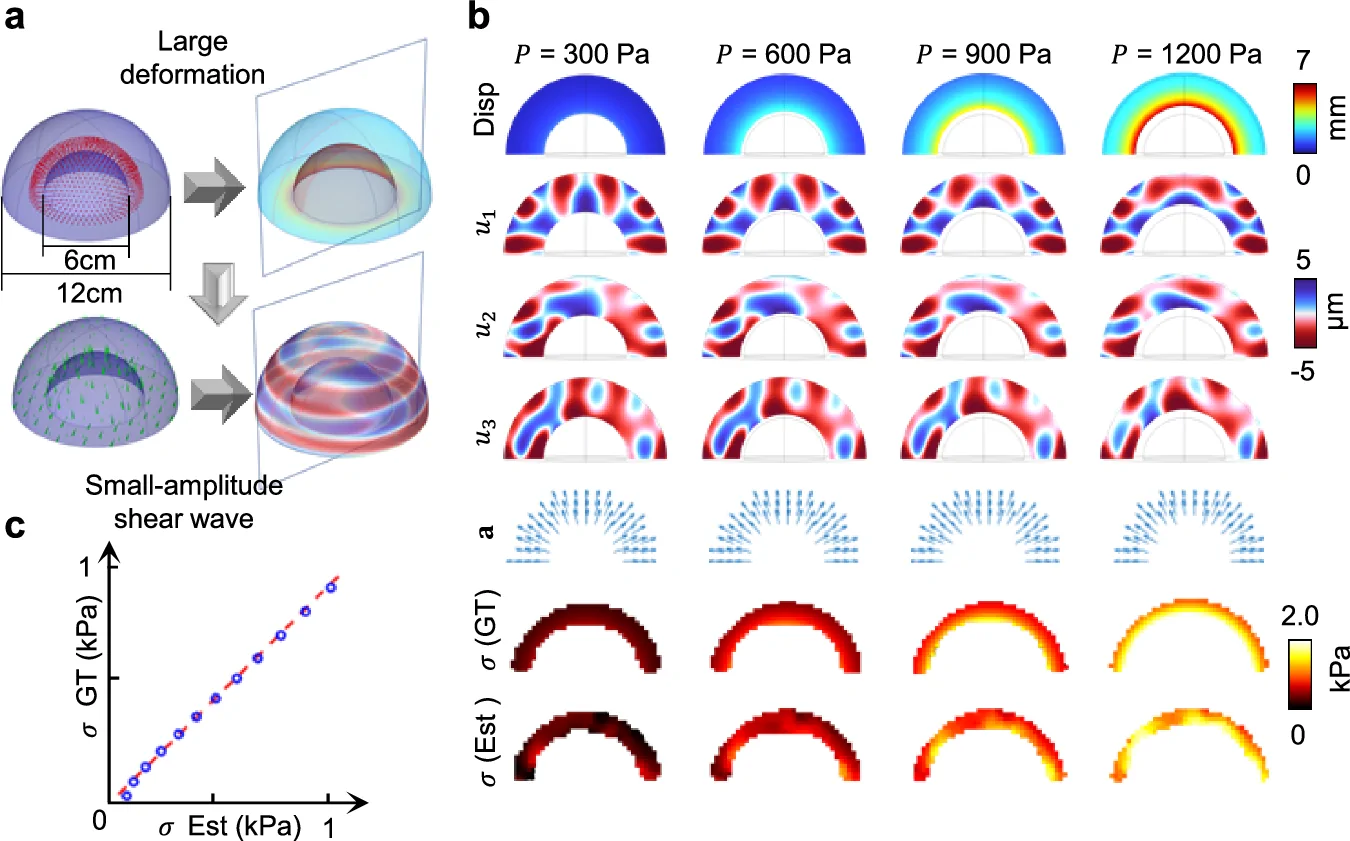
Stress inversion test in a hemispherical shell inflation simulation. **a** Schematic of the simulation setup: a spherical shell with an inner radius of 6 cm and an outer radius of 12 cm. In the first step, an internal pressure P is applied to the inner surface of the structure to induce large deformations; in the second step, a small-amplitude shear excitation is imposed on the deformed shell’s outer edge to generate shear wavefields. **b** Simulation and inversion results under varying pressures P. From top to bottom: displacement magnitude after large deformation, shear wavefields along three orthogonal directions, simulated stress direction field, and comparison between true and inverted stress values. **c** Comparison of mean stress values (true vs. estimated) over loading pressures from 100 Pa to 1200 Pa in increments of 100 Pa. The x and y coordinates of each blue circle represent the true and estimated values, respectively, and the red dashed line indicates the ideal *x = y* reference.

To assess the accuracy of the proposed reverberant shear wave–based stress inversion approach under complex geometrical and loading conditions, we evaluated a 3D hemispherical shell model subjected to large deformations. The model, featuring a 6 cm inner diameter and 12 cm outer diameter, was assigned hyperelastic properties with pressure applied to its inner surface under stepwise hydrostatic loading. Reverberant shear wavefields were then excited at 50 Hz and recorded on the deformed shell for inversion analysis (Fig. 2a).

As the applied pressure P increased from 100 Pa to 1200 Pa, the hemispherical shell exhibited significant radial displacement with a pronounced gradient from the interior to the edge (Fig. 2b). Correspondingly, the amplitudes of the reverberant shear waves across all three orthogonal directions exhibited systematic, distinguishable changes correlated with the stress level. The mean stress values estimated using the proposed method (Eqs. 3–4) exhibited agreement with the ground truth, closely aligning with the identity line (*x* = *y*) and yielding a coefficient of determination of *R*^2^ = 0.9978. The mean relative error was below 2%, indicating high accuracy of the reconstruction (Fig. 2c).

These results demonstrate the accuracy and robustness of the reverberant shear wave inversion framework for capturing spatially varying stress fields under nonlinear deformation, highlighting its potential for applications in biological tissues and other soft, heterogeneous media.

### Validation in heterogeneous embedded‑sphere inflation simulations

**Fig. 3:**
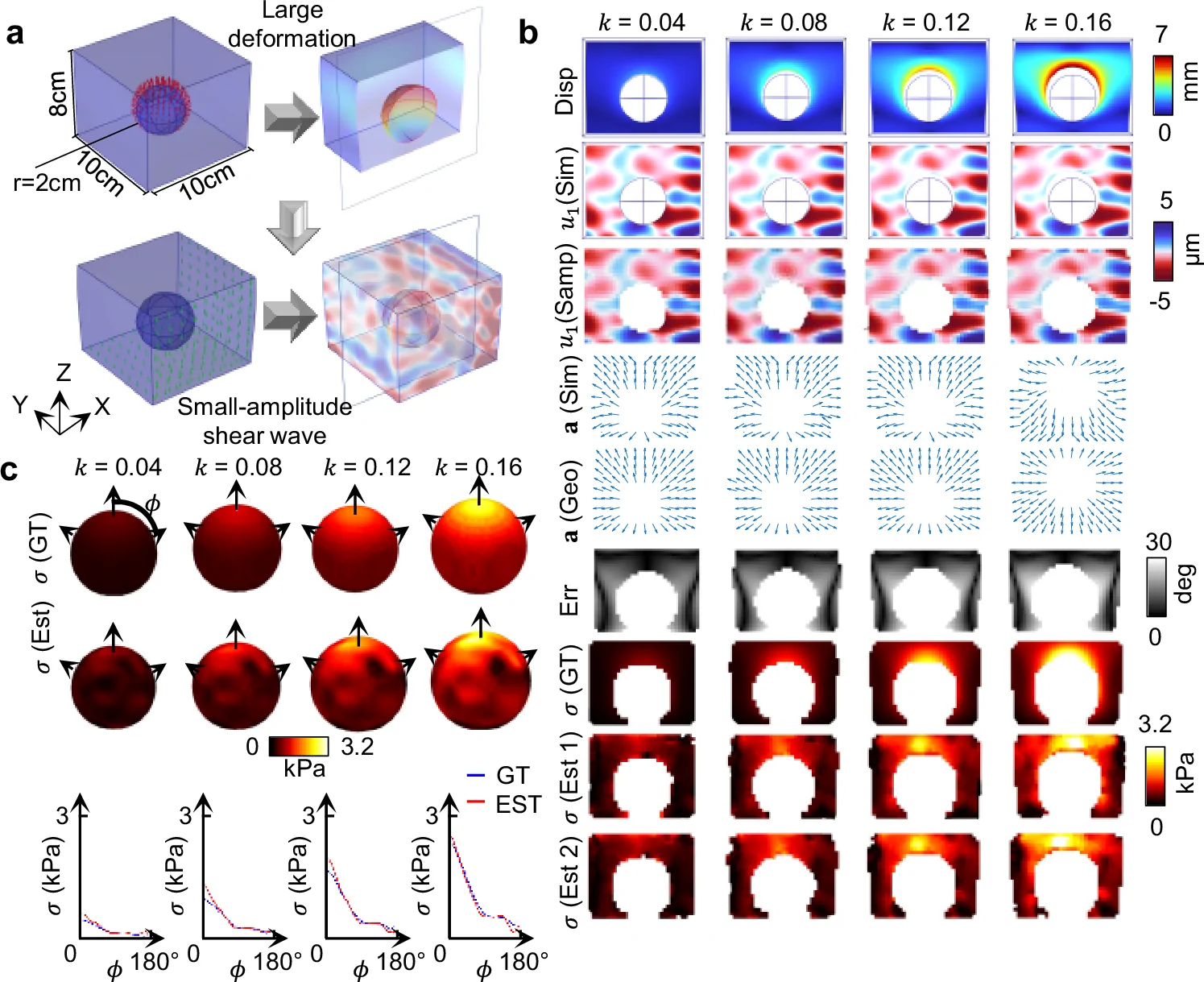
Reconstruction of heterogeneous stress in an embedded sphere simulation. **a** A spherical cavity is created within a 10 cm × 10 cm × 8 cm hyperelastic block. An inhomogeneous displacement field is first applied on the inner sphere surface to induce large deformation; subsequently, a 50 Hz small‑amplitude shear excitation is imposed on the deformed exterior surface to generate shear wavefields. **b** Comparison of simulation and inversion under varying deformation loads, shown top to bottom as: displacement magnitude after large deformation, FEM‑simulated shear wavefields and those sampled at 3 mm spacing for inversion, simulated stress direction field versus the stress direction field derived from surface normals, along with their angular error distribution, and comparison of true stress values with inverted estimates under two direction‑field definitions (Est1: from simulated field; Est2: from geometric normals). **c** Stress distribution on the spherical surface obtained via inversion using geometric normals, and mean true versus estimated stress values across different elevation angles ϕ.

To evaluate the inversion framework’s accuracy in the presence of heterogeneous stress distributions, we simulated an embedded spherical expansion scenario using COMSOL Multiphysics (v6.2). A 10 cm × 10 cm × 8 cm incompressible hyperelastic block containing a central spherical cavity (radius 2 cm) was defined (Fig. 3a) and characterized by a Mooney–Rivlin constitutive model. The simulation was carried out in two steps. First, we imposed a spatially heterogeneous radial displacement on the cavity wall: the bottommost node was held fixed, while all remaining nodes were uniformly expanded by a factor k—maintaining the cavity’s spherical geometry and yielding a final diameter of $2\left(1+k\right)$ cm. This loading generated a non‑uniform stress field throughout the surrounding medium. Second, once the block had reached its deformed configuration, low‑amplitude shear excitations at 50 Hz were applied to three adjacent faces to induce reverberant shear waves. The resulting displacement fields were recorded for subsequent inversion (Fig. 3b).

As the expansion ratio k increased, radial displacements on the upper hemisphere grew larger than those on the lower hemisphere, producing sharper deformation gradients in vertical cross-sections. The resulting shear wavefields exhibited distinct spatial modulation patterns that mirrored these deformation profiles. Stress inversion was then applied to the 3mm–sampled displacement data using two stress direction fields: one taken directly from the simulated ground-truth stress orientations, and the other inferred solely from surface geometry by assigning normals perpendicular to the cavity boundary—emulating the absence of true direction information in clinical tumor settings. Despite local angular deviations of up to 30° between the two fields, both inversion strategies reconstructed the spatial heterogeneity of stress around the cavity with high fidelity (Fig. 3b).

For a clearer visual comparison, the estimated stress distributions were projected onto the spherical surface (Fig. 3c), where—even when relying solely on geometry-derived directions (i.e., outward normals to the cavity boundary)—the reconstructed stress along the polar axis closely followed the ground truth, showing elevated stress in the upper hemisphere and reduced stress in the lower hemisphere. By averaging stress values along concentric spherical sections at varying elevation angles ϕ, the inversion curves quantitatively recapitulated the true polar and expansion-dependent trends across all tested k values (Fig. 3c). Crucially, assigning direction fields from surface geometry alone provides sufficient information for inversion, eliminating the need for prior knowledge of stress orientations. In gel-phantom sphere-inflation simulations, we performed two inversions: one using the true stress orientations and the other using surface normals. Both yielded equally accurate reconstructions of the heterogeneous stress field, with recovered polar-axis distributions, concentric-section averages, and expansion-dependent trends closely matching the ground truth.

These results demonstrate that surface-normal–based direction fields enable high-fidelity, non-invasive stress mapping, even under complex geometrical constraints where true stress orientations are inaccessible, as in typical in vivo settings.

### Validation in phantom experiments under spherical indentation

**Fig. 4:**
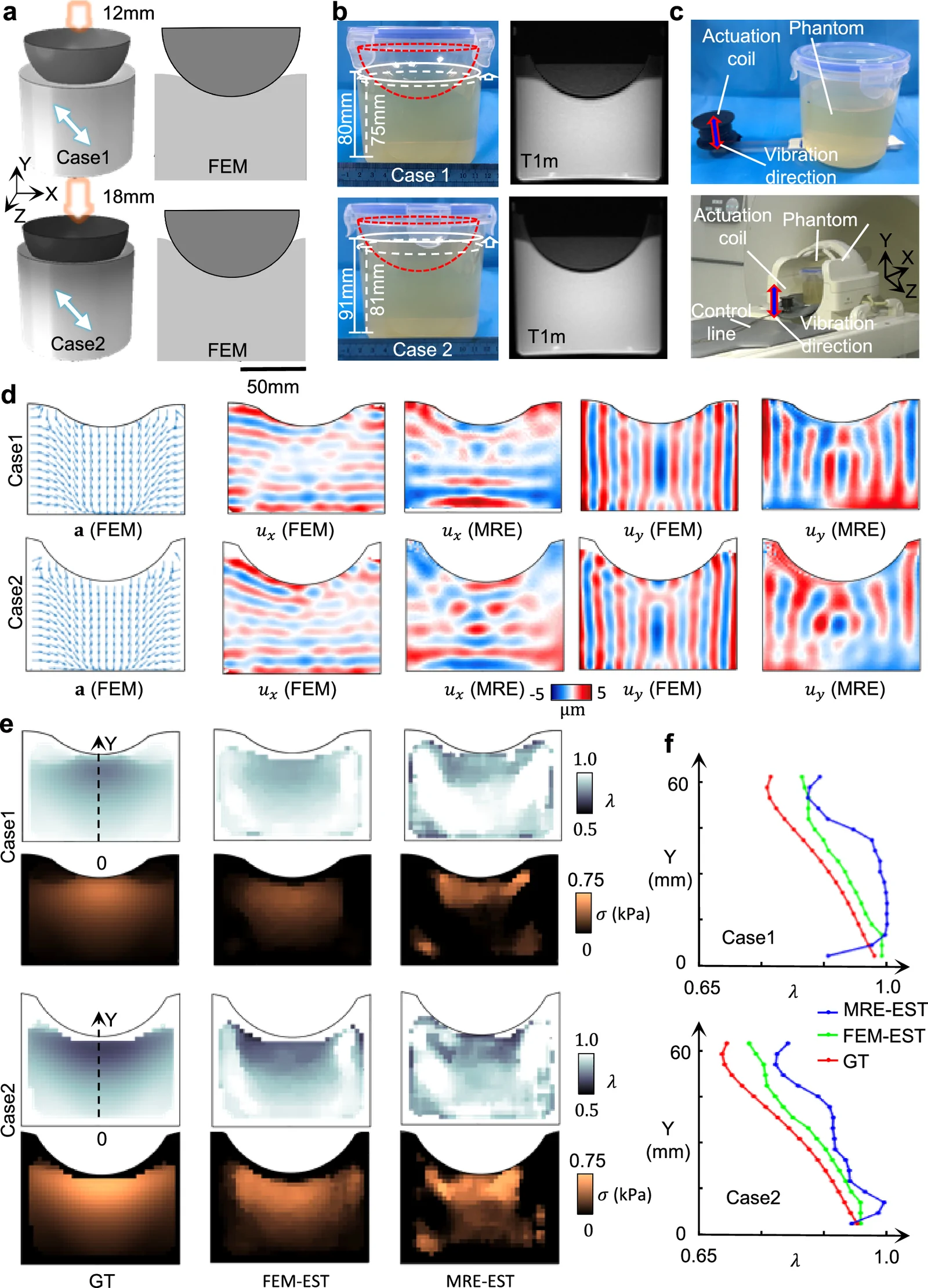
Simulated and experimental results of spherical indentation on a gelatin phantom. **a** Finite-element simulation schematics for two indentation depths (Case 1 and Case 2), showing the applied small-amplitude shear directions (yellow double arrows) and the deformed Y–Z cross-section. **b** Corresponding MRE experiments on transparent gelatin spherical shells indented to both depths (red dashed outlines) produced deformed T1-weighted MR images. **c** MRE experimental configuration, illustrating the phantom and actuator arrangement, MR acquisition method, and the direction of small-amplitude vibration. **d** Stress direction fields on the Y–Z cross-section from FEM, alongside the small-amplitude displacement fields in the x and y directions obtained both from FEM and MRE. **e** Ground-truth (GT) distributions of stretch ratio λ and stress σ for both indentation depths, compared with inversion results from FEM simulations and MRE wavefield. **f** Profiles of stretch ratio along the midline (Y) of the cross-section, showing ground truth, FEM-based estimates, and MRE-based estimates.

To rigorously evaluate the accuracy and robustness of our stress inversion framework under spatially heterogeneous loading, we performed two controlled experiments in which rigid hemispherical shells indented gelatin phantoms to two different depths, thereby imposing nonuniform stress fields with clear spatial gradients. Superimposed shear waves were then simulated via finite-element modeling (FEM) (Fig. 4a) and measured experimentally via MRE (Fig. 4b, c).

FEM simulations revealed that increasing indentation depth produced progressively steeper stretch-ratio λ gradients beneath the shell, with maximal deformation at the center decaying toward the periphery. Both simulated and MRE-measured wavefields exhibited consistent deformation geometries and propagation characteristics: distortions in the XY plane matched closely, while Z-direction motion remained negligible (Fig. 4d, e and Supplementary Fig. 8).

The stress inversion accuracy was validated using the FEM-derived prestrain distribution, computed under identical geometry and static boundary conditions with an incompressible material assumption, as a surrogate ground truth. Applying our wave-based inversion to both simulated and MRE-measured displacement fields, we recovered the heterogeneous stress landscape and precisely delineated the central high-stress region and peripheral gradients (Fig. 4e). Along the midline (x = 0), inversion of simulated data yielded stretch-ratio errors of just 5.3% and 5.5% at the two indentation depths, while inversion of MRE data exhibited errors below 11% (9.9% and 10.7%), despite minor boundary-condition mismatches (Fig. 4f). This close agreement confirms that our framework delivers accurate, spatially resolved stress measurements even under complex loading.

These results confirm that our inversion strategy maintains both robustness and spatial fidelity even under heterogeneous loading, underscoring its performance for noninvasive, in vivo stress mapping in complex biomechanical tissues.

### In vivo normal stress measurement of meningioma

**Fig. 5:**
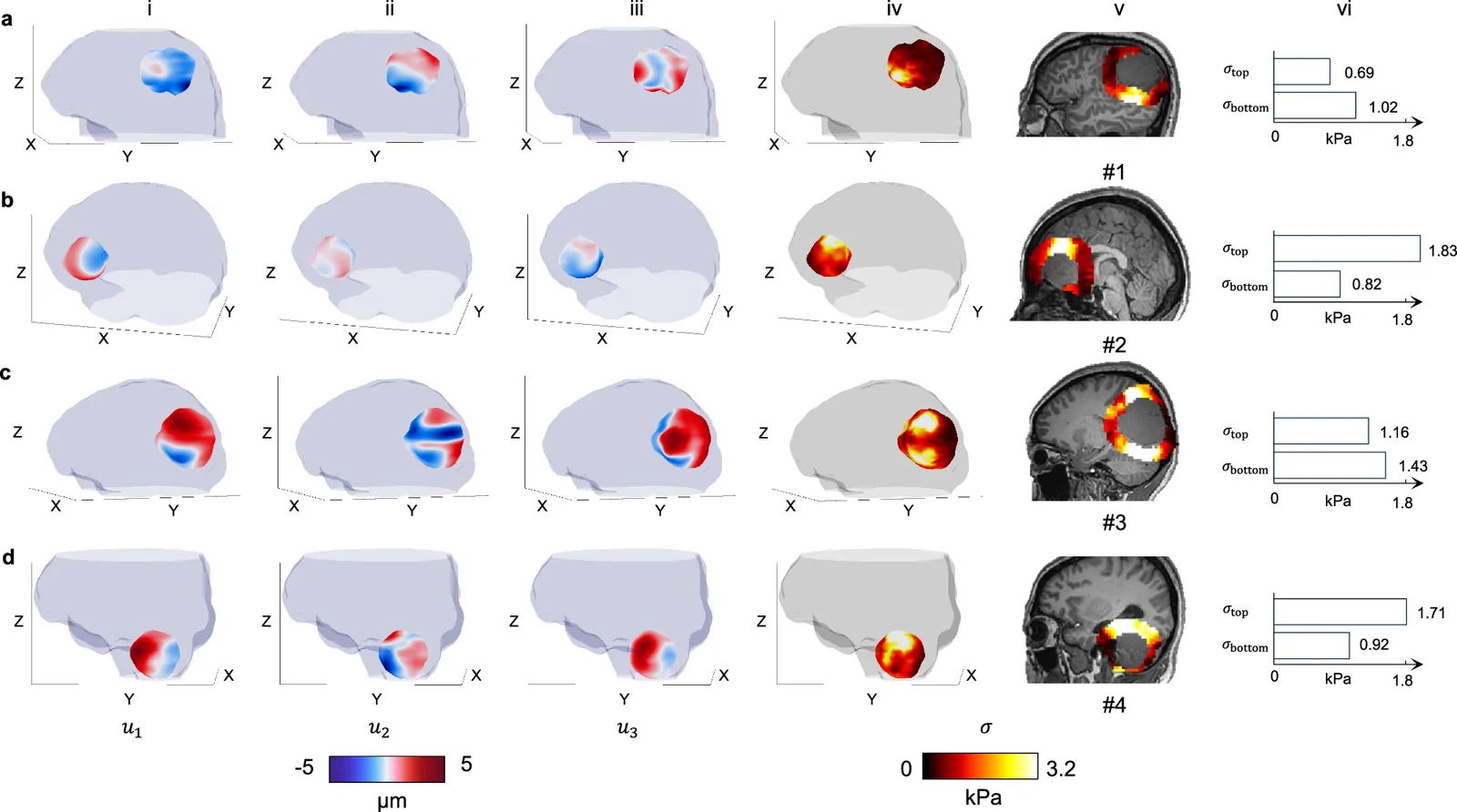
In vivo measurement of normal stress induced by meningiomas. Panels (**a**–**d**) correspond to four tumors situated in different anatomical regions (e.g., parietal, frontal, occipital, and temporal regions). In each panel, subpanels **i**–**iii** show the x, y and z components of the MRE shear wave displacement field mapped onto the 3D tumor–brain interface, subpanel **iv** depicts the resulting stress distribution on the same surface, subpanel **v** presents a sagittal T1-weighted slice overlaid with the 1.5 cm peritumoral stress map, and subpanel **vi** plots mean stress in the top ${\sigma }_{{top}}$ and bottom ${\sigma }_{{bottom}}$ hemispheres. Stress was sampled within a 1.5 cm radial margin around each tumor. ${\sigma }_{{top}}$ and ${\sigma }_{{bottom}}$ are the average stresses measured in the superior and inferior regions, respectively, defined by the horizontal plane passing through the tumor centroid.

To evaluate the feasibility and robustness of our wave‑based stress inversion framework under physiologically realistic conditions, we retrospectively analyzed MRE datasets from four patients with intracranial meningioma (Fig. 2). Shear wave displacement fields were acquired on a 3 T system, and tumor boundaries were segmented from co‑registered T1‑weighted images. Assuming that stress propagates along the local surface normal, we sampled wave information from a 1.5 cm peritumoral shell (5 pixels) to constrain the inversion. This produced high-resolution, 3D maps of stress at the tumor–brain interface, revealing spatially heterogeneous mechanical loads in vivo (Fig. 5 and Supplementary Fig. 8). The reconstructed stress fields exhibited strong anatomical correspondence with calvarial constraints, providing biomechanical plausibility for the inferred spatial patterns. For quantitative assessment, we define the “top” and “bottom” hemispheres by the horizontal plane passing through the tumor centroid, where ${\sigma }_{{top}}$ and ${\sigma }_{{bottom}}$ are the average stresses measured in the superior and inferior regions relative to this plane, respectively.

Situated in various anatomical regions of the cranial vault (e.g., parietal, frontal, occipital, and temporal regions), the tumor volumes ranged from 32.4 to 116.5 cm³ (diameters 3.96–6.06 cm), with reconstructed stress magnitudes lying predominantly in the kPa regime^37^. These values align with known brain tissue viscoelasticity and underscore the method’s ability to capture physiologically relevant compressive loads across distinct anatomical levels.

The parietal lobe lesion (#1, 58.3 cm³, Ø 4.81 cm, Fig. 5a) exhibited a modest inferior bias, with apex stress of 0.69 kPa rising to 1.02 kPa at the base. This pattern reflects expansion against the falx cerebri and downward compression of the adjacent cortex, in line with mechanical constraints imposed by the interhemispheric fissure.

In the frontal lobe tumor (#2, 32.4 cm³, Ø 3.96 cm, Fig. 5b), we observed pronounced superior polarization, with stress peaking at 1.83 kPa at the apex and falling to 0.82 kPa inferiorly. Upward growth beneath the convex parietal vault, where firm dural attachments and calvarial curvature limit downward bulging, likely concentrates compressive load at the upper boundary.

The large occipital lesion (#3, 116.5 cm³, Ø 6.06 cm, Fig. 5c) displayed nearly balanced stresses (1.16 kPa superior vs. 1.43 kPa inferior), indicative of symmetric mechanical resistance by the tentorium and surrounding dura. Such an equilibrium is expected for posterior‑midline masses constrained equally above and below by rigid anatomical structures.

The temporal lobe tumor (#4, 39.6 cm³, Ø 4.23 cm, Fig. 5d) again favored the superior pole, with apex stress of 1.71 kPa compared to 0.92 kPa at the base. Tight dural attachments along the middle cranial fossa roof and the concave bone surface limit inferior displacement, focusing compressive load on the upper margin.

All cases yielded high-resolution, three-dimensional maps of peritumoral stress that reflected each tumor’s morphology and local anatomical constraints: convexity and temporal lesions concentrated loads at their superior poles beneath the calvarial vault; frontal masses exhibited a modest inferior bias due to falcine confinement; and posterior midline tumors displayed near-symmetric loading across the tentorial plane. Peak stresses at the meningioma–brain interface exceeded 3 kPa, well above normal intracranial pressure (0.7 ~ 2.0 kPa)^38^ and the negative interstitial fluid pressures (− 1.5 ~ − 0.4 kPa)^39^ that govern cerebral fluid balance, and approached the intrinsic elastic stiffness of healthy brain tissue (1 ~ 5 kPa). These site-specific stress signatures underscore our framework’s sensitivity to true physiological variations in skull geometry and dural tethering, reveal the substantial solid forces meningiomas exert on surrounding parenchyma, and, by quantifying these heterogeneous in vivo force landscapes, open new avenues for biomechanical phenotyping, peritumoral edema insight, and individualized preoperative risk stratification and therapeutic planning.

### Quantitative mapping of optic chiasm normal stress in pituitary adenomas

Pituitary adenomas frequently cause visual impairment through optic chiasm compression, which disrupts axonal transport and impairs retinal ganglion cell function^40^. Although surgical decompression is the clinical standard, up to 40% of patients suffer irreversible visual loss^41^, necessitating better tools for prognostic assessment. Current methods like optical coherence tomography and diffusion imaging often lack sensitivity to early functional changes^42^. Given that mechanical stress is known to impair microcirculation and axonal health, and may precede permanent structural damage^43^, its quantitative in vivo assessment along the visual pathways represents a critical but currently unmet clinical need.

**Fig. 6:**
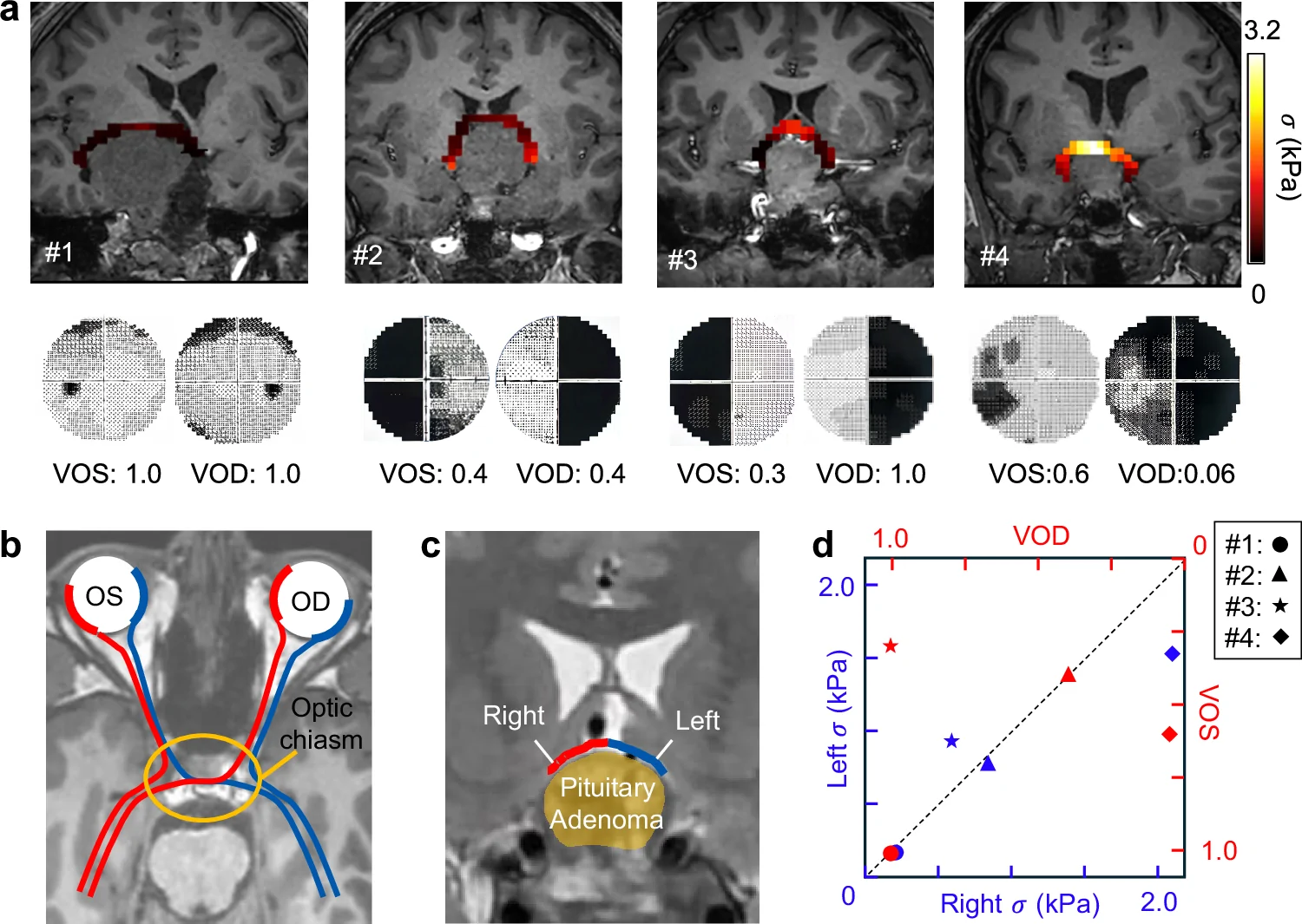
Normal stress measurement results of optic chiasm compression by pituitary adenoma. **a** T1‑weighted structural images overlaid with reconstructed stress distributions in the optic chiasm of four patients. Each panel is annotated with the patient’s corrected visual acuities (OS/OD) and temporal field outcomes. Note that the MR display convention inverts actual laterality (displayed “left” → patient’s right, and vice versa); all lateral labels and analyses below have been corrected to reflect true anatomical sides. **b** Schematic of the ipsilateral relationship between chiasm stress and temporal visual field: increased stress on the anatomical left chiasm produces temporal field deficits and acuity loss in the left eye (OS), while right‑side compression affects the right eye (OD). **c** T2‑weighted image from patient 3 with the segmented tumor mask, and the two regions of interest used for stress quantification in the true left (blue) and true right (red) chiasm. **d** Scatter plot of mean stress in the true left and right chiasm regions (blue markers, lower and left axes) versus corresponding ipsilateral visual acuity (red markers, upper and right axes). A strong inverse correlation is evident between local stress magnitude and ipsilateral visual function.

Here, we present the first high-resolution 3D mapping of normal stress within the optic chiasm in four patients with pituitary adenoma, acquired by combining MRE with wave-based inversion. We collected both MRE and T1-weighted data on a 3 T MRI system (United Imaging, Shanghai, China), using the same acquisition scheme as in our meningioma studies. Consistent with established neuroanatomy, left-sided stress elevation in the optic chiasm corresponded with temporal visual field deficits in the left eye, while right-sided stress increases aligned with right-eye temporal field impairment. This laterality-specific pattern was observed in all four patients (Fig. 6 and Supplementary Fig. 10).

Quantitative analysis revealed a robust inverse relationship between regional stress and visual function (Fig. 6c, d). Patient 1 exhibited low average stress ( ~ 0.3 kPa) and maintained normal binocular acuity (1.0) with intact fields. Patient 2 showed bilaterally elevated stress ( ~ 0.9 kPa), accompanied by bilateral temporal field defects and reduced acuity ( ~ 0.4). In Patient 3, asymmetric stress (1.0 kPa left vs. 0.6 kPa right) corresponded precisely to severe left‑eye acuity loss (0.3) with preserved right‑eye vision, while both eyes demonstrated temporal field defects. Patient 4 presented the highest stress levels (2.0 kPa left, 1.5 kPa right), matching dramatic left‑eye vision loss (0.06) and extensive field deficits, whereas the right eye retained moderate acuity (0.6) with localized temporal scotomas.

In vivo heterogeneous stress mapping revealed two key findings in patients with pituitary adenomas. First, regional stress magnitude correlated with ipsilateral temporal field defects and acuity loss; second, intra-individual stress asymmetries mirrored the lateralization of visual dysfunction, underscoring the critical role of the local mechanical microenvironment. These results provide the first in vivo biomechanical validation that compressive stress contributes to optic neuropathy, surpassing traditional morphological imaging in predictive power. Moreover, the observed correlation between stress and visual impairment supports the pathophysiological hypothesis that impaired axonal transport underlies vision loss^40,44,45^. Notably, our method detected subclinical compression as low as 0.3 kPa (e.g., patient #1 in Fig. 6a, who exhibited no visual deficits), suggesting potential utility as an early mechanical warning signal. Together, these findings lay the groundwork for MRE-based preoperative assessment of chiasmal compression, with potential to inform surgical timing, guide individualized treatment strategies, and predict postoperative visual recovery from a biomechanical perspective.

### Non-invasive estimation of intracranial pressure from cerebral surface stress

The accurate measurement of intracranial pressure (ICP) is critical for neurocritical care and the diagnosis/treatment of brain diseases. Traditional methods rely on invasive procedures, i.e., ventricular catheter insertion or lumbar puncture, with risks of infection and operational complexity^46^. Since ICP applied to the cortical surface can be treated as stress induced by uniaxial compression from cerebrospinal fluid (CSF), we propose using the method developed in this study for in vivo ICP measurement. The cortex was modeled as an elastic solid immersed in pressurized CSF, with stress values derived from a 9-mm inward shell along surface normals serving as an indirect metric of ICP.

Four participant groups were enrolled: (1) healthy young adults (*n* = 18, age 23 ~ 28, mean 24), (2) healthy elderly adults (*n* = 20, age 60 ~ 80, mean 70), (3) mild cognitive impairment (MCI) patients (*n* = 15, age 62 ~ 79, mean 69), and (4) elderly patients with normal pressure hydrocephalus (NPH) (*n* = 15, age 61 ~ 86, mean 75). MRE was performed using a 3 T MRI scanner (United Imaging, Shanghai, China) with the same parameters as in the meningioma studies.

**Fig. 7:**
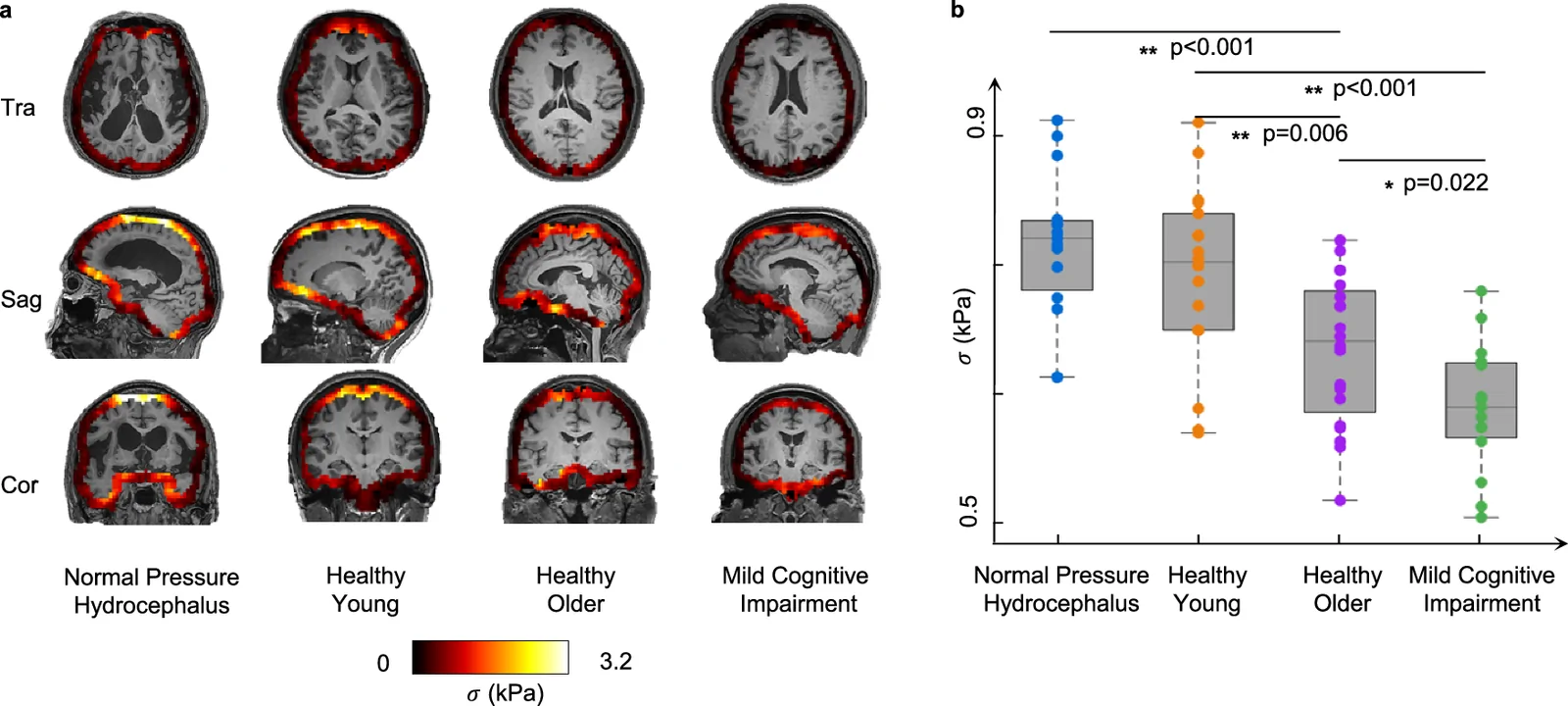
Intracranial pressure measurements across different populations. **a** Typical cortical surface pressure distribution patterns are shown in transverse (Tra), sagittal (Sag) and coronal (Cor) views for a patient with normal pressure hydrocephalus (NPH), a healthy young group, a healthy elderly group and an elderly mild cognitive impairment (MCI) group. **b** Box plots summarize the cortical surface pressure distributions for NPH patients, healthy young adults, healthy elderly adults, and elderly MCI patients. The box range covers from 25% to 75%, with statistical significance marked by asterisks (*: *p* < 0.05, **: *p* < 0.01).

Results (Fig. 7 and Supplementary Fig. 11) demonstrated a clear, age-dependent decline in estimated intracranial pressure (ICP): healthy young adults exhibited the highest values (737.8 ± 97 Pa), followed by healthy elderly adults (652.8 ± 82 Pa), and patients with mild cognitive impairment (590.2 ± 72 Pa). All intergroup differences reached statistical significance (*p* < 0.05), mirroring reported age-related ICP decreases ( ~ 0.69 mmHg per decade)^47^ and implicating cortical atrophy–mediated reductions in CSF pressure and altered brain hydrodynamics. Notably, the additional 62.6 Pa drop in the MCI cohort versus elderly controls underscores this metric’s sensitivity to early cognitive decline, potentially reflecting impaired CSF-ISF exchange and glymphatic dysfunction^48,49^.

In contrast, patients with normal-pressure hydrocephalus (NPH; mean age 75 years) showed elevated ICP (780.0 ± 76 Pa), significantly above elderly controls (*p* < 0.001) yet statistically indistinguishable from young adults (*p* > 0.05). This increase likely arises from compensatory CSF accumulation in ventricles and subarachnoid spaces, partially offsetting age-related atrophy. All NPH values remained below the clinical “normal-pressure” threshold ( < 18 cm H_2_O, ~ 1765 Pa)^50^, consistent with prior MRE findings of increased brain stiffness in NPH^51,52^.

Within-group coefficients of variation were uniformly low (13.2% young, 12.5% elderly, 12.2% MCI, 9.7% NPH), all under 15%, indicating excellent reproducibility of this stress-based ICP assessment. Collectively, these data establish cortical stress mapping as a robust, noninvasive correlate of ICP, capturing physiologically meaningful variations across aging, neurodegeneration, and hydrocephalus, and highlight its translational potential for early disease detection and multimodal evaluation of glymphatic clearance.

## Discussion

We introduce a noninvasive method to measure in vivo stress in soft tissues using reverberant shear waves. A key theoretical advance is the use of a traveling wave expansion model constrained by the Mooney–Rivlin hyperelastic law, from which we derive an analytical link between the local stress tensor and the reverberant wavefield. This clear formulation enables a quantitative inversion algorithm that exploits the acoustoelastic effect (stress-induced changes in wave speed) to estimate spatially heterogeneous stress without prior wave direction control. We validated the framework with comprehensive numerical simulations and phantom experiments. The simulations included scenarios with spatially varying stress fields, such as surface indentation of a hemispherical shell and inflation of a hollow sphere (Figs. 2, 3, Supplementary Notes 3–4, Supplementary Figs. S1, S2). In each scenario, the inversion algorithm accurately reconstructed the heterogeneous stress distribution. Phantom and human experiments (Figs. 4–7) further demonstrate the reliability and robustness in mapping stress.

In comparison to previous stress estimation methods, the proposed method overcomes the physical bottlenecks of conventional acoustoelasticity and model-based elastography. Acoustoelasticity typically requires prescribed wave propagation modes or known wave directions^18–20^, which are challenging for the brain due to cranial reflections and tissue heterogeneity. While recent orthogonal-wave methods^22^ elegantly demonstrate stress recovery in soft materials, the required precise wavefield steering is also challenging for deep and complex tissues. Furthermore, existing reverberant elastography frameworks^53–55^ generally assume a statistically isotropic, diffuse wavefield, relying on the expectation over all propagation directions to estimate properties. However, the presence of significant tissue damping and limited excitation sources often results in a “finite” or incomplete reverberance that may not satisfy the statistical assumptions. The proposed method differs fundamentally by embracing these uncontrolled, “finite” reverberant fields. Instead of imposing physical wave control or relying on perfect wavefield isotropy, we utilize an operator-based inversion that mathematically untangles the complex wavefield^56^. Much like a series of calibrated rotations to restore a Rubik’s Cube, our tailored sequence of differential operators acts on the diverse wavelets in the reverberant field, isolating stress-induced signatures. Unlike prior methods that attempt physical wavefield preconditioning, the proposed method focuses on mathematically deconditioning the naturally complex wavefield to extract acoustoelastic signatures. This enables us to establish an analytical link between non-cooperative wavefields and 3D stress distributions, without the need for perfect reverberance or material constant calibration. By avoiding the directional filtering required by earlier methods^28^, our approach preserves the full anisotropy of the wavefield, enabling robust, high-resolution stress mapping across the entire field of view.

Beyond its theoretical advances, our method bridges invasive and noninvasive measurement paradigms. Traditional direct stress techniques, such as tumor excision, indentation^15–17^, or microdroplet injection^57^, provide accurate values but are destructive and unsuitable for in vivo longitudinal studies. In contrast, many noninvasive methods, such as stiffness-based elastography or indirect wave anisotropy analysis, typically rely on simplified assumptions about tissue geometry or material properties^24,25^ and generally cannot directly resolve true stress^26,27^. Our approach directly relates the measured wavefield to the underlying hyperelastic stress tensor with minimal a priori assumptions. We perform localized fitting within small 3D voxel patches (e.g., 4 × 4 × 4 voxels), enabling high spatial resolution and flexibility in complex anatomy. Crucially, this allows us to resolve heterogeneous stress patterns from a single volumetric acquisition, for example, peritumoral tensile rings, compressive zones at anatomical constrictions, or stress gradients at tissue interfaces. In tumor contexts, where local mechanical heterogeneity drives pathophysiology, this full-field, high-resolution, single-acquisition stress mapping opens new avenues for clinical assessment and mechanobiological investigation.

Direct in vivo stress mapping also has broad implications for understanding mechanobiology. Mechanical stress is a fundamental regulator of tissue form and function, but its distribution in living tissues has been largely invisible^22^. For example, differential growth across cortical layers generates in-plane stresses that drive the folding of the brain’s surface (gyrification)^58^. Our method could test this by visualizing stress patterns in developing brains or organoid models. Similarly, growth mismatches produce buckling in other systems (e.g., intestinal villi formation), and quantifying those stresses could inform models of morphogenesis^9^. In cancer, solid stress within tumors is known to compress blood vessels and alter cell behavior^10^, so noninvasive stress maps could serve as novel biomarkers for tumor mechanics. By combining stress imaging with molecular or functional imaging, one could study how mechanical forces influence gene expression and signaling pathways during development and disease^11^. Our approach thus paves the way toward in vivo “stress histology,” linking mechanical forces to biological outcomes across fields from neurodevelopment to oncology.

In clinical contexts, stress imaging adds a new dimension to diagnostics by capturing biomechanical changes that may precede or complement anatomic alterations. We tested the translational potential in three representative scenarios: stress mapping near meningiomas, optic chiasm compression by pituitary adenomas, and cortical surface stress as a proxy for intracranial pressure (ICP). In patients with meningiomas, we found peritumoral stresses of 1 ~ 3.5 kPa, values comparable to the intrinsic stiffness of brain tissue^51,52^ and far exceeding normal interstitial or intracranial pressures^38,39^. Such elevated stresses could disrupt local microcirculation, impair axonal transport, and remodel the extracellular matrix^59^. Importantly, the stress distribution followed tumor margins and nearby anatomical boundaries, providing insights into tissue–tumor interactions beyond conventional imaging features.

In patients with pituitary adenomas, even sub-kPa stress along the optic chiasm correlated with characteristic bitemporal visual field deficits^40,43–45^. We detected stresses as low as ~ 0.3 kPa in patients with only mild symptoms, suggesting that stress mapping could aid early diagnosis and surgical risk assessment. In studies of healthy and cognitively impaired individuals, cortical stress patterns reflected known ICP dynamics: we observed an age-dependent decline in mean cortical stress^60^ and abnormal regional loading in mild cognitive impairment^61^, potentially linked to glymphatic dysfunction. In patients with NPH, cortical stress was markedly elevated, consistent with increased ICP^50^ and with independent MRE-based measures of brain stiffness^51,52^. Together, these findings validate in vivo stress as a meaningful mechanical biomarker, capable of capturing both local and global changes across neurological conditions. Integrating stress mapping with existing imaging techniques could enhance early detection, enable mechanobiological phenotyping, and guide personalized treatment in neuro-oncology, neurodegeneration, and cerebrospinal fluid disorders.

The full stress state in tissue is described by a second-order tensor comprising six independent components, making the recovery of all components from a single shear-wave measurement impractical. Under the incompressibility assumption, shear-wave propagation is insensitive to hydrostatic pressure, leaving the full three-dimensional tensor theoretically underdetermined. To render the problem tractable, we adopted an isotropic hyperelastic model and focused on the dominant uniaxial stress component in a 3D formulation. Under a general triaxial state, the inverse problem is severely underdetermined, and all three principal directions would need to be known at every voxel, information unavailable from a standard MRE acquisition. The uniaxial assumption reduces the system to a single stretch ratio, yields a determined inverse problem, and requires only one anatomically inferable direction. This simplification aligns with physiological observations where loading is predominantly normal to tissue boundaries^16,62^. Although these assumptions may not fully capture the complete tensor complexity in highly multiaxial environments, numerical simulations (Supplementary Note 5) demonstrate that the framework robustly recovers the primary deviatoric stress with less than 5% error under moderate lateral loading (lateral ratio up to 1/3).

The achievable spatial resolution of the proposed stress-mapping framework is primarily determined by the spatial resolution of the underlying MRE acquisition; therefore, extremely localized stress concentrations at sub-voxel scales cannot be fully resolved under current MRE resolution limits. Furthermore, our quasi-local homogeneity assumption is predicated on the local shear wavelength; while a standard simplification in elastography, its capability to resolve clinically relevant gradients is supported by our numerical simulations (Figs. 3, 4) and in vivo results (Fig. 5). Supplementary Note 9 demonstrates that the framework accurately recovers stress in media with spatially graded material properties (modulus gradients up to 10 kPa/m), with maximum estimation errors remaining below 15%. Although the sliding window approach optimizes the balance between local sensitivity and inversion stability, the current resolution may still pose challenges for mapping stress in fine-scale anatomical structures.

Several potential sources of modeling bias are examined in the Supplementary Information. Supplementary Note 6 compares wave‑speed variations induced by stress with those arising from tissue mechanical anisotropy, confirming that acoustoelastic effects dominate directional wave‑speed changes. Across the physiologically relevant damping ratio range at 50 Hz (0.1 ~ 0.3), evaluations in Supplementary Note 7 show that estimation errors increase monotonically but remain bounded (up to 23%), and the spatial pattern is well preserved. The proposed method provides bulk quantitative estimates of stress. While the overall magnitude and trends are consistent with expected values, the estimates may be refined with more complex models or additional constraints. However, the general magnitude is expected to remain stable. Supplementary Note 8 further demonstrates, through noise‑propagation simulations, that the proposed framework remains stable under realistic noise levels.

Future work should relax these simplifying assumptions to account for compressible, viscoelastic, or mechanically anisotropic tissues, and validate the method in large multi-center clinical studies, including dedicated viscoelastic phantom experiments, to test its robustness across pathologies, MRI scanners, and imaging protocols.

In summary, we introduce a new noninvasive technique to map in vivo mechanical stress using reverberant shear waves. The method offers clear advantages in challenging clinical scenarios: it enables quantitative mapping of tumor-induced stress, optic pathway compression, and intracranial pressure. These capabilities highlight the broad clinical relevance of stress imaging and its potential to improve diagnostics in neuro-oncology and beyond. Looking ahead, combining stress maps with complementary MRI biomarkers such as perfusion imaging or diffusion-based glymphatic indices^63,64^ may provide more comprehensive mechanobiological profiles of brain disorders. Extending our framework to incorporate anisotropic or viscoelastic constitutive models will expand its utility to structurally complex tissues (e.g., cardiac muscle and white matter) and to applications in cardiovascular medicine and bioengineering. Ultimately, the ability to noninvasively quantify stress in vivo opens new research frontiers and provides a unique mechanical perspective on biological function and disease.

## Methods

### TWE model-guided analytical inversion for stress

The soft tissue is modeled as an incompressible hyperelastic medium following the Mooney-Rivlin formulation^30^, where ${C}_{10}$ and ${C}_{01}$ characterize material behavior under large deformation. Although the stress state in biological tissue is generally multi-axial, shear wave propagation in an incompressible medium is insensitive to the hydrostatic component and responds to the deviatoric part of the stress tensor (Supplementary Note 5). The proposed inversion recovers the deviatoric stress difference along the dominant loading direction under a uniaxial-dominant assumption, which provides both analytical tractability and a well-posed inverse problem.

When subject to uniaxial stress, shear waves demonstrate directional anisotropy, defined by the angle θ between the propagation direction ${{{\bf{n}}}}$ and the principal stress axis ${{{\bf{a}}}}$. The resulting wavefield consists of two polarized modes: SV wave (Shear Vertical) mode and SH wave (Shear Horizontal) mode. With ρ denoting the material density, wave velocities v and polarization vectors ${{{\bf{m}}}}$ satisfy^31^:

1 $$\left\{\begin{array}{c}\rho {v}_{{SH}}^{2}={\mu }_{A}{\cos }^{2}\theta+{\mu }_{B}{\sin }^{2}\theta \\ \rho {v}_{{SV}}^{2}={\mu }_{A}{\cos }^{2}\theta+{\mu }_{C}{\sin }^{2}\theta \\ {{{{\bf{m}}}}}_{{SH}}={{{\bf{n}}}}\times {{{\bf{a}}}}\\ {{{{\bf{m}}}}}_{{SV}}={{{\bf{n}}}}\times \left({{{\bf{n}}}}\times {{{\bf{a}}}}\right)\end{array}\right.$$

The effective shear moduli ${\mu }_{A},{\mu }_{B}$ and ${\mu }_{C}$, reflecting both intrinsic material stiffness and the current prestrain (stretch ratio λ), are defined as:

2 $$\left\{\begin{array}{c}{\mu }_{A}=2{\lambda }^{2}{C}_{10}+2\lambda {C}_{01}\\ {\mu }_{B}=\frac{2}{\lambda }{C}_{10}+2\lambda {C}_{01}\\ {\mu }_{C}=\frac{2}{\lambda }{C}_{10}+\frac{2}{{\lambda }^{2}}{C}_{01}\end{array}\right.$$

In reverberant shear wavefields composed of multiple propagation directions and polarizations, the superposition of component waves leads to complex spatial patterns. These patterns pose significant challenges to conventional modal decomposition, particularly in inhomogeneous biological tissues.

To overcome these challenges, a differential-operator-based inversion framework was formulated to directly extract stress information from the composite wavefield. The fundamental mathematical rationale of this framework is to avoid solving the complex, nonlinear wave equations under large deformation. Instead, we leverage the known analytical characteristics of wave propagation, e.g., polarization and velocity modulation. In addition, a specific set of operators are also used to analytically “substitute” the unknown terms, such as local propagation directions and modulation angles, within the reverberant field. Physically, these operators exploit the vector identities between the wavefield ${{{\boldsymbol{u}}}}$ and the stress axis ${{{\bf{a}}}}$. Since SH polarization ${{{{\bf{m}}}}}_{{SH}}$ is inherently orthogonal to ${{{\bf{a}}}}$ (${{{\bf{a}}}}{{{\boldsymbol{\cdot }}}}{{{{\bf{m}}}}}_{{SH}}=0$), whereas SV polarization ${{{{\bf{m}}}}}_{{SV}}$ has a projection onto ${{{\boldsymbol{a}}}}$ that varies with the propagation direction, operators involving (${{{\bf{a}}}}{{{\boldsymbol{\cdot }}}}{{{\bf{U}}}}$) effectively act as polarization filters to differentiate the two modes. Furthermore, directional derivatives of the form ${\left({{{\boldsymbol{\nabla }}}}\cdot {{{\bf{a}}}}\right)}^{2}$ incorporate angular weighting factors equivalent to ${\cos }^{2}\theta$ for subwave components. By applying different orders of these derivatives, the operators generate distinct, linearly independent angular kernels for the effective moduli ${\mu }_{A}$, ${\mu }_{B}$ and ${\mu }_{C}$.

Three kinds of key operators, as detailed in Supplementary Note 2, were constructed to address the wavefield complexity: 1) Polarization Manipulation ${{{\boldsymbol{\nabla }}}}\times {{{\bf{U}}}}$: Enabling SV-SH polarization conversion to isolate stress-dependent interactions. 2) Component-Selective Filtering ${{{\bf{a}}}}\cdot {{{\bf{U}}}}$: Specifically suppressing SH-wave components and enhancing SV-wave fidelity. 3) Angle-Domain Weighting Modulation ${\left({{{\boldsymbol{\nabla }}}}\cdot {{{\bf{a}}}}\right)}^{2}$ and ${\left|\left({{{\boldsymbol{\nabla }}}}\times {{{\bf{a}}}}\right)\right|}^{2}$: Performing weighted wavefield reconstruction via the θ parameter to modulate sensitivity directionally. Conceptually, these operators act on the reverberant field similar to calibrated rotations used to solve a Rubik’s Cube: while each operator induces distinct, synchronized changes across all wavelets based on their specific orientations, their structured combination mathematically untangles the chaotic interference into a solvable analytical relationship. This allows for the local decoupling of the intermediate variables ${\mu }_{A}$, ${\mu }_{B}$ and ${\mu }_{C}$, which serve as essential mathematical bridges, to yield a concise, closed-form expression for the uniaxial stress magnitude without requiring explicit wave-mode separation or global wave steering.

These operators form a structured operator complex that enables explicit reconstruction of the heterogeneous stress:

3 $$\left\{\begin{array}{c}-\rho {\omega }^{2}{{{\bf{U}}}}={\mu }_{A}\cdot {{{{\bf{L}}}}}_{1}+{\mu }_{B}\cdot {{{{\bf{L}}}}}_{2}+{\mu }_{C\cdot }\left({{{{\bf{L}}}}}_{3}-{{{{\bf{L}}}}}_{2}\right)\\ {{{{\bf{L}}}}}_{1}={\left({{{\boldsymbol{\nabla }}}}\cdot {{{\bf{a}}}}\right)}^{2}{{{\bf{U}}}}\\ {{{{\bf{L}}}}}_{2}=-\left({{{\bf{a}}}}\cdot \left({{{\boldsymbol{\nabla }}}}\times {{{\bf{U}}}}\right)\right)\cdot \left({{{\boldsymbol{\nabla }}}}\times {{{\bf{a}}}}\right)\\ {{{{\bf{L}}}}}_{3}={\left|\left({{{\boldsymbol{\nabla }}}}\times {{{\bf{a}}}}\right)\right|}^{2}{{{\bf{U}}}}\end{array}\right.$$

Here, ${{{\bf{U}}}}$ represents the observed 3D displacement field, ${{{{\bf{L}}}}}_{i}$ represents the applied operators. This inversion framework is agnostic to specific propagation direction or wave mode combinations, thus suitable for reverberant wavefields in complex biological tissues.

Because real tissues exhibit some compressibility—and thus can support longitudinal waves—we apply the curl operator to both sides of Eq. (3) and use a sliding 4 × 4 × 4 voxel window for inversion. In regions where the principal stress direction ${{{\boldsymbol{a}}}}$ can be determined (e.g., via anatomical alignment or geometric inference), this operator yields the effective moduli ${\mu }_{A},{\mu }_{B}$ and ${\mu }_{C}$. From these values, the uniaxial stress magnitude σ and stretch ratio λ are analytically recovered (detailed in Supplementary Note 1):

$$\sigma={\mu }_{A}-{\mu }_{C}$$

4 $$\lambda={\left(\frac{{\mu }_{A}}{{\mu }_{C}}\right)}^{1/3}$$

This framework establishes an analytical relationship between three key biomechanical properties: constitutive parameters, local strain state, and stress distribution. Through voxel-level inversion across the entire tissue volume, we generate high-resolution stress maps that enable precise spatial identification of mechanically stressed regions, including tumor margins and intracranial pressure gradients. The quasi-local homogeneity assumption provides an optimal compromise between physical fidelity and computational stability, facilitating robust stress imaging in complex biological systems.

### Simulation setup for hemispherical shell and embedded sphere models

All simulations were implemented in COMSOL Multiphysics v6.2 by modeling incompressible Mooney–Rivlin solids and employing a uniform 3 mm mesh resolution for both geometry and wave-field sampling. In all cases, the stress field was first established during a quasi-static loading stage, where the vector ${{{\bf{a}}}}$ is defined as the normalized eigenvector corresponding to the third principal stretch ${\lambda }_{3}$. This stress field was then probed via low-amplitude dynamic shear excitations; surface displacements were recorded subsequently and used for inversion calculations based on the analytical relations given in Eqs. (3–4).

For the hemispherical shell model, a hollow shell with an inner diameter of 6 cm and an outer diameter of 12 cm was parameterized with ${C}_{01}$ = 400 Pa and ${C}_{10}$ = 600 Pa. Hydrostatic pressure was applied to the inner surface in 100 Pa increments from 100 Pa to 1200 Pa, producing large, radially graded deformations and a corresponding stress distribution. After each deformation step, a small-amplitude shear excitation was imposed around the deformed rim; reverberant shear waves propagating through the stressed medium were recorded on the shell surface. These displacement fields were then processed via the prescribed inversion framework to recover local stress values, enabling direct comparison against the known load history.

In the embedded-sphere block model, a 10 cm×10 cm×8 cm hyperelastic block (${C}_{01}$ = 500 Pa and ${C}_{10}$ = 700 Pa) housed a central spherical cavity of radius 2 cm. To impose heterogeneous stress, the bottommost cavity node was fixed while all other nodes were radially expanded by a factor of (k+1), yielding a nonuniform stress field in the surrounding matrix. Once the block reached its deformed configuration, 50 Hz shear excitations were applied to three orthogonal faces, and the resulting wave-induced displacements were recorded. For inversion, the 3 mm-sampled data were analyzed using two stress-direction strategies: true tensor orientations from the simulation and unit normals to the deformed cavity surface. Comparing reconstructed and ground-truth maps demonstrated the framework’s ability to resolve spatially varying stress under both ideal and geometry-based direction assignments.

### Experimental setup for spherical indentation in FEM modeling and MRE measurements

The experimental design consisted of a rigid plastic container filled with cylindrical gelatin phantoms, subjected to localized indentation via a rigid hemispherical shell to generate spatially heterogeneous stress fields. Two indentation depths (12 mm and 18 mm) were applied to simulate different levels of stress (Fig. 4a). The full experimental pipeline included finite element (FEM) modeling, shear wave excitation and acquisition, and MRE-based stress inversion. For the inversion of both FEM and MRE data, the vector ${{{\boldsymbol{a}}}}$ is uniformly defined as the normalized eigenvector corresponding to the third principal stretch ${\lambda }_{3}$ from FEM simulations.

In the FEM simulation, two cylindrical gelatin models (radius: 56 mm; heights: 82 mm and 88 mm) were constructed in Abaqus 2022, using an incompressible Mooney–Rivlin material model (${C}_{01}$ = 150 Pa, ${C}_{10}$ = 100 Pa). The bottom surface was fixed, and the lateral surfaces were assigned roller boundary conditions. A rigid hemispherical shell with a radius of 50 mm was used to apply vertical displacements of 12 mm and 18 mm, respectively, generating corresponding pre-deformation fields. These pre-deformed geometries were then imported into COMSOL Multiphysics 6.2, where a 50 Hz planar shear wave excitation with amplitude $[1,\,1.5,\,0]$ N/m² was applied to the bottom and side walls. Three-dimensional displacement responses were recorded at a spatial resolution of 3 mm.

For MRE measurements, gelatin phantoms were prepared by mixing gelatin, glycerol, and water at a mass ratio of 1:13.5:14.2. The mixture was poured into the same plastic container to form cylindrical samples with heights of 82 mm and 88 mm. A rigid plastic hemispherical shell (diameter: 50 mm) was used for indentation. To minimize susceptibility-induced MRI artifacts at the gel boundary and reduce undesired shear stress during loading, a thin layer of water was applied at the contact interface between the shell and the gel surface. Shear waves at 50 Hz were introduced from the container base using an electromagnetic actuator^65^. A 3 T MRI scanner (uMR790, United Imaging Healthcare) was used to acquire 2D GRE-MRE images (TR/TE = 1100/18 ms; MEG = 30 mT/m), capturing four phase-offset frames at 3 mm isotropic resolution. Phase unwrapping was performed using a Dual-DC algorithm^66^, and the resulting complex displacement fields were Gaussian-filtered in 3D (*σ*^2^ = 1) to suppress noise prior to inversion.

Due to uncertainty in constitutive parameters under dynamic loading conditions in real tissues, FEM-derived prestrain distributions (stretch ratio λ) were used as the ground truth reference. Stress and stretch fields were reconstructed from both FEM and MRE wave data using the proposed wave-based inversion framework (Eqs. 3–4).

### In vivo mapping of brain tissue stress using MRE

To achieve in vivo quantification of brain tissue stress under physiological and pathological conditions, we established a standardized MRE workflow incorporating common acquisition procedures and pathology-specific modifications. All human experiments were conducted using a 3 T clinical MRI scanner (uMR790, United Imaging Healthcare), employing a harmonized imaging protocol that included high-resolution T1-weighted structural imaging and a 2D spin-echo echo-planar imaging (SE-EPI) MRE sequence (TR/TE = 4000/65 ms, motion-encoding gradient amplitude = 40 mT/m, vibration frequency = 50 Hz). Shear waves were introduced via a neck-mounted electromagnetic actuator^65^. For each participant, eight phase-offset images were acquired at an isotropic spatial resolution of 3 mm. Full-field complex displacement fields were reconstructed using the Dual-DC phase unwrapping algorithm^66^.

To enhance inversion robustness and minimize noise-induced artifacts, the displacement data underwent a two-stage preprocessing procedure. First, a three-dimensional directional Gaussian filter (*σ*^2^ = 0.5) was applied to reduce spatial noise, followed by a Butterworth low-pass filter (normalized cutoff frequency = 0.3) to suppress high-frequency disturbances. This preprocessing pipeline was consistently applied across all human in vivo datasets, ensuring comparability and stability of the inversion results. The stress inversion procedure also relies on the analytical framework described in Eqs. 3–4.

We then implemented pathology-specific strategies for estimating the stress direction field within each cohort. For patients with brain tumors, including meningiomas and pituitary adenomas, tumor boundaries were manually delineated by experienced neuroradiologists on T1-weighted images. A smooth 3D tumor surface was reconstructed via fifth-order polynomial surface fitting. This directional field was treated as the principal stress orientation ${{{\boldsymbol{a}}}}$ and used to guide the inversion process. A 1.5 cm (5 pixels) isotropic peritumoral shell was defined as the region of interest (ROI), within which the local stress direction was assumed to be normal to the tumor surface (Supplementary Fig. 9). This relatively broad shell was chosen to encompass both immediate peritumoral tissue and the surrounding mechanically perturbed zones, ensuring inclusion of stress-transmitting interfaces while avoiding tumor-core artifacts. This width was specifically chosen to match the large physical scale of the meningiomas in our cohort (diameters 3.96–6.06 cm), ensuring the inclusion of the broader mechanically perturbed zones while maintaining statistical robustness for the inversion.

For pituitary adenomas, additional emphasis was placed on the biomechanical impact on the optic chiasm. A conservative 6 mm (2-pixel) peritumoral shell was delineated around the superior tumor margin in the coronal plane to ensure coverage of the optic chiasm region. The reduced shell thickness is specifically tailored to the compact anatomy of the optic chiasm, minimizing partial volume effects from non-visual structures. Within this defined zone, we computed stress distributions separately for the left and right region-of-interest (ROI) to facilitate correlation with laterality-specific clinical deficits. Analysis was confined to the superior half of the ROI in the coronal plane, aligning with the tumor region exerting compression on the optic chiasm. The mean stress values from the left and right subregions were then independently correlated with corresponding clinical assessments of left and right eye function. Visual acuity was evaluated using standardized Snellen charts, following the international protocol for visual acuity assessment recommended by the World Health Organization^67^. In parallel, visual field sensitivity and defects were quantitatively assessed using automated static perimetry^68^. This bilateral analysis allowed us to directly examine the mechanobiological relevance of localized stress alterations in relation to lateralized visual dysfunctions.

In the ICP measurement cohort, we defined a 3-voxel (9 mm) inward shell from the cortical surface to capture the stress distribution across the brain’s outer layer. This depth was optimized to focus on the brain-CSF interface, which can be analyzed using the proposed model. Brain masks were obtained via FSL skull-stripping, and the cortex was treated as an elastic boundary. At this interface, stress continuity links the brain tissue stress state to ICP: the hydrostatic pressure of the CSF is balanced by the normal stress of the adjacent brain tissue. As demonstrated numerically in Supplementary Note 5, our inversion scheme is insensitive to the hydrostatic component and specifically recovers the deviatoric normal stress, which provides a direct estimate of global ICP. Stress directions were assumed to follow the inward surface normals, and the mean stress magnitude within this cortical shell served as a surrogate for global ICP^46^, thereby minimizing the influence of local modeling mismatches.

Cognitive function was evaluated using the Mini-Mental State Examination (MMSE)^69^ and Montreal Cognitive Assessment (MoCA)^70^, with MCI defined by MoCA < 26 and MMSE ≥ 24. Diagnoses of normal-pressure hydrocephalus (NPH) were confirmed by experienced clinicians based on neuroimaging and clinical signs. Group comparisons employed Shapiro–Wilk tests for normality followed by two-tailed independent *t* tests (*p* < 0.05).

This integrated MRE-based framework enables flexible yet standardized stress mapping across multiple clinical contexts. By incorporating anatomical geometry, wave dynamics, and pathology-specific constraints, the method offers a non-invasive, reproducible platform for probing the mechanical state of the brain under both normal and disease conditions.

### Software and hardware platform

The MR data processing, TWE dataset generation and stress inversion method were implemented with MATLAB R2022a (MathWorks, Natick, MA, USA) on macOS Ventura 13.5 operating system equipped with an Apple M1 Pro (16 GB memory) chip. All the Abaqus and COMSOL simulations were implemented on the Siyuan-1 cluster (2 x Intel Xeon ICX Platinum 8358 (2.6 GHz, 32 cores), 16 x 32GB TruDDR4 3200 MHz) of the Center for High Performance Computing at Shanghai Jiao Tong University.

### Ethics statement

This study was approved by the Shanghai Jiao Tong University Ethics Committee (E2021119I, E20250528I). All procedures involving human participants were conducted in accordance with the Declaration of Helsinki and relevant institutional guidelines. Written informed consent was obtained from all participants prior to participation. All MRI data were anonymized prior to analysis.

### Reporting summary

Further information on research design is available in the Nature Portfolio Reporting Summary linked to this article.

## Supplementary information

Supplementary Information

Description of Additional Supplementary Files

Supplementary Movie 1

Reporting Summary

Transparent Peer Review file

## Source data

Source Data

## Data Availability

Simulation data and MRE wave field data for a meningioma case have been deposited on Zenodo^71^. Source data are provided in this paper.

## Code availability

Processing scripts for the shared datasets, illustrating the reconstruction workflow, are publicly available at the same repository^71^. Other code and information are available from the corresponding author (Y.F.) upon request.
