# Supplementary material for: Noninvasive 3D mapping of tissue stress using reverberant shear wave
Source: Nat Commun. 2026 Aug 11;17:9626. doi: 10.1038/s41467-026-76509-0 (PMC13554193; doi:10.1038/s41467-026-76509-0)
Supplement: Supplementary file 1 [file 41467_2026_76509_MOESM1_ESM.pdf]

Supplementary Information

The file includes:

Supplementary Figures. S1 to S11

Supplementary notes S1 to S9

References

Supplementary Figures

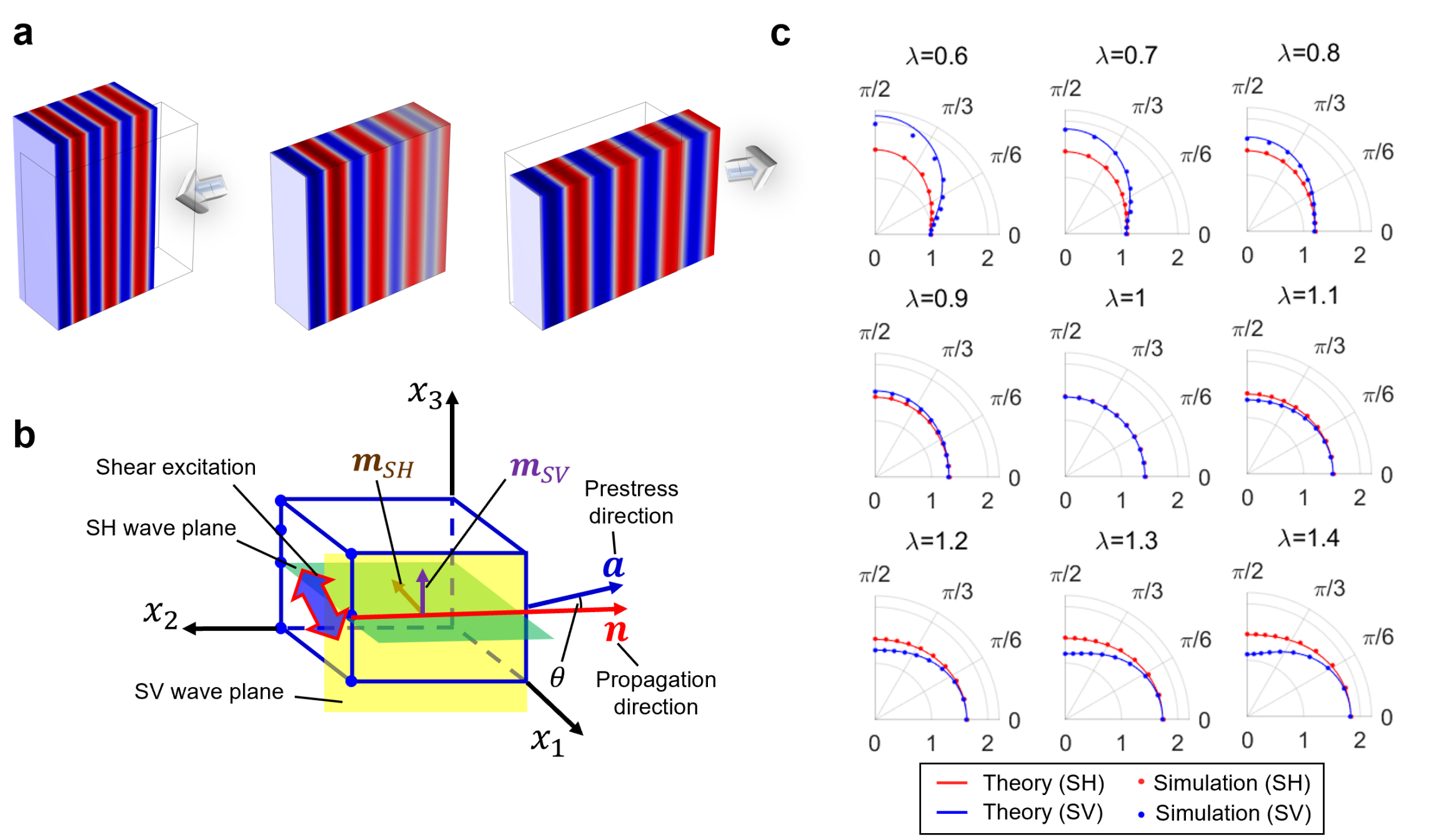

Supplementary Fig. 1 Simulation validation of wave speed characteristics. **a** Wavefield distributions under varying pre-stress applied along the y-axis. **b** Experimental setup schematic showing pre-stress direction, excitation loading, and polarization directions of SH/SV waves. **c** Theoretical (solid lines) versus simulated (points) SH/SV wave speeds across stretch ratios. The polar plot displays wave speed magnitude (m/s, radial axis) versus propagation angle relative to pre-stress (θ, polar axis).

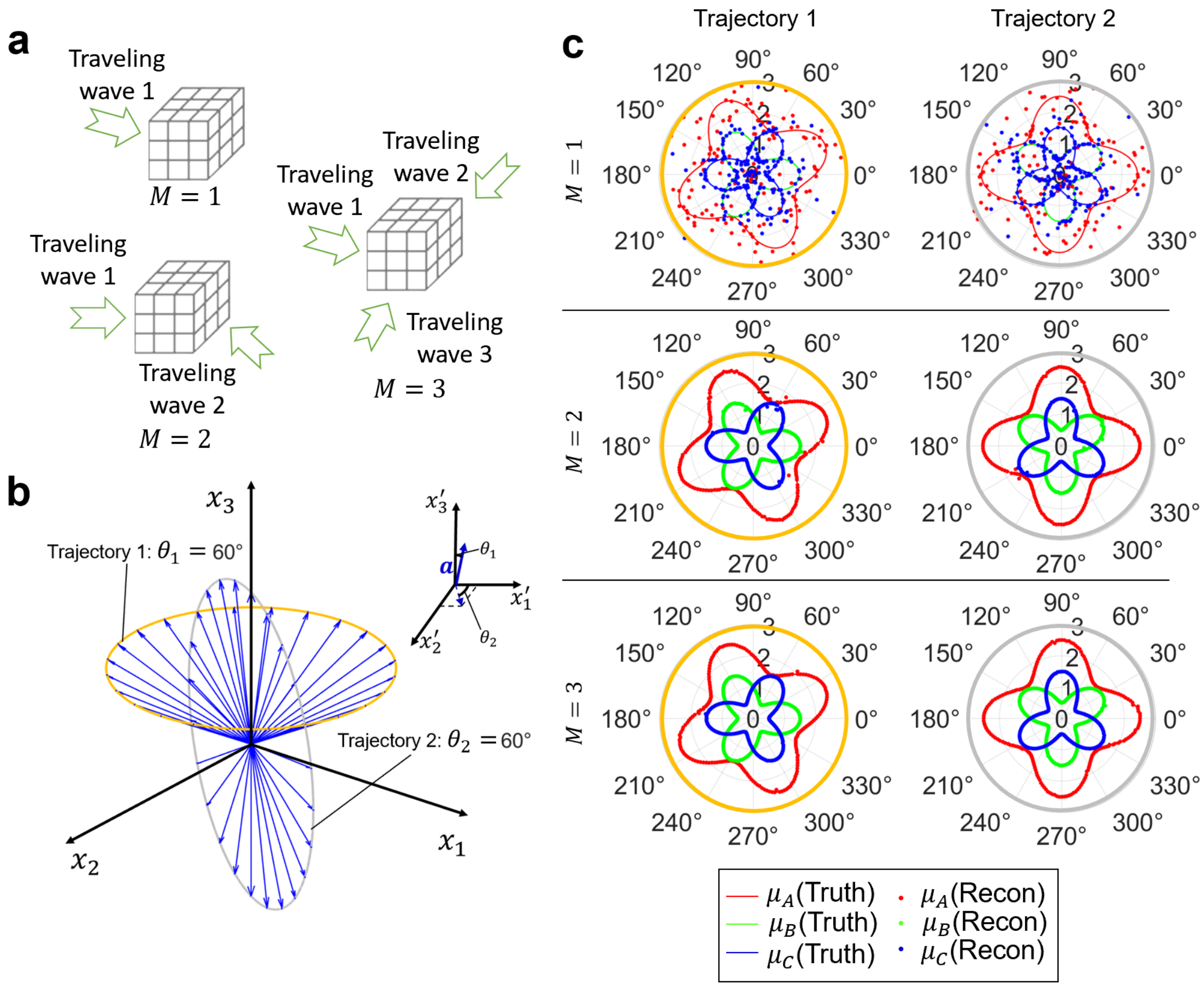

Supplementary Fig. 2: Validation of local simulated wavefield inversion performance. a Three types of wavefields were constructed within a 3×3×3 mm³ cubic region with 1 mm resolution, each composed of randomly oriented traveling waves at 30 Hz, with different numbers of propagation directions ($M = 1/2/3$). **b** Simulation of prestress directions: different prestress directions were simulated using direction vectors defined by angles θ_1 and θ_2. Two trajectories were considered: trajectory 1 (yellow) varies θ_2 continuously while holding θ_1 constant at $\frac{\pi}{3}$; trajectory 2 (gray) varies θ_1 continuously while keeping θ_2 fixed at $\frac{\pi}{3}$. For each trajectory, 200 test sample points were collected. **c** Estimated versus ground truth material parameters are visualized in polar coordinates, where the angular component corresponds to the directional parameters defined in **b**, and the radial scale is in kilopascals (kPa). Different marker symbols represent results from different wavefield types and sampling trajectories.

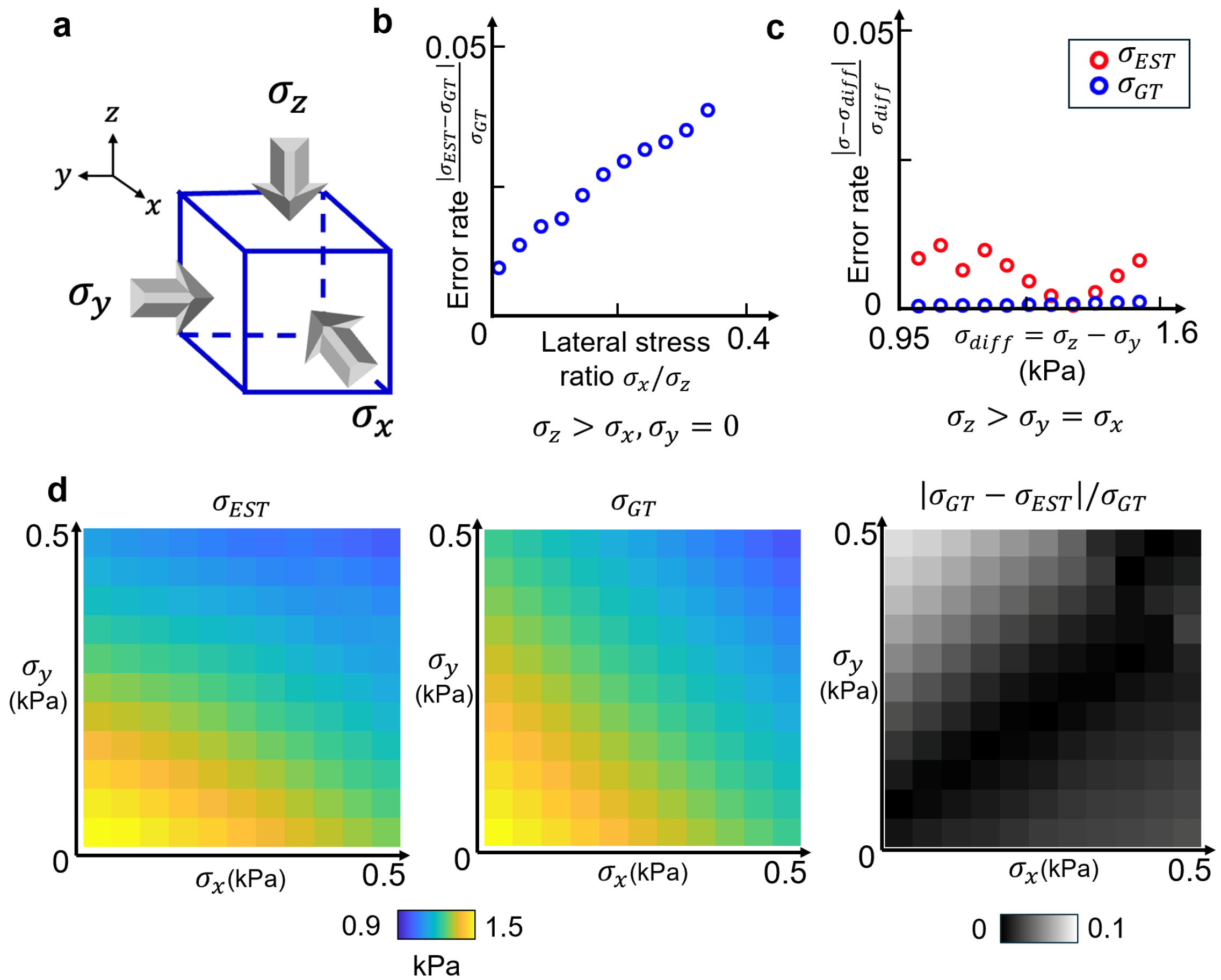

Supplementary Fig. 3. Validation of the stress estimation under multiaxial stress conditions. **a** A 50×50×50 mm^3 cube with triaxial compressive loads ($\sigma_x, \sigma_y, \sigma_z$). A Mooney-Rivlin material model was used. **b** 2D Multiaxial Case**:** Estimation error for the stress as a function of lateral stress ratio σ_x/σ_z. **c** 3D Symmetric Case: Relative error of the estimated stress (σ_{EST}, red dots) and the stretch-derived stress (σ_{GT}, blue dots) compared to the theoretical deviatoric difference ($\sigma_{diff} = \sigma_{GT} - \sigma_y$). **d** Full 3D Asymmetric Case: Distribution of estimated stress across a comprehensive sweep of independent σ_x and σ_y (0–500 Pa).

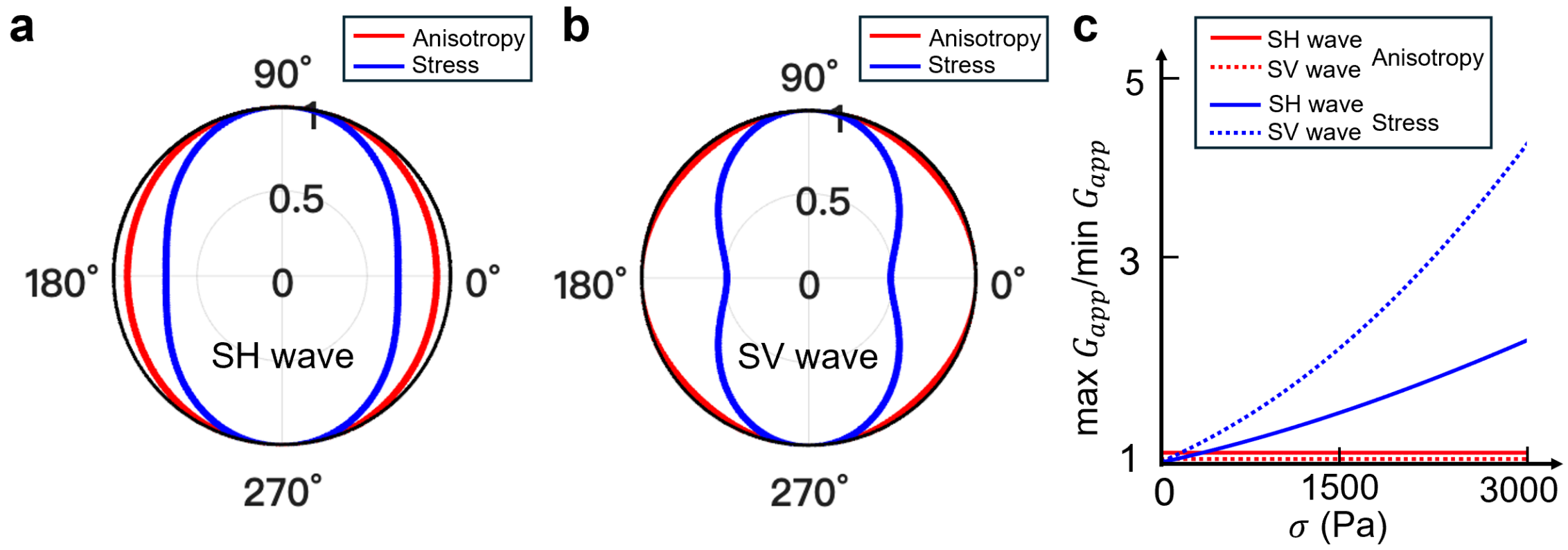

Supplementary Fig. 4: Comparison of the apparent shear modulus ($G_{app} = \rho c_{app}^2$) due to applied stress versus intrinsic mechanical anisotropy. a, b Normalized apparent shear modulus as a function of the angle between wave-propagation direction and the fiber/principal-stress axis, shown for **(a)** SH and **(b)** SV waves. **c** Ratio of maximum to minimum G_{app} ($G_{app,max}/G_{app,min}$) under increasing applied stress, using an anisotropic material without applied stress as the baseline. The pronounced increase in the ratio confirms that applied stress, rather than intrinsic mechanical anisotropy, is the dominant source of wave-speed variation.

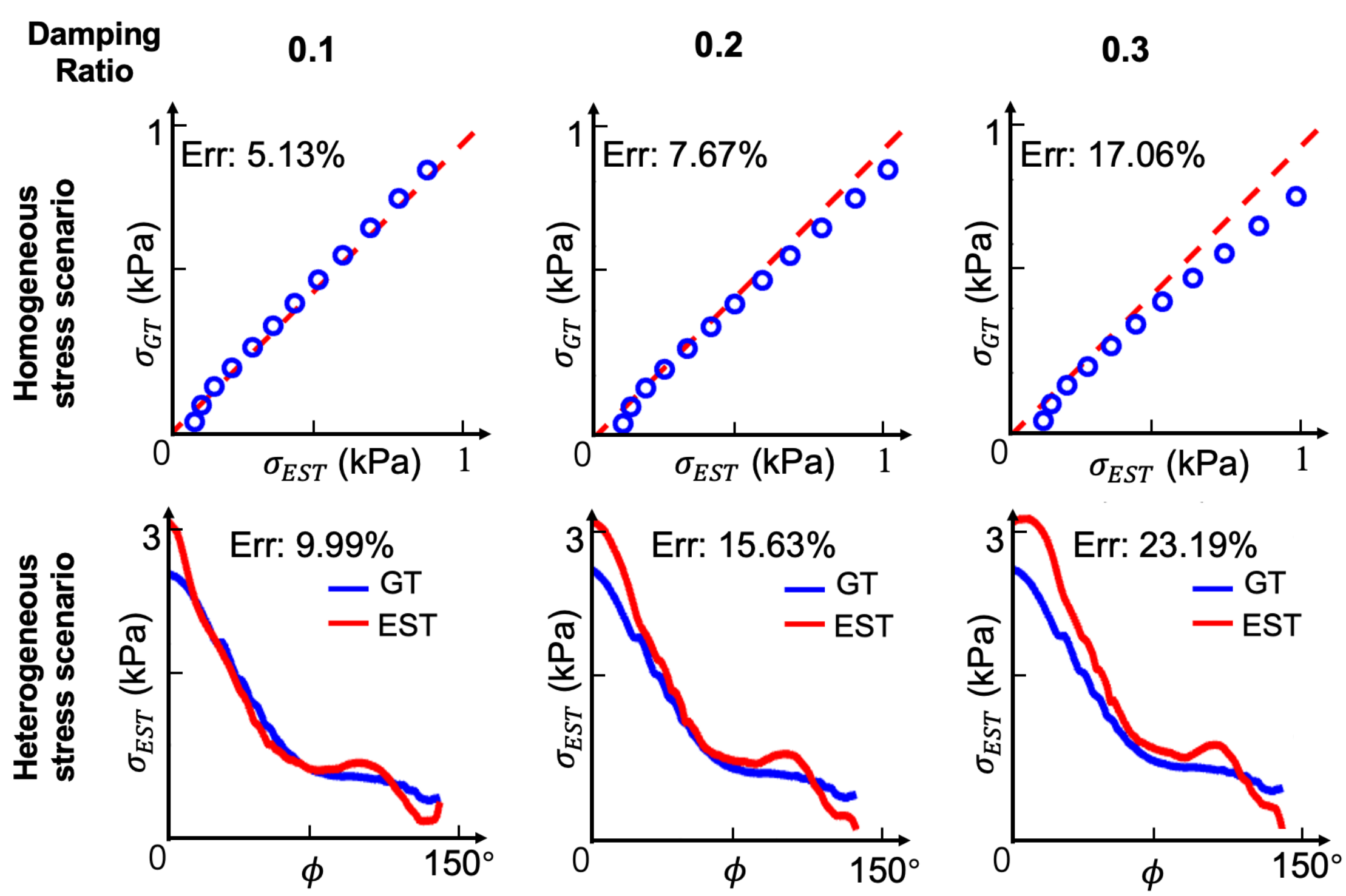

Supplementary Fig. 5 Stress estimation under visco-hyperelastic material modeling. A generalized Maxwell viscoelastic model (single branch, τ = 3 ms) is coupled with the Mooney-Rivlin hyperelastic framework. Three loss tangent values (damping ratio G'/G''= 0.1, 0.2, 0.3) are tested. Top row (Fig. 2): Pressurized hemispherical shell simulation. Scatter plots compare ground-truth stress (y-axis) with estimated stress (x-axis). The red dashed line indicates the ideal 1:1 correspondence. Bottom row (Fig. 3): Heterogeneous embedded-sphere inflation simulation. Stress estimation error increases monotonically (9.99% to 23.19%), while the spatial stress pattern is well preserved. Together, these results confirm the robustness of the inversion framework to physiologically relevant levels of viscoelastic damping.

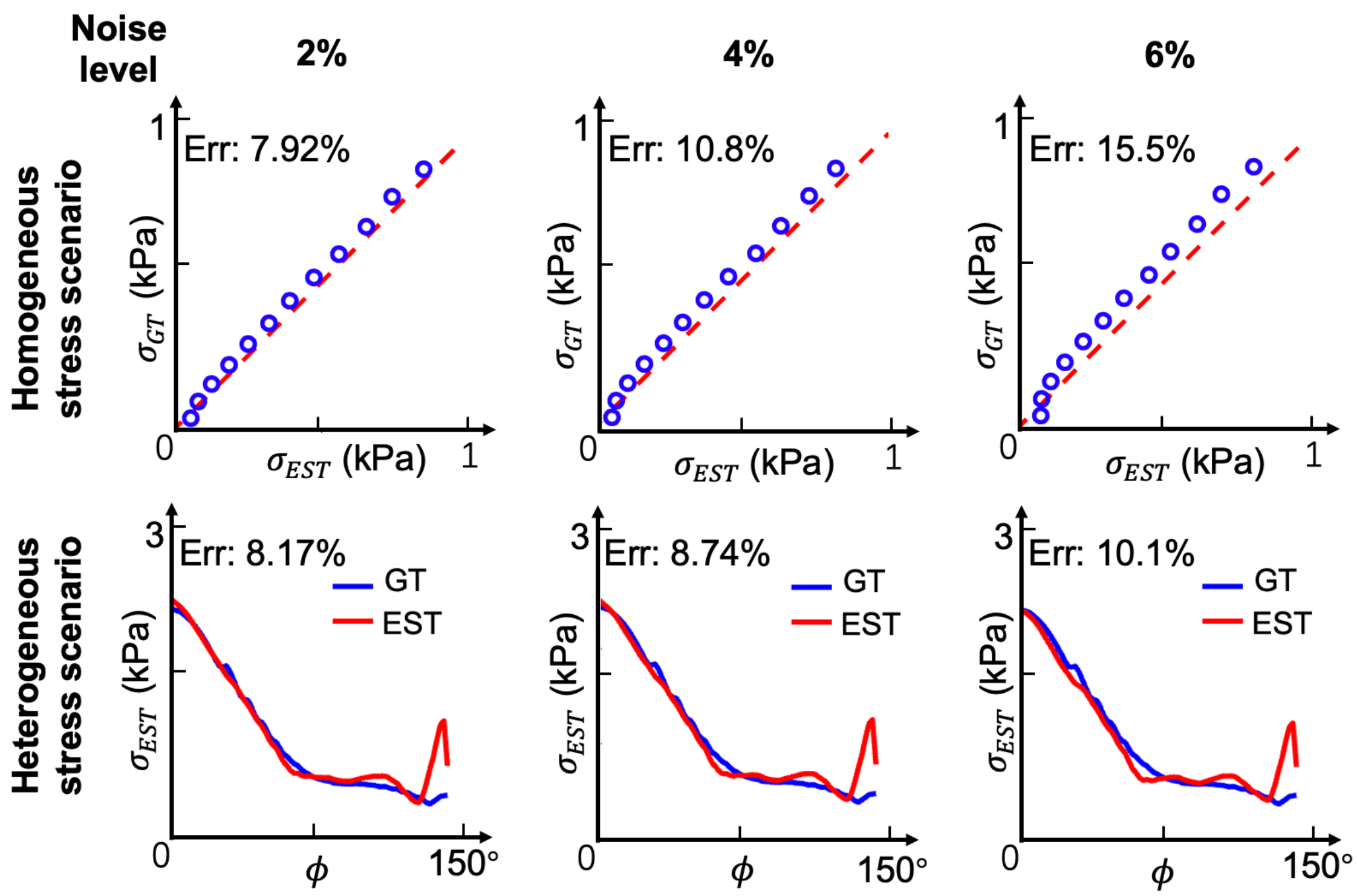

Supplementary Fig. 6 Stress estimation under increasing measurement noise. Top row (**Fig. 2**): Pressurized hemispherical shell simulation. Scatter plots compare ground-truth stress (x-axis) with estimated stress (y-axis). The red dashed line indicates the ideal 1:1 correspondence (x = y). Despite rising noise, estimation error increases only moderately (7.92% to 15.5%), while the linear correlation with ground truth remains strong. Bottom row (Fig. 3): Heterogeneous embedded-sphere inflation simulation. Stress estimation error remains highly stable, even under 6% noise. Together, these results demonstrate the robustness of the inversion algorithm to realistic levels of measurement noise.

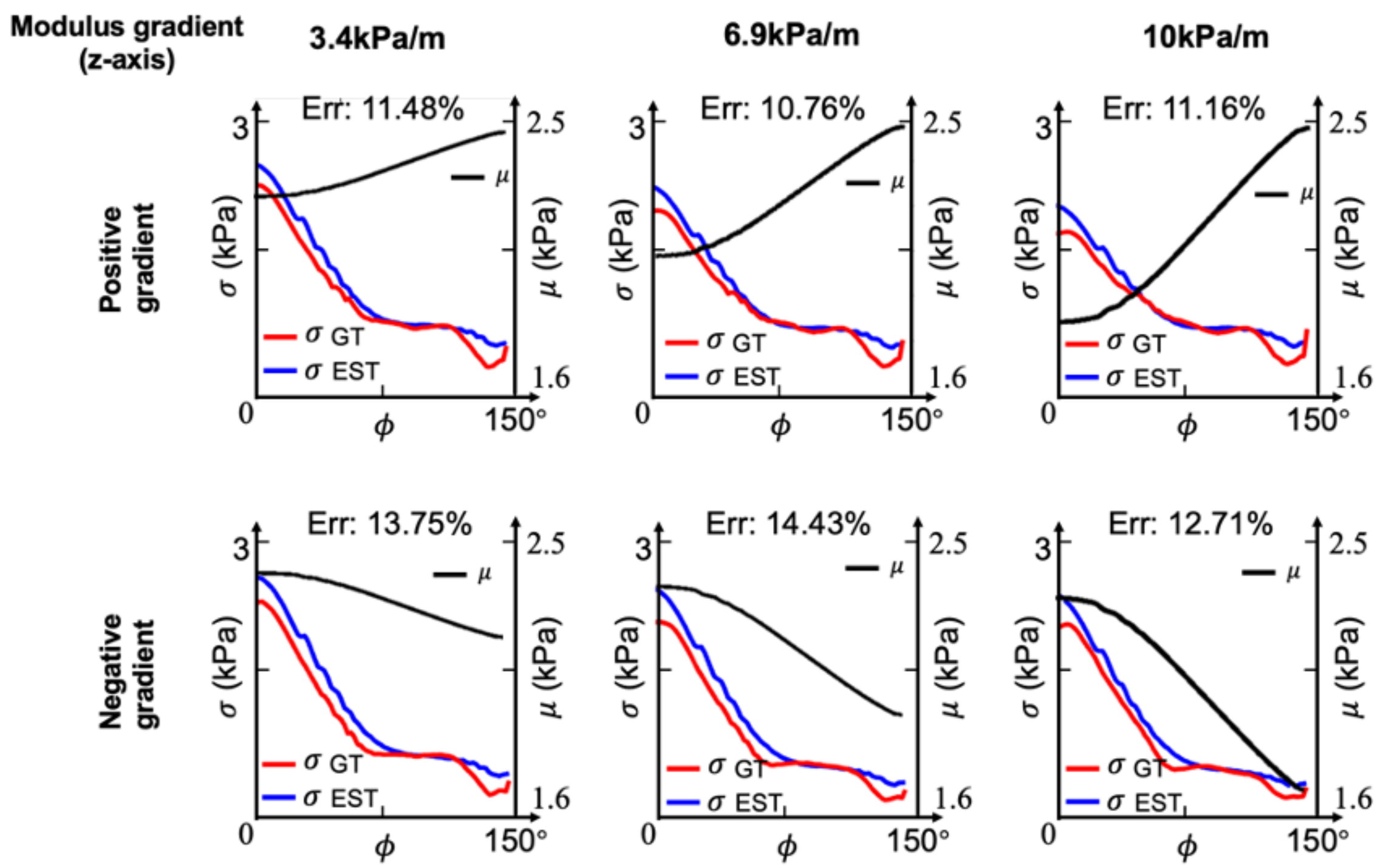

Supplementary Fig. 7. Stress estimation accuracy in spatially heterogeneous media with linearly graded material properties. The simulation is based on the embedded-sphere inflation model (Fig. 3), with the shear modulus $\mu(z) = 2[C_{10}(z) + C_{01}(z)]$ varying linearly along the z-axis. Three modulus gradients (3.4, 6.9, and 10 kPa/m) are tested under positive-gradient (top row) and negative-gradient (bottom row) conditions. In each panel, σ_{GT} (red) and σ_{EST} (blue) denote the ground-truth and estimated stress as functions of elevation angle φ; the black curve shows the local shear modulus μ (right axis).

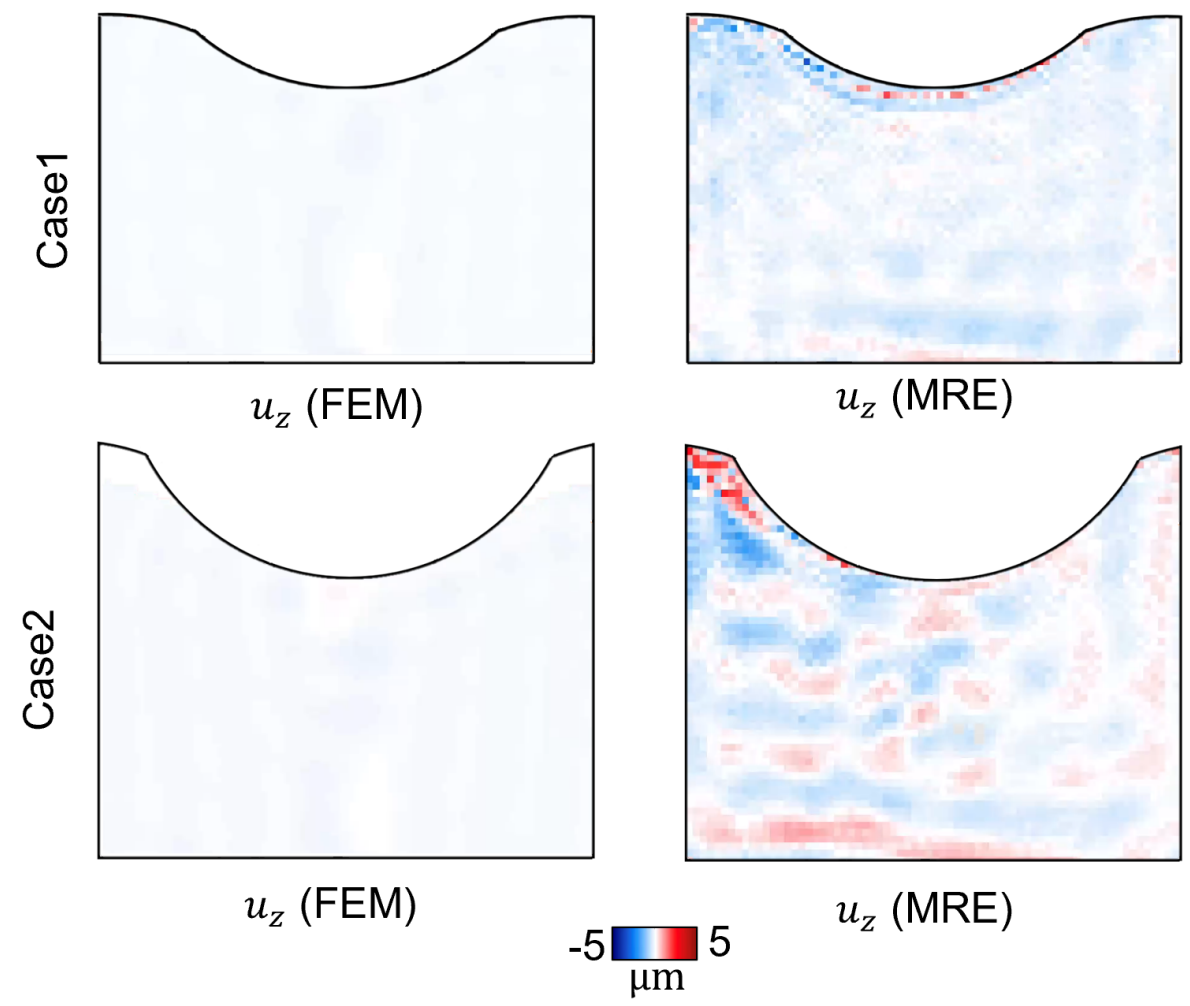

Supplementary Fig. 8: Small-amplitude shear wave fields propagating in the z-direction on the Y–Z cross-section, obtained by both FEM and MRE during spherical indentation tests on a gelatin phantom under two loading conditions.

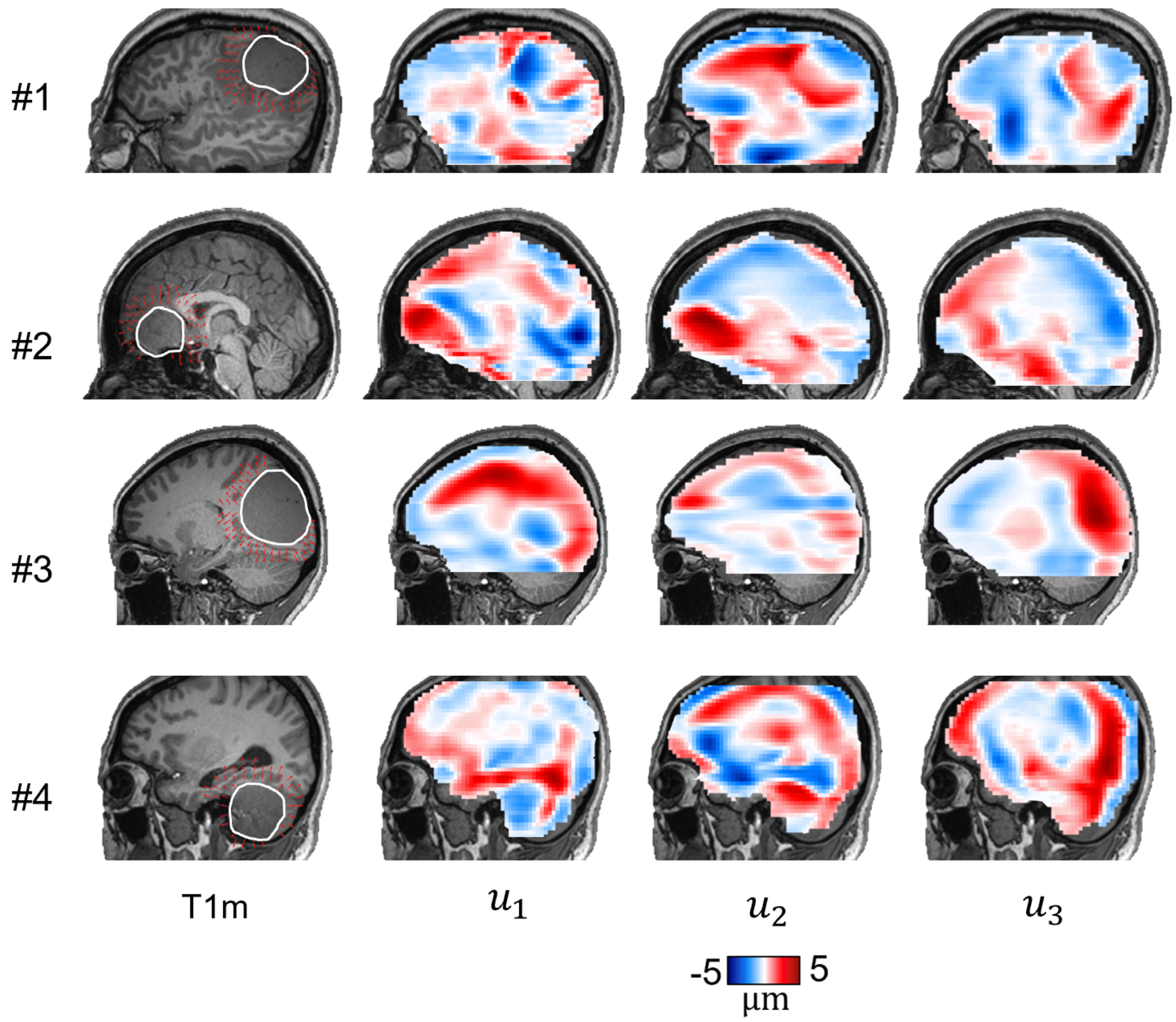

Supplementary Fig. 9 Sagittal T1 maps at four distinct meningioma sites, with white outlines delineating the tumor boundaries and arrows indicating the prestress direction normal to the tumor surface. For each case, the corresponding whole brain shear wave fields measured by MRE along the three encoding directions are also shown. This figure corresponds to Fig. 5.

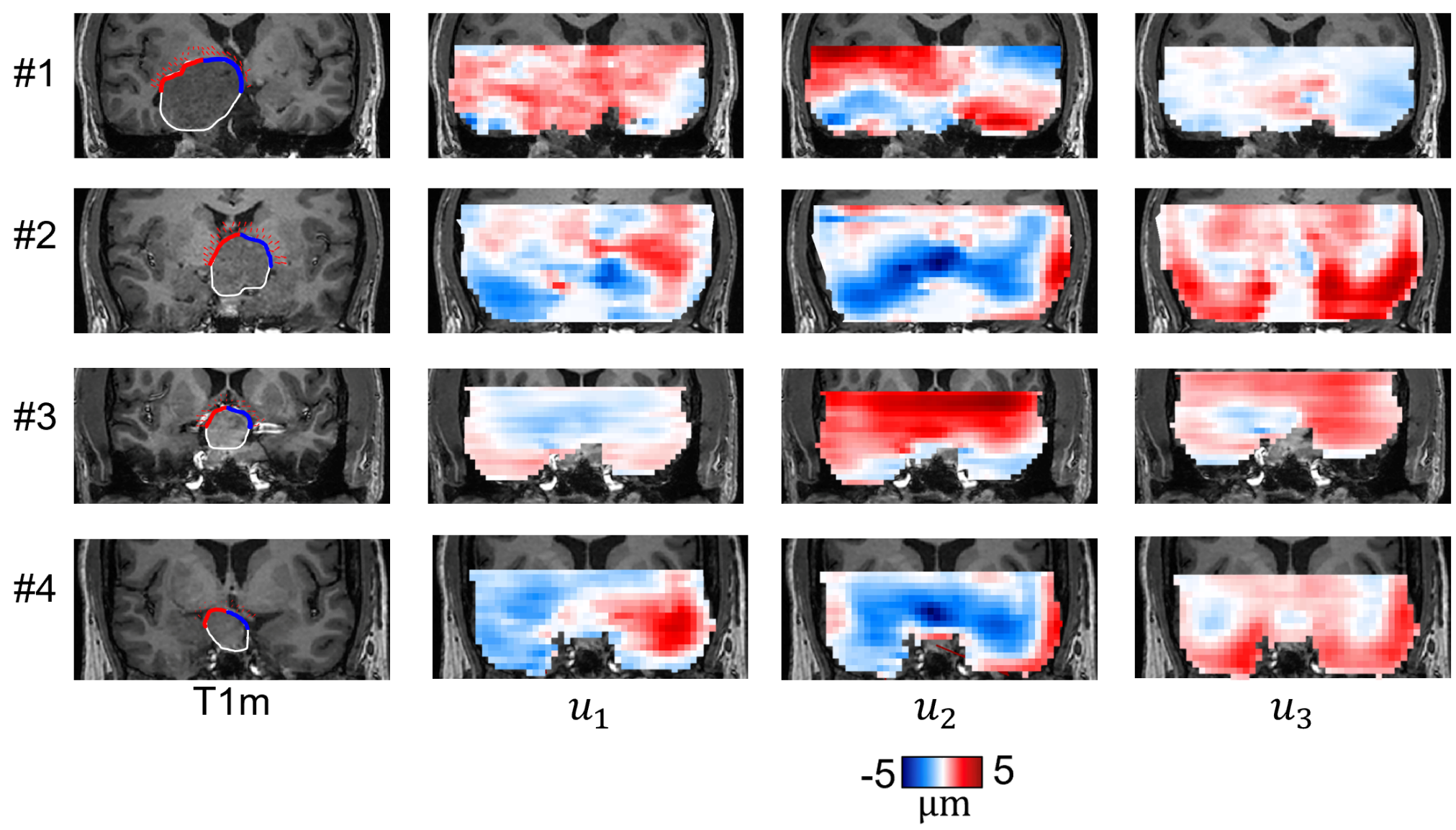

Supplementary Fig. 10 Coronal T1 maps for four patients with pituitary adenomas are shown, with white lines delineating tumor boundaries, red lines highlighting the actual right-side prestress ROI and blue lines marking the left-side prestress ROI. Arrows indicate the prestress direction normal to the tumor surface. The corresponding shear wave fields measured by MRE along the three encoding directions are also presented. This figure corresponds to Fig. 6.

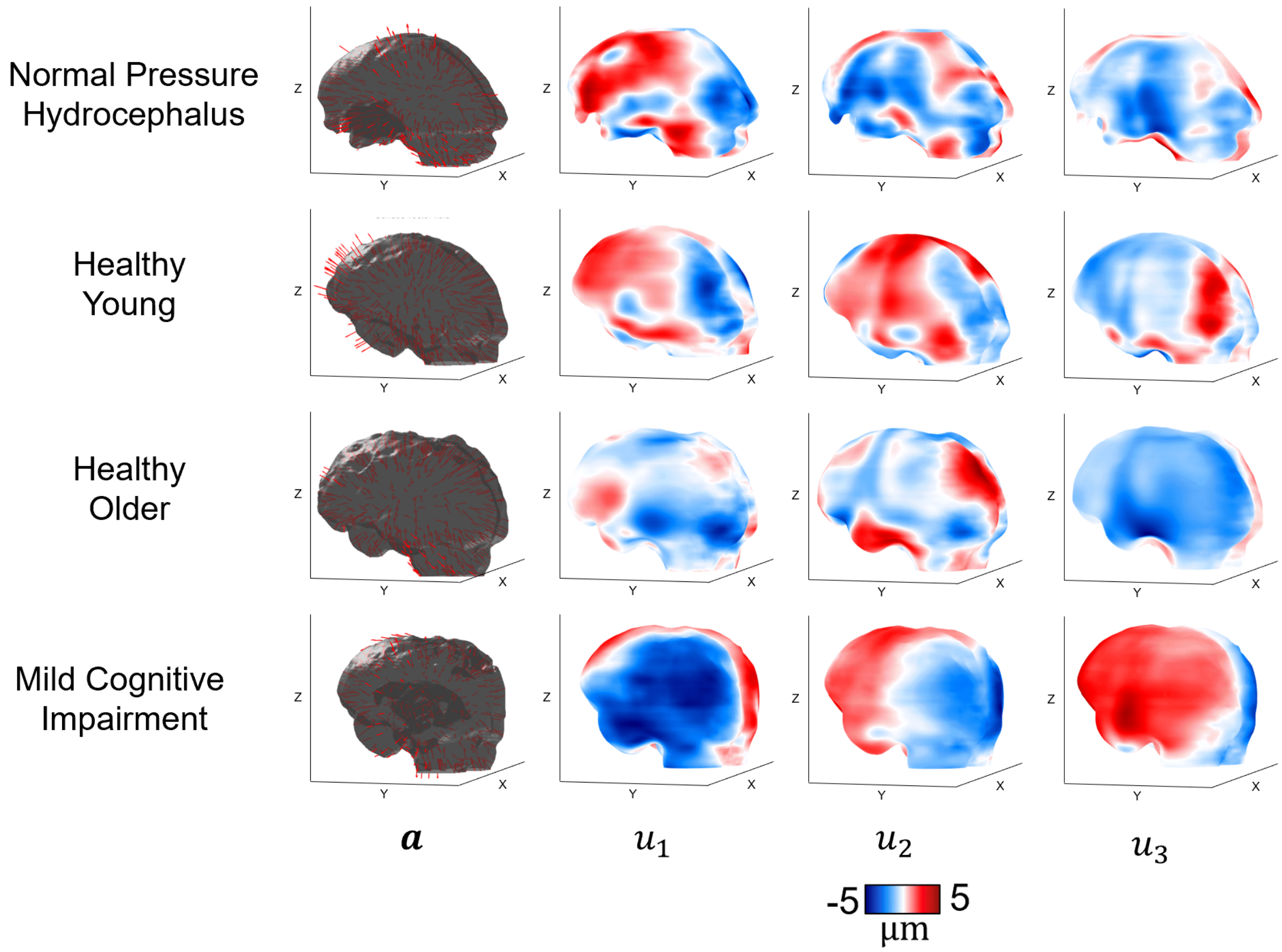

Supplementary Fig. 11 3D cortical surface contours segmented from T1 maps, with arrows indicating the chosen normal prestress directions of four typical volunteers. Adjacent panels show the cortex's shear wave fields measured by MRE along the three encoding directions. This figure corresponds to Fig. 7.

Supplementary Note 1: Theoretical foundations of TWE-guided analytical prestress inversion

Prestress wave propagation model under uniaxial compression

The Mooney–Rivlin model can accurately characterize the incompressible, hyperelastic behavior of brain tissue under large deformations[1]. Under uniaxial stress, hyperelastic materials exhibit acoustoelastic effects[2,3], where the superimposed small-amplitude shear wave experiences changes in wave speed, displaying features similar to transverse isotropy. Further studies have demonstrated a specific analytical relationship between wave polarization and wave speed in small-amplitude waves propagating through incompressible Mooney–Rivlin materials under uniaxial stress[4].

The strain energy function of the Mooney–Rivlin model is defined as:

$$W = C_{10}(I_1 - 3) + C_{01}(I_2 - 3) \tag{S1}$$

where C_{10} and C_{01} are material-specific hyperelastic constants, I_1 and I_2 are the first and second invariants of the right Cauchy–Green deformation tensor, respectively. When uniaxial stress is applied along a direction specified by the unit vector $\boldsymbol{a}$, the material deforms in that direction with a stretch ratio λ. A material point P, initially located at position $\mathbf{X}$, moves to a new position $\mathbf{x}$ in the deformed configuration, given by $\mathbf{x} = \mathbf{FX}$, where $\otimes$ means the tensor (outer) product and $\mathbf{F}$ is the deformation gradient:

$$\mathbf{F} = \lambda\mathbf{a}\otimes\mathbf{a} + \frac{1}{\sqrt{\lambda}}(\mathbf{I} - \mathbf{a}\otimes\mathbf{a}) \tag{S2}$$

To analyze wave propagation, we introduce a small-amplitude, monochromatic shear wave traveling in the deformed material along the direction $\mathbf{n}$. This wavefield supports two orthogonally polarized shear waves, denoted shear-horizontal (SH) wave u_{SH} and shear-vertical (SV) u_{SV}. Let θ denote the angle between the propagation direction $\boldsymbol{n}$ and the principal stress direction $\boldsymbol{a}$, and ρ the material density. Then, the wave polarizations $\mathbf{m}_{SH}$ and $\mathbf{m}_{SV}$, along with the corresponding wave speeds v_{SH} and v_{SV}, can be expressed analytically[4] in Eq.(1). The wave characteristics are directly governed by three parameters in Eq.(2) which encapsulate both the intrinsic material properties and the pre-deformation state. These parameters determine the anisotropic wave behavior under prestressed conditions.

Traveling wave expansion model for reverberant prestress wavefields

When a time-harmonic excitation with angular frequency ω is introduced into biological tissue, the resulting wavefield often exhibits a reverberant pattern due to the complexity of internal structures and boundary conditions. Assuming the locally deformed tissue supports L_{SH} SH waves and L_{SV} SV waves, the reverberant wavefield at a spatial point $\mathbf{r}$ can be expressed using a traveling wave expiation (TWE) model[5,6] as:

$$\begin{aligned}\mathbf{U}(\mathbf{r}) &= \sum_{p=1}^{L_{SH}} A_{SH}^{p} \exp\left(ik_{SH}^{p}\mathbf{n}_{SH}^{p} \cdot \mathbf{r}\right) \cdot \mathbf{m}_{SH}^{p} + \sum_{q=1}^{L_{SV}} A_{SV}^{q} \exp\left(ik_{SV}^{q}\mathbf{n}_{SV}^{q} \cdot \mathbf{r}\right) \cdot \mathbf{m}_{SV}^{q} \\ &= \sum_{p=1}^{L_{SH}} u_{SH}^{p} \cdot \mathbf{m}_{SH}^{p} + \sum_{q=1}^{L_{SV}} u_{SV}^{q} \cdot \mathbf{m}_{SV}^{q} \\ &= \mathbf{U}_{SH} + \mathbf{U}_{SV}\end{aligned} \tag{S3}$$

where the superscripts p and q denote the indices of individual SH and SV wave components, respectively. A_{SH} and A_{SV} represent the complex amplitudes of each SH and SV wave, k_{SH} and k_{SV} are their corresponding wave vectors, and $\mathbf{U}_{SH}$ and $\mathbf{U}_{SV}$ denote their polarization directions.

Analytical relationship construction between wavefields and prestress parameters

Building on the methodological design framework established in our previous work[5,6], we developed a set of wavefield processing operators with clear physical meaning to analyze reverberant wavefields under prestress. In reverberant fields, the individual wave components are difficult to separate and do not require explicit decomposition. Therefore, the operators were designed to act collectively on all SH and SV waves across different propagation directions.

To ensure consistency with the physical behavior of wavefields under prestress (Proved in Supplementary Note 2), we formulated a unified operator system that enables polarization modulation between SV and SH waves, selective filtering to suppress SH components, and angular-domain weighted modulation to reconstruct the wavefield based on the angle-dependent parameter θ:

$$
\left\{
\begin{aligned}
(\nabla\cdot\mathbf{a})^2\mathbf{U} &= -\sum\nolimits_{p=1}^{L_{SH}}\cos^2\theta_{SH}^{p}\cdot {k_{SH}^{p}}^{2}\cdot u_{SH}^{p}\cdot\mathbf{m}_{SH}^{p} \\
&\quad -\sum\nolimits_{q=1}^{L_{SV}}\cos^2\theta_{SV}^{q}\cdot {k_{SV}^{q}}^{2}\cdot u_{SV}^{q}\cdot\mathbf{m}_{SV}^{q} \\
|(\nabla\times\mathbf{a})|^2\mathbf{U} &= -\sum\nolimits_{p=1}^{L_{SH}}\sin^2\theta_{SH}^{p}\cdot {k_{SH}^{p}}^{2}\cdot u_{SH}^{p}\cdot\mathbf{m}_{SH}^{p} \\
&\quad -\sum\nolimits_{q=1}^{L_{SV}}\sin^2\theta_{SV}^{q}\cdot {k_{SV}^{q}}^{2}\cdot u_{SV}^{q}\cdot\mathbf{m}_{SV}^{q} \\
\nabla\times\mathbf{U} &= \sum\nolimits_{p=1}^{L_{SH}} ik_{SH}^{p}\cdot u_{SH}^{p}\cdot\mathbf{m}_{SV}^{p} - \sum\nolimits_{q=1}^{L_{SV}} ik_{SH}^{q}\cdot u_{SH}^{q}\cdot\mathbf{m}_{SH}^{q} \\
(\mathbf{a}\cdot\mathbf{U})\cdot(\nabla\times\mathbf{a}) &= -\sum\nolimits_{q=1}^{L_{SV}}\sin^2\theta_{SV}^{q}\cdot ik_{SH}^{p}\cdot u_{SV}^{q}\cdot\mathbf{m}_{SV}^{q}
\end{aligned}
\right.
\tag{S4}
$$

where Nabla operator is defined as $\nabla = \left[\frac{\partial}{\partial x_1},\frac{\partial}{\partial x_2},\frac{\partial}{\partial x_3}\right]^{\mathrm{T}}$.

Given the wave velocity relation $c = \omega/k$, Eq.S3 and Eq.S5 can be combined to derive the wavefield characteristics that reflect the influence of both material properties and prestress:

$$
\left\{
\begin{aligned}
\rho\omega^2 &= {k_{SH}^{p}}^{2}\left(\mu_A\cos^2\theta_{SH}^{p} + \mu_B\sin^2\theta_{SH}^{p}\right) \\
\rho\omega^2 &= {k_{SV}^{q}}^{2}\left(\mu_A\cos^2\theta_{SV}^{q} + \mu_C\sin^2\theta_{SV}^{q}\right) \\
\mathbf{U} &= \sum\nolimits_{p=1}^{L_s} u_{SH}^{p}\cdot\left(\mathbf{n}_{SH}^{p}\times\mathbf{a}\right) + \sum\nolimits_{q=1}^{L_f} u_{SV}^{q}\cdot\left(\mathbf{n}_{SV}^{q}\times\left(\mathbf{n}_{SV}^{q}\times\mathbf{a}\right)\right)
\end{aligned}
\right.
\tag{S5}
$$

By integrating these operators with the derived wavefield properties, we establish an analytical relationship that accurately links the reverberant wavefield to the mechanical parameters of the tissue, the direction of uniaxial stress, and the associated stretch ratio:

$$
\left\{
\begin{aligned}
-\rho\omega^2\mathbf{U} &= \mu_A\cdot\boldsymbol{L}_1 + \mu_B\cdot\boldsymbol{L}_2 + \mu_C.(\boldsymbol{L}_3 - \boldsymbol{L}_2) \\
\mathbf{L}_1 &= (\nabla\cdot\mathbf{a})^2\mathbf{U} \\
\mathbf{L}_2 &= -\left(\mathbf{a}\cdot(\nabla\times\mathbf{U})\right)\cdot(\nabla\times\mathbf{a}) \\
\mathbf{L}_3 &= |(\nabla\times\mathbf{a})|^2\mathbf{U}
\end{aligned}
\right.
\tag{S6}
$$

This framework provides a robust and physically interpretable basis for quantifying pre-stress in complex biological environments.

Analytical inversion of prestress parameters under uniaxial compression

When the direction of uniaxial compression $\boldsymbol{a}$ is known, estimating the parameters μ_A, μ_B and μ_C from the wavefield becomes a matter of solving a linear system of three equations with three unknowns. These equations can be expressed in the following form:

$$\begin{bmatrix}\mu_A\\ \mu_B\\ \mu_C\end{bmatrix} = -\rho\omega^2([\mathbf{L}_1 \quad \mathbf{L}_2 \quad (\mathbf{L}_3-\mathbf{L}_2)])^{-1}\mathbf{U} \tag{S7}$$

We then establish the relationship between μ_A, μ_B and μ_C, and the underlying prestress and material properties. For Mooney–Rivlin materials, the Cauchy stress tensor[4] under uniaxial compression is given by $\boldsymbol{\sigma} = -p\mathbf{1} + 2C_{10}\mathbf{B} - 2C_{01}\mathbf{B}^{-1}$, where p is the Lagrange multiplier enforcing incompressibility, $\mathbf{1}$ is the identity matrix, and $\mathbf{B} = \mathbf{F}\mathbf{F}^T$is the Cauchy–Green deformation tensor. Under uniaxial pre-compression, the difference between the two principal stresses corresponds to the axial stress $\sigma_p = \sigma$, which can be directly obtained. By incorporating this relationship into Eq.S4, and using the expression for the shear modulus in Mooney–Rivlin materials, $\mu = 2C_{01} + 2C_{10}$, we derive a closed-form relation linking the material parameters, deformation characteristics, and prestress to the wavefield parameters μ_A, μ_B and μ_C:

$$\begin{cases} \sigma = \mu_A - \mu_C \\ \lambda = \left(\dfrac{\mu_A}{\mu_C}\right)^{1/3} \\ \mu = \dfrac{\lambda^2\mu_C + \lambda\mu_B + \mu_A}{\lambda^2+\lambda+1} \end{cases} \tag{S8}$$

Together with Eq.S7 and Eq.S8, this formulation provides a complete framework for estimating prestress from wavefield measurements in soft biological tissues.

Supplementary Note 2: Proof of fundamental operator properties in prestress wavefields

The reverberant wavefield of Mooney–Rivlin materials under uniaxial stress is defined based on the Traveling Wave Expansion (TWE) model. Below are proofs of the fundamental operator properties of this wavefield based on Eq.S3 and Eq.S5.

Lemma 1.

$$(\boldsymbol{\nabla}\cdot\mathbf{a})^2\mathbf{U} = -\sum_{p=1}^{L_{SH}} \cos^2\theta_{SH}^p \cdot {k_{SH}^p}^2 \cdot u_{SH}^p \cdot \mathbf{m}_{SH}^p - \sum_{q=1}^{L_{SV}} \cos^2\theta_{SV}^q \cdot {k_{SV}^q}^2 \cdot u_{SV}^q \cdot \mathbf{m}_{SV}^q \quad \text{(S9)}$$

where, θ_{SH}^p is the angle between $\mathbf{a}$ and $\mathbf{n}_{SH}^p$ and θ_{SV}^q is the angle between $\mathbf{a}$ and $\mathbf{n}_{SV}^q$.

Proof.

Since,

$$\begin{aligned}(\boldsymbol{\nabla}\cdot\mathbf{a})^2 = a_1^2\frac{\partial^2}{\partial x_1^2} + a_2^2\frac{\partial^2}{\partial x_2^2} + a_3^2\frac{\partial^2}{\partial x_3^2} + 2a_1a_2\frac{\partial^2}{\partial x_1\partial x_2} + 2a_2a_3\frac{\partial^2}{\partial x_2\partial x_3} \\ + 2a_1a_3\frac{\partial^2}{\partial x_1\partial x_3}\end{aligned} \quad \text{(S10)}$$

therefore,

$$\begin{aligned}(\boldsymbol{\nabla}\cdot\mathbf{a})^2u_{SH}^p &= \left(ik_{SH}^p\right)^2\cdot A_p\cdot e^{ik_{SH}^p\boldsymbol{n}_{SH}^p\cdot\boldsymbol{r}}\cdot(a_1^2{n_{SH1}^p}^2 + a_2^2{n_{SH2}^p}^2 + a_3^2{n_{SH3}^p}^2 \\ &\quad + 2a_1a_2n_{SH1}^pn_{SH2}^p + 2a_2a_3n_{SH2}^pn_{SH3}^p + 2a_1a_3n_{SH1}^pn_{SH3}^p) \\ &= \left(ik_{SH}^p\right)^2\cdot A_p\cdot e^{ik_{SH}^p\boldsymbol{n}_{SH}^p\cdot\boldsymbol{r}}\cdot\left(a_1n_{SH1}^p + a_2n_{SH2}^p + a_3n_{SH3}^p\right)^2 \\ &= -\cos^2\theta_{SH}^p\cdot{k_{SH}^p}^2\cdot u_{SH}^p\end{aligned} \quad \text{(S11)}$$

Similarly,

$$(\boldsymbol{\nabla}\cdot\mathbf{a})^2u_{SV}^q = -\cos^2\theta_{SV}^q\cdot{k_{SV}^q}^2\cdot u_{SV}^q \quad \text{(S12)}$$

Then,

$$(\boldsymbol{\nabla}\cdot\mathbf{a})^2\mathbf{U} = \sum_{p=1}^{L_{SH}}(\boldsymbol{\nabla}\cdot\mathbf{a})^2u_{SH}^p\cdot\boldsymbol{m}_{SH}^p + \sum_{q=1}^{L_{SV}}(\boldsymbol{\nabla}\cdot\mathbf{a})^2u_{SV}^q\cdot\mathbf{m}_{SV}^q \quad \text{(S13)}$$

$$= -\sum_{p=1}^{L_{SH}} \cos^2 \theta_{SH}^p \cdot {k_{SH}^p}^2 \cdot u_{SH}^p \cdot \mathbf{m}_{SH}^p$$
$$- \sum_{q=1}^{L_{SV}} \cos^2 \theta_{SV}^q \cdot {k_{SV}^q}^2 \cdot u_{SV}^q \cdot \mathbf{m}_{SV}^q$$

Lemma 2.

$$|(\nabla \times \mathbf{a})|^2 \mathbf{U} = -\sum_{p=1}^{L_{SH}} \sin^2 \theta_{SH}^p \cdot {k_{SH}^p}^2 \cdot u_{SH}^p \cdot \mathbf{m}_{SH}^p - \sum_{q=1}^{L_{SV}} \sin^2 \theta_{SV}^q \cdot {k_{SV}^q}^2 \cdot u_{SV}^q \cdot \mathbf{m}_{SV}^q \quad \text{(S14)}$$

where, θ_{SH}^p is the angle between $\mathbf{a}$ and $\mathbf{n}_{SH}^p$ and θ_{SV}^q is the angle between $\mathbf{a}$ and $\mathbf{n}_{SV}^q$.

Proof.

Since,

$$|(\nabla \times \mathbf{a})|^2 = (a_2^2 + a_3^2)\frac{\partial^2}{\partial x_1^2} + (a_1^2 + a_3^2)\frac{\partial^2}{\partial x_2^2} + (a_1^2 + a_2^2)\frac{\partial^2}{\partial x_3^2} - 2a_1 a_2 \frac{\partial^2}{\partial x_1 \partial x_2} - 2a_2 a_3 \frac{\partial^2}{\partial x_2 \partial x_3} - 2a_1 a_3 \frac{\partial^2}{\partial x_1 \partial x_3} \quad \text{(S15)}$$

therefore,

$$\begin{aligned} |(\nabla \times \mathbf{a})|^2 u_{SH}^p &= (ik_{SH}^p)^2 \cdot A_p \cdot \exp\left(ik_{SH}^p \mathbf{n}_{SH}^p \cdot \mathbf{r}\right) \cdot ((a_2^2 + a_3^2){n_{SH1}^p}^2 \\ &+ (a_1^2 + a_3^2){n_{SH2}^p}^2 + (a_1^2 + a_2^2){n_{SH3}^p}^2 - 2a_1 a_2 n_{SH1}^p n_{SH2}^p \\ &- 2a_2 a_3 n_{SH2}^p n_{SH3}^p - 2a_1 a_3 n_{SH1}^p n_{SH3}^p) \\ &= (ik_{SH}^p)^2 \cdot A_p \cdot \exp\left(ik_{SH}^p \mathbf{n}_{SH}^p \cdot \mathbf{r}\right) \\ &\cdot \left(\left(-a_2 n_{SH1}^p + a_1 n_{SH2}^p\right)^2 + \left(a_3 n_{SH1}^p - a_1 n_{SH3}^p\right)^2 \right. \\ &\left. + \left(-a_3 n_{SH2}^p + a_2 n_{SH3}^p\right)^2\right) = -\sin^2 \theta_{SH}^p \cdot {k_{SH}^p}^2 \cdot u_{SH}^p \end{aligned} \quad \text{(S16)}$$

Similarly,

$$|(\nabla \times \mathbf{a})|^2 u_{SV}^q = -\sin \theta_{SV}^q \cdot {k_{SV}^q}^2 \cdot u_{SV}^q \quad \text{(S17)}$$

Then,

$$|(\nabla \times \mathbf{a})|^2\mathbf{U} = \sum_{p=1}^{L_{SH}} |(\nabla \times \mathbf{a})|^2 u_{SH}^p \cdot \mathbf{m}_{SH}^p + \sum_{q=1}^{L_{SV}} |(\nabla \times \mathbf{a})|^2 u_{SV}^q \cdot \mathbf{m}_{SV}^q$$
$$= -\sum_{p=1}^{L_{SH}} \sin^2\theta_{SH}^p \cdot {k_{SH}^p}^2 \cdot u_{SH}^p \cdot \mathbf{m}_{SH}^p \tag{S18}$$
$$-\sum_{q=1}^{L_{SV}} \sin^2\theta_{SV}^q \cdot {k_{SV}^q}^2 \cdot u_{SV}^q \cdot \mathbf{m}_{SV}^q$$

Lemma 3.

$$\nabla \times \mathbf{U} = \sum_{p=1}^{L_{SH}} ik_{SH}^p \cdot u_{SH}^p \cdot \mathbf{n}_{SH}^p \times \left(\mathbf{n}_{SH}^p \times \mathbf{a}\right) - \sum_{q=1}^{L_{SV}} ik_{SV}^q \cdot u_{SV}^q \cdot \left(\mathbf{n}_{SV}^q \times \mathbf{a}\right) \tag{S19}$$

Proof.

$$\nabla \times \mathbf{U} = \nabla \times \left(\sum_{p=1}^{L_{SH}} u_{SH}^p \cdot \mathbf{m}_{SH}^p + \sum_{q=1}^{L_{SV}} u_{SV}^q \cdot \mathbf{m}_{SV}^q\right)$$
$$= \nabla \times \left(\sum_{p=1}^{L_{SH}} u_{SH}^p\left(\mathbf{n}_{SH}^p \times \mathbf{a}\right) + \sum_{q=1}^{L_{SV}} u_{SV}^q \left(\mathbf{n}_{SV}^q \times \left(\mathbf{n}_{SV}^q \times \mathbf{a}\right)\right)\right)$$
$$= \sum_{p=1}^{L_{SH}} ik_{SH}^p \cdot A_p \cdot e^{ik_{SH}^p \mathbf{n}_{SH}^p \cdot \mathbf{r}} \cdot \mathbf{n}_{SH}^p \times \left(\mathbf{n}_{SH}^p \times \mathbf{a}\right)$$
$$+ \sum_{q=1}^{L_{SV}} ik_{SV}^q \cdot A_q \cdot e^{ik_{SV}^q \mathbf{n}_{SV}^q \cdot \mathbf{r}} \cdot \mathbf{n}_{SV}^q \times \left(\mathbf{n}_{SV}^q \times \left(\mathbf{n}_{SV}^q \times \mathbf{a}\right)\right)$$
$$= \sum_{p=1}^{L_{SH}} ik_{SH}^p \cdot A_p \cdot e^{ik_{SH}^p \mathbf{n}_{SH}^p \cdot \mathbf{r}} \cdot \mathbf{n}_{SH}^p \times \left(\mathbf{n}_{SH}^p \times \mathbf{a}\right) \tag{S20}$$
$$+ \sum_{q=1}^{L_{SV}} ik_{SV}^q \cdot A_q \cdot e^{ik_{SV}^q \mathbf{n}_{SV}^q \cdot \mathbf{r}} \cdot \mathbf{n}_{SV}^q \times \left(\mathbf{n}_{SV}^q\left(\mathbf{n}_{SV}^q \cdot \mathbf{a}\right) - \mathbf{a}\left(\mathbf{n}_{SV}^q \cdot \mathbf{n}_{SV}^q\right)\right)$$
$$= \sum_{p=1}^{L_{SH}} ik_{SH}^p \cdot A_p \cdot e^{ik_{SH}^p \mathbf{n}_{SH}^p \cdot \mathbf{r}} \cdot \mathbf{n}_{SH}^p \times \left(\mathbf{n}_{SH}^p \times \mathbf{a}\right)$$
$$- \sum_{q=1}^{L_{SV}} ik_{SV}^q \cdot A_q \cdot e^{ik_{SV}^q \mathbf{n}_{SV}^q \cdot \mathbf{r}} \cdot \left(\mathbf{n}_{SV}^q \times \mathbf{a}\right)$$
$$= \sum_{p=1}^{L_{SH}} ik_{SH}^p \cdot u_{SH}^p \cdot \mathbf{n}_{SH}^p \times \left(\mathbf{n}_{SH}^p \times \mathbf{a}\right) - \sum_{q=1}^{L_{SV}} ik_{SV}^q \cdot u_{SV}^q \cdot \left(\mathbf{n}_{SV}^q \times \mathbf{a}\right)$$

Lemma 4.

$$\mathbf{a} \cdot \mathbf{U} = -\sum_{q=1}^{L_{SV}} u_{SV}^q \cdot \sin^2\theta_{SV}^q \tag{S21}$$

Proof.

$$
\begin{aligned}
\mathbf{a}\cdot\mathbf{U} &= \mathbf{a}\cdot\left(\sum_{p=1}^{L_{SH}} u_{SH}^{p}\left(\mathbf{n}_{SH}^{p}\times\mathbf{a}\right) + \sum_{q=1}^{L_{SV}} u_{SV}^{q}\left(\mathbf{n}_{SV}^{q}\times\left(\mathbf{n}_{SV}^{q}\times\mathbf{a}\right)\right)\right)\\
&= \mathbf{a}\cdot\sum_{q=1}^{L_{SV}} u_{SV}^{q}\cdot\left(\mathbf{n}_{SV}^{q}\left(\mathbf{n}_{SV}^{q}\cdot\mathbf{a}\right) - \mathbf{a}\left(\mathbf{n}_{SV}^{q}\cdot\mathbf{n}_{SV}^{q}\right)\right)\\
&= \sum_{q=1}^{L_{SV}} u_{SV}^{q}\cdot\left(\left(\mathbf{n}_{SV}^{q}\cdot\mathbf{a}\right)^{2} - 1\right)\\
&= -\sum_{q=1}^{L_{SV}} u_{SV}^{q}\cdot\sin^{2}\theta_{SV}^{q}
\end{aligned}
\tag{S22}
$$

Supplementary Note 3: Simulation of wavefield under prestress

The analytical model of wave propagation for prestressed Mooney–Rivlin materials (Eq. 1) was validated using FE simulations (COMSOL v6.2). A homogeneous block (100 mm × 30 mm × 100 mm) was assigned a Mooney–Rivlin constitutive law with constants $C_{10} = C_{01} = 500\mathrm{Pa}$. Quasi-static prestrain was imposed by applying a uniaxial preload in the y–z plane, followed by a small-amplitude shear excitation on the left face, polarized to separate SH and SV components (Supplementary Fig. 1). Wave speeds were extracted from the simulated wavelengths.

Stretch ratio λ varied from 0.6 to 1.4 (step = 0.1), corresponding to up to 40% compression or extension. The prestress direction $\mathbf{a}$ was defined as $[0, -\cos\beta, \sin\beta]$, with β spanning 0 to $\frac{\pi}{2}$ in $\frac{\pi}{18}$ increments, so that the angle θ between $\mathbf{a}$ and the propagation direction $\mathbf{n}$ ranged across 0 to $\frac{\pi}{2}$. A total of 9 × 10 = 90 parameter combinations were tested.

As shown in Supplementary Fig. 1c, simulated SH and SV wave speeds agreed with the analytical predictions over the full $\lambda - \theta$ domain, with an average relative error below 0.83%. These results confirm the validity of the prestress–wave characteristic relationship in Eq. 1 under diverse loading and polarization conditions, thereby underpinning our inversion framework.

Supplementary Note 4: Validation of prestress inversion using simulated wavefield patches

To validate the theoretical performance of our prestress inversion framework, we synthesized 30 Hz reverberant wavefields via the TWE model within compact 3 × 3 × 3 voxel patches (1 mm resolution, Supplementary Fig. 2a), using MATLAB 2022. Each patch comprised a random superposition of SH and SV plane waves. That is, $M = 1/2/3$ distinct propagation directions $\mathbf{n}_{SH}$ and $\mathbf{n}_{SV}$ with complex amplitudes A_{SH} and A_{SV}—generated according to Eq. S3 and Eq. S5. The wavefields subjected to systematic changes in both material properties and prestress orientations $\mathbf{a} = [\cos\theta_2 \sin\theta_1, \sin\theta_2 \sin\theta_1, \cos\theta_1]$. The inversion accuracy was evaluated under two distinct sampling trajectories for prestress orientation and material parameters (Supplementary Fig. 2b).

The first trajectory (yellow) varied the azimuthal angle θ_2 while keeping the polar angle $\theta_1 = \pi/3$ constant. The specific parameter configuration is as follows:

$$\begin{cases} \mathbf{a} = \left[\cos 2\pi\frac{n}{N}\sin\frac{\pi}{3}, \sin 2\pi\frac{n}{N}\sin\frac{\pi}{3}, \cos\frac{\pi}{3}\right] \\ \mu_A = \left(2 + 0.5\cos 8\pi\frac{n}{N}\right)(\text{kPa}) \\ \mu_B = \left(1 + 0.5\sin 6\pi\frac{n}{N}\right)(\text{kPa}) \\ \mu_C = \left(1 + 0.5\sin 6\pi\frac{n}{N} + \pi\right)(\text{kPa}) \end{cases}. \tag{S23}$$

The second trajectory (gray) fixed $\theta_2 = \pi/3$ while varying θ_1. The specific parameter configuration is as follows:

$$\begin{cases} \mathbf{a} = \left[\cos\frac{\pi}{3}\sin 2\pi\frac{n}{N}, \sin\frac{\pi}{3}\sin 2\pi\frac{n}{N}, \cos 2\pi\frac{n}{N}\right] \\ \mu_A = \left(2 + 0.5\sin 8\pi\frac{n}{N}\right)(\text{kPa}) \\ \mu_B = \left(1 + 0.5\cos 6\pi\frac{n}{N}\right)(\text{kPa}) \\ \mu_C = \left(1 + 0.5\cos 6\pi\frac{n}{N} + \pi\right)(\text{kPa}) \end{cases}, \tag{S24}$$

where n represents the current simulation number, and $N = 200$ represents the total number of simulations. In each case, the complex stiffness components μ_A, μ_B, and μ_C were modulated with spatially oscillatory patterns, mimicking heterogeneity typically encountered in biological tissues.

When wavefields contained only one traveling direction ($M = 1$), the inversion results exhibited significant deviations from ground truth, particularly in estimating the transverse anisotropic moduli. This is attributed to the limited directional richness of the reverberant field, which fails to sufficiently excite all mechanical modes needed for robust inversion. In contrast, when two or three different traveling wave directions were present ($M = 2$ and $M = 3$), the relative estimation errors dramatically decreased to 0.45% and 0.27%, respectively (Supplementary Fig. 2c).

Notably, the proposed method achieved this performance using only a single-frequency, small-scale (3×3×3) local patch, indicating strong potential for practical in vivo application where spatial resolution and acquisition time are limited. The localized nature of the approach makes it especially promising for characterizing small or spatially heterogeneous lesions such as brain tumors or fibrotic nodules, where prestress-induced anisotropy is expected to be pronounced and clinically informative.

Supplementary Note 5: Assumption of uniaxial stress for in vivo tissue stress estimation

In this study, stress inversion is formulated under a uniaxial-dominant assumption, where the local stress state is represented by an effective stretch ratio along a primary axis. Although multiaxial stress state may exist, this Note clarifies that the assumption can still provide satisfactory estimation. If brain tissue is described as a nearly incompressible soft solid, the Cauchy stress tensor is decomposed into a hydrostatic component and a deviatoric component. Classical nonlinear acoustoelastic theory demonstrates that shear waves are insensitive to hydrostatic pressure and respond exclusively to the deviatoric part, which governs stress-induced anisotropy[7]. Consequently, isotropic pressure, while potentially large in magnitude, does not contribute to measurable shear-wave speed variation. This property fundamentally limits what components of a multiaxial stress field can be observed.

To quantify the influences of multiaxiality and hydrostatic pressure, we performed numerical simulations in COMSOL using a cubic specimen with dimensions of 50×50×50 mm^3. The phantom was modelled as a Mooney-Rivlin material with material parameters $C_{01} = 400\mathrm{Pa}$ and $C_{10} = 600\mathrm{Pa}$. As illustrated in Supplementary Fig. 3a, roller supports were set on three adjacent surfaces, while independent compressive stresses $(\sigma_x, \sigma_y, \sigma_z)$ were applied to the other three surfaces. Shear vibrations were then exerted on the remaining three surfaces at a frequency of 50 Hz. The wavefield analysis procedures adopted in this study were consistent with those detailed in Fig. 2. For the stress inversion, the z-axis was specified as the principal stress orientation. In the z-axis, the stretch ratio λ was derived from the simulation, while the ground-truth stress σ_{GT} was further obtained using Eq. S8.

- 2D Multiaxial Loading ($\sigma_z > \sigma_x, \sigma_y = 0$): We fixed $\sigma_y = 0$, $\sigma_z = 1500\mathrm{Pa}$ and increased σ_x from 0 to 500 Pa (lateral stress ratio σ_x/σ_z up to 1/3). While the estimation error increases with multiaxiality, it remains below 5% even when the lateral stress is 1/3 of the primary stress (Supplementary Fig. 3b). For complex scenarios, the state can be viewed as a superposition of a hydrostatic part and a uniaxial-dominant deviatoric part, which our method recovers with acceptable accuracy.
- 3D Symmetric Loading ($\sigma_z > \sigma_y = \sigma_x$): We verified the system's insensitivity to hydrostatic pressure by fixing $\sigma_z = 1500\mathrm{Pa}$ and increasing the lateral pressure $\sigma_y = \sigma_x$ from 0 to 500 Pa. In this configuration, the theoretically observable stress

corresponds to the deviatoric difference $\sigma_{diff} = \sigma_z - \sigma_y$. The results show that the relative error between σ_{GT} and σ_{diff} is negligible (<4e-4), confirming that the stress derived from the deformation state is equivalent to the principal stress difference (Eq. S8). Furthermore, the estimated stress σ_{EST} obtained through our wave-based inversion remained highly consistent with σ_{diff}, with a relative error below 1% (Supplementary Fig. 3c). This dual verification shows that the proposed framework effectively filters out hydrostatic pressure bias and specifically resolves the deviatoric stress components that reflect the actual compressive state of the tissue.

- 3D Asymmetric Loading: We performed a parameter sweep where both σ_x and σ_y independently varied from 0 to 500Pa in steps of 50 Pa (fix $\sigma_z = 1500\text{Pa}$). As shown in Supplementary Fig. 3d, our method consistently recovers the primary stress σ_z across all combinations, with a maximum absolute error rate below 8.6% (130 Pa).

Reconstructing a full stress tensor from clinical MRE is often ill-posed. By capturing the dominant deviatoric component, we are able to provide a stable and practical metric that preserves directional accuracy without the numerical instability of over-parameterized models.

Supplementary Note 6: Stress is the primary contributor for differences in wave speed

The proposed stress-estimation framework leverages directional shear-wave speed variations. However, brain tissue—particularly white matter with its organized fiber tracts—exhibits mechanical anisotropy, which could confound stress estimation [8]. To isolate the effect of stress, it is necessary to distinguish and compare the influences of mechanical anisotropy against those of stress-induced changes. Accordingly, we compared profiles of the apparent shear modulus ($G_{app} = \rho c_{app}^2$) obtained from an anisotropic constitutive model[9] with those arising from stress-induced states. For this analysis, we adopted a Nearly Incompressible Transversely Isotropic (NITI) model to evaluate the influence of mechanical anisotropy.

As shown in Fig. S4a,b, with an applied stress of 1.5 kPa, the angular variation of the apparent shear modulus (G_{app}) for both SV and SH waves significantly exceeds the variation attributable to mechanical anisotropy (using representative brain anisotropic parameters: shear anisotropy φ = 0.1, tensile anisotropy ζ = 0.06[9]). Under stresses of 1.5 kPa and 3 kPa, the ratio of maximum to minimum G_{app} for SH/SV waves reaches approximately 1.5/2.1 and 2.1/3.7, respectively. In contrast, for an NITI material without applied stress, this ratio remains relatively low (~1.1/1.03; Fig. S4c). For ICP estimation (e.g., ~500 Pa), we use the global spatial average of stress over the entire cortical surface rather than local pointwise measurements. This large-scale averaging mitigates direction-dependent biases arising from local fiber tracts, ensuring that the estimated ICP reflects systemic pressure rather than localized tissue microstructure. Collectively, these results demonstrate that the observed directional wave-speed differences are dominated by applied stress, with minimal contribution from the intrinsic mechanical anisotropy of brain tissue.

Supplementary Note 7: The effect of visco-hyperelastic material behavior on stress estimation

To evaluate the sensitivity of the proposed stress-inversion framework to viscoelastic material behavior, we performed systematic simulations using a visco-hyperelastic constitutive model. The Mooney–Rivlin hyperelastic model was coupled with a generalized Maxwell viscoelastic model (single branch, relaxation time $\tau = 3$ ms) in COMSOL Multiphysics. Three energy factors ($\alpha =$ 0.18, 0.33, 0.46) were selected to produce damping ratio G' / G'' $=$0.1, 0.2, and 0.3 at 50 Hz, spanning the physiologically reported range for in vivo brain tissue at MRE frequencies10,11.

In a prestressed visco-hyperelastic medium, the effective stiffness is direction-dependent; viscoelastic relaxation acting on these anisotropic stiffnesses may introduce additional directional variations in phase velocity beyond those caused by the acoustoelastic effect alone, potentially biasing the stress estimate. The visco-hyperelastic simulations performed here inherently capture this coupling, as the complex dynamic modulus self-consistently reflects both the prestress-induced anisotropy and the viscoelastic response.

Computation results demonstrate that, despite the potential for viscosity-induced phase-velocity bias, stress estimation accuracy degrades gradually and remains robust across the tested damping range (Supplementary Fig. 5). For the homogeneous shell model, the relative estimation errors were 5.13%, 7.67%, and 17.06% for loss tangent values of 0.1, 0.2, and 0.3, respectively. In the heterogeneous embedded-sphere scenario, the corresponding errors were 9.99%, 15.63%, and 23.19%. In both scenarios, the spatial stress distribution is preserved and the dominant error is a systematic magnitude bias rather than a distortion of the stress pattern. These findings indicate that, within the physiological damping range at 50 Hz, the acoustoelastic effect remains the dominant source of directional phase-velocity anisotropy, and the additional bias introduced by viscoelastic dispersion does not substantially confound the clinical stress patterns reported in this study.

Supplementary Note 8: Robustness of stress estimation to displacement noise

To evaluate the sensitivity of the proposed stress-inversion framework to realistic MRE error sources, we performed systematic noise propagation analyses. Gaussian noise at levels of 2%, 4%, and 6% was incorporated into the simulated displacement wave fields of the pressurized hemispherical shell and the heterogeneous embedded-sphere inflation models. For simulations with noisy wave fields, 3D Gaussian filtering (kernel=3x3x3, variance = 1) was applied prior to inversion.

The results demonstrate that the algorithm maintains high accuracy and stability even under significant noise interference (Supplementary Fig. 6). For the homogeneous shell model, the relative estimation errors were 7.92%, 10.8%, and 15.5% for noise levels of 2%, 4%, and 6%, respectively, compared to a baseline error of 2.09% in the noise-free case. In the more complex heterogeneous scenario, the errors remained stable at 8.17%, 8.74%, and 10.1% (baseline error: 7.84%). A localized error increase observed near 150° in the heterogeneous model may be attributed to edge effects of the Gaussian kernel and data truncation at the boundary. These findings indicate that the stress patterns and clinical trends observed in this study are mainly driven by underlying physiological conditions rather than noise or algorithmic artifacts.

Supplementary Note 9: Stress estimation in spatially heterogeneous media

To evaluate the robustness of the proposed inversion framework in media with spatially varying material properties, we extended the embedded-sphere inflation simulation (Fig. 3) by introducing a linear gradient in the Mooney–Rivlin constants C_{10} and C_{01} along the z-axis. This produces a continuously graded shear modulus field $\mu(z) = 2[C_{10}(z) + C_{01}(z)]$ (with a fixed ratio of $C_{10}: C_{01} = 5: 7$) that varies smoothly across the computational domain, mimicking the possible mechanical heterogeneity encountered in peritumoral brain parenchyma.

Three modulus gradient magnitudes were tested, 3.4, 6.9, and 10 kPa/m, under both positive-gradient (μ increasing along +z) and negative-gradient (μ increasing along −z) configurations, yielding six independent test cases. In all cases, the heterogeneous expansion-induced stress field was first established quasi-statically, followed by 50 Hz shear wave excitation on the deformed configuration. Stress inversion was performed using the same operator-based framework with geometric surface normals as the assumed stress direction.

As shown in Supplementary Fig. 7, the inversion accurately recovers the heterogeneous stress distribution across all tested gradient magnitudes and orientations. Despite local shear modulus variations spanning up to ~30% across the field of view, the mean estimation errors remain between 10.76% and 14.43%, with no systematic degradation as the gradient steepens. Notably, the method correctly tracks the spatial stress profile under both positive and negative gradients, confirming that the quasi-local sliding-kernel inversion naturally adapts to gradual spatial variations in constitutive properties without requiring prior knowledge of the local modulus distribution. These results demonstrate that the proposed framework is applicable to mechanically heterogeneous media.

References

1. Destrade, M., Gilchrist, M. D., Murphy, J. G., Rashid, B. & Saccomandi, G. Extreme softness of brain matter in simple shear. *International Journal of Non-Linear Mechanics* **75**, 54–58 (2015).
2. Caenen, A. *et al.* Analysis of multiple shear wave modes in a nonlinear soft solid: Experiments and finite element simulations with a tilted acoustic radiation force. *Journal of the Mechanical Behavior of Biomedical Materials* **107**, 103754 (2020).
3. Urban, M. W. *et al.* Characterization of transverse isotropy in compressed tissue-mimicking phantoms. *IEEE Trans. Ultrason., Ferroelect., Freq. Contr.* **62**, 1036–1046 (2015).
4. Rouze, N. C., Caenen, A. & Nightingale, K. R. Phase and group velocities for shear wave propagation in an incompressible, hyperelastic material with uniaxial stretch. *Phys. Med. Biol.* **67**, 095015 (2022).
5. Ma, S. *et al.* Measurement of biomechanical properties of transversely isotropic biological tissue using traveling wave expansion. *Medical Image Analysis* **101**, 103457 (2025).
6. Ma, S. *et al.* MR Elastography With Optimization-Based Phase Unwrapping and Traveling Wave Expansion-Based Neural Network (TWENN). *IEEE Trans. Med. Imaging* **42**, 2631–2642 (2023).
7. Destrade, M. & Ogden, R. W. On the third- and fourth-order constants of incompressible isotropic elasticity. *The Journal of the Acoustical Society of America* **128**, 3334–3343 (2010).
8. Tweten, D. J., Okamoto, R. J., Schmidt, J. L., Garbow, J. R. & Bayly, P. V. Estimation of material parameters from slow and fast shear waves in an incompressible, transversely isotropic material. *JOURNAL OF BIOMECHANICS* **48**, 4002–4009 (2015).
9. Ma, S. *et al.* Measurement of biomechanical properties of transversely isotropic biological tissue using traveling wave expansion. *Medical Image Analysis* **101**, 103457 (2025).

10. Hiscox, L. V. *et al.* Magnetic resonance elastography (MRE) of the human brain: technique, findings and clinical applications. *Phys. Med. Biol.* **61**, R401–R437 (2016).

11. Manduca, A. *et al.* MR elastography: Principles, guidelines, and terminology. *MAGNETIC RESONANCE IN MEDICINE* **85**, 2377–2390 (2021).
