# Supplementary material for: Noninvasive 3D mapping of tissue stress using reverberant shear wave
Source: Nat Commun. 2026 Aug 11;17:9626. doi: 10.1038/s41467-026-76509-0 (PMC13554193; doi:10.1038/s41467-026-76509-0)
Supplement: Supplementary file 2 [file 41467_2026_76509_MOESM2_ESM.pdf]

**Description of Additional Supplementary Files**

**Supplementary Movie 1.** Research summary and overview. A 5-minute video presentation covering the research background, methodology, simulation results, phantom experiments, and clinical applications in human participants.
