# Supplementary material for: Noninvasive 3D mapping of tissue stress using reverberant shear wave
Source: Nat Commun. 2026 Aug 11;17:9626. doi: 10.1038/s41467-026-76509-0 (PMC13554193; doi:10.1038/s41467-026-76509-0)
Supplement: Supplementary file 3 [file 41467_2026_76509_MOESM4_ESM.pdf]

nature portfolio

Corresponding author(s): Yuan Feng

Last updated by author(s): Jul 16, 2026

Reporting Summary

Nature Portfolio wishes to improve the reproducibility of the work that we publish. This form provides structure for consistency and transparency in reporting. For further information on Nature Portfolio policies, see our Editorial Policies and the Editorial Policy Checklist.

Statistics

For all statistical analyses, confirm that the following items are present in the figure legend, table legend, main text, or Methods section.

n/a	Confirmed	
☐	☒	The exact sample size (n) for each experimental group/condition, given as a discrete number and unit of measurement
☐	☒	A statement on whether measurements were taken from distinct samples or whether the same sample was measured repeatedly
☐	☒	The statistical test(s) used AND whether they are one- or two-sided<br>*Only common tests should be described solely by name; describe more complex techniques in the Methods section.*
☒	☐	A description of all covariates tested
☒	☐	A description of any assumptions or corrections, such as tests of normality and adjustment for multiple comparisons
☐	☒	A full description of the statistical parameters including central tendency (e.g. means) or other basic estimates (e.g. regression coefficient) AND variation (e.g. standard deviation) or associated estimates of uncertainty (e.g. confidence intervals)
☒	☐	For null hypothesis testing, the test statistic (e.g. F, t, r) with confidence intervals, effect sizes, degrees of freedom and P value noted<br>*Give P values as exact values whenever suitable.*
☒	☐	For Bayesian analysis, information on the choice of priors and Markov chain Monte Carlo settings
☒	☐	For hierarchical and complex designs, identification of the appropriate level for tests and full reporting of outcomes
☒	☐	Estimates of effect sizes (e.g. Cohen's d, Pearson's r), indicating how they were calculated

Our web collection on statistics for biologists contains articles on many of the points above.

Software and code

Policy information about availability of computer code

Data collection	COMSOL Multiphysics (version 6.2) and Abaqus (version 2022) were used for finite element simulations and data generation. In vivo MRI and MRE data were acquired using a 3T clinical MRI scanner (uMR790, United Imaging Healthcare).
Data analysis	Data processing, TWE dataset generation, and stress inversion analyses were performed using MATLAB (R2022a, MathWorks). Brain masks were obtained via skull-stripping using FSL (v6.0.5). Custom processing scripts for the reconstruction workflow are available on Zenodo.

For manuscripts utilizing custom algorithms or software that are central to the research but not yet described in published literature, software must be made available to editors and reviewers. We strongly encourage code deposition in a community repository (e.g. GitHub). See the Nature Portfolio guidelines for submitting code & software for further information.

Data

Policy information about availability of data

All manuscripts must include a data availability statement. This statement should provide the following information, where applicable:

- Accession codes, unique identifiers, or web links for publicly available datasets
- A description of any restrictions on data availability
- For clinical datasets or third party data, please ensure that the statement adheres to our policy

Simulation data and MRE wave field data for a meningioma case have been deposited on zenodo (https://zenodo.org/records/21339517). Source data are provided with this paper.

Research involving human participants, their data, or biological material

Policy information about studies with human participants or human data. See also policy information about sex, gender (identity/presentation), and sexual orientation and race, ethnicity and racism.

Reporting on sex and gender	Sex and gender data are not reported in this study. The primary focus of this research was the physical validation of a physics-informed stress inversion model for soft tissue biomechanics. We did not perform sex- and gender-based analyses as the current sample sizes in pathological sub-groups (n=4) lack sufficient statistical power to yield meaningful conclusions regarding sex differences.
Reporting on race, ethnicity, or other socially relevant groupings	Race and ethnicity data were not collected. The study focuses on universal physical phenomena (acoustoelastic effects in hyperelastic media) and localized anatomical constraints, where race and ethnicity were not considered relevant confounding variables for the primary biomechanical hypotheses tested.
Population characteristics	For the ICP estimation study, participants included healthy young adults (n=18, age 23-28, mean 24), healthy elderly adults (n=20, age 60-80, mean 70), patients with mild cognitive impairment (n=15, age 62-79, mean 69), and patients with normal pressure hydrocephalus (n=15, age 61-86, mean 75).
Recruitment	Participants were recruited prospectively from outpatient clinics at Huashan hospital and Ruijin hospital from Jun. 2022 to Jan. 2025. Diagnoses were confirmed by experienced clinicians based on neuroimaging and clinical signs.
Ethics oversight	This study was approved by the Shanghai Jiao Tong University Ethics Committee (Approval number: E2021119I, E202505281).

Note that full information on the approval of the study protocol must also be provided in the manuscript.

Field-specific reporting

Please select the one below that is the best fit for your research. If you are not sure, read the appropriate sections before making your selection.

☒ Life sciences ☐ Behavioural & social sciences ☐ Ecological, evolutionary & environmental sciences

For a reference copy of the document with all sections, see nature.com/documents/nr-reporting-summary-flat.pdf

Life sciences study design

All studies must disclose on these points even when the disclosure is negative.

Sample size	Sample sizes (n=18 for young, n=20 for elderly, n=15 for MCI, n=15 for NPH, and n=4 for tumor cohorts) were chosen based on the exploratory nature of validating the physics-informed stress inversion model and the availability of patients within the study period.
Data exclusions	No data were excluded from the analyses.
Replication	This is a cross-sectional observational study validating a novel computational framework. Technical replication (repeat MRE scans on the same subject) was not performed. The within-group coefficients of variation (e.g., 13.2% for young, 12.5% for elderly) demonstrate the measurement stability across the cohorts.
Randomization	Randomization is not relevant to this study because it is an observational cohort study comparing pre-existing pathological conditions (e.g., meningioma, pituitary adenoma, MCI, NPH) and age-matched controls, rather than a randomized intervention trial.
Blinding	Investigators were not blinded to group allocation during data collection and analysis, as the diagnoses and anatomical landmarks (e.g., tumor boundaries) required explicit identification on T1-weighted images to define the regions of interest (ROIs) for stress inversion.

Reporting for specific materials, systems and methods

We require information from authors about some types of materials, experimental systems and methods used in many studies. Here, indicate whether each material, system or method listed is relevant to your study. If you are not sure if a list item applies to your research, read the appropriate section before selecting a response.

Materials & experimental systems

n/a	Involved in the study	
☒	☐	Antibodies
☒	☐	Eukaryotic cell lines
☒	☐	Palaeontology and archaeology
☒	☐	Animals and other organisms
☐	☒	Clinical data
☒	☐	Dual use research of concern
☒	☐	Plants

Methods

n/a	Involved in the study	
☒	☐	ChIP-seq
☒	☐	Flow cytometry
☐	☒	MRI-based neuroimaging

Clinical data

Policy information about clinical studies

All manuscripts should comply with the ICMJE guidelines for publication of clinical research and a completed CONSORT checklist must be included with all submissions.

Clinical trial registration	Not applicable. This is an observational study, not an interventional clinical trial.
Study protocol	Not applicable.
Data collection	Data were collected prospectively at Huashan Hospital and Ruijin Hospital, using a 3T MRI scanner (uMR790, United Imaging Healthcare). The data collection period was from Jun. 2022 to Jan. 2025.
Outcomes	The primary pre-defined outcome was the noninvasive quantification of heterogeneous stress distributions (in kPa or Pa) using the proposed traveling wave expansion (TWE) model applied to MRE-derived reverberant shear wave fields.

Plants

Seed stocks	Not applicable.
Novel plant genotypes	Not applicable.
Authentication	Not applicable.

Magnetic resonance imaging

Experimental design

Design type	Elastography imaging.
Design specifications	Not applicable.
Behavioral performance measures	Not applicable.

Acquisition

Imaging type(s)	Elastography (2D SE-EPI MRE)
Field strength	3T
Sequence & imaging parameters	2D SE-EPI MRE: TR/TE = 4000/65 ms, motion-encoding gradient amplitude = 40 mT/m, vibration frequency = 50 Hz, 8 phase-offset images, 3 mm isotropic resolution. T1-weighted imaging
Area of acquisition	Whole brain scan.

Diffusion MRI ☐ Used ☒ Not used

Preprocessing

Preprocessing software	Dual-DC phase unwrapping algorithm implemented in Pytorch (R2022a); FSL (v6.0.5) for skull-stripping.
Normalization	Data were analyzed in native subject space. No spatial normalization to a standard template was performed, as the analysis focused on subject-specific tumor boundaries and cortical geometry.
Normalization template	Not applicable.
Noise and artifact removal	A three-dimensional directional Gaussian filter (σ^2=0.5) for spatial noise reduction, followed by a Butterworth low-pass filter (normalized cutoff frequency = 0.3) for high-frequency disturbance suppression.
Volume censoring	Not applicable.

Statistical modeling & inference

Model type and settings	Not applicable (Standard fMRI GLM models were not used).
Effect(s) tested	Not applicable.

Specify type of analysis: ☐ Whole brain ☒ ROI-based ☐ Both

Anatomical location(s)	ROIs were defined subject-specifically: a 1.5 cm peritumoral shell for meningiomas, a 6 mm superior peritumoral shell for optic chiasm in pituitary adenomas, and a 9 mm inward shell from the cortical surface for ICP estimation. Tumor boundaries were manually delineated.
Statistic type for inference (See Eklund et al. 2016)	Mean stress magnitude within the predefined spatial ROIs (voxel-wise data averaged within ROIs).
Correction	Not applicable.

Models & analysis

n/a	Involved in the study
☒	☐ Functional and/or effective connectivity
☒	☐ Graph analysis
☒	☐ Multivariate modeling or predictive analysis
