# Supplementary material for: Noninvasive 3D mapping of tissue stress using reverberant shear wave
Source: Nat Commun. 2026 Aug 11;17:9626. doi: 10.1038/s41467-026-76509-0 (PMC13554193; doi:10.1038/s41467-026-76509-0)
Supplement: Supplementary file 4 [file 41467_2026_76509_MOESM5_ESM.pdf]

nature portfolio

Peer Review File

Noninvasive 3D mapping of tissue stress using reverberant shear wave

Corresponding Author: Professor Yuan Feng

This file contains all reviewer reports in order by version, followed by all author rebuttals in order by version.

Version 0:

Reviewer comments:

Reviewer #1

(Remarks to the Author)
The growth and pathology of soft tissues alter their internal stress state. Probing this stress is therefore clinically significant and fundamental for understanding the biomechanics underlying many physiological and pathological processes. The manuscript by Ma et al. describes a method that combines acoustoelasticity with magnetic resonance elastography (MRE). The method is validated using simulation data and phantom experiments, followed by preliminary patient studies that illustrate its potential clinical utility. Overall, the paper is interesting and well written, and the proposed method shows promise for clinical translation. Nevertheless, several issues need to be addressed before I can recommend the manuscript for publication.

(1) The working principle of the proposed method relies on acoustoelastic theory, which requires several assumptions regarding the mechanical properties and stress state of the medium. Some of these assumptions should be examined and validated.

(a) The stress in the sample is assumed to be uniaxial, and the stretch ratio is inferred based on this assumption. However, in most scenarios considered in this study, the stress state is likely multiaxial. Could the authors justify the assumption that the uniaxial component is dominant?

(b) The authors claim that the proposed method is independent of constitutive parameters. However, a Mooney–Rivlin model is assumed for the medium, raising uncertainty about whether the method is intrinsically free from constitutive assumptions. Clarification is needed.

(c) Brain tissue is mechanically anisotropic. Since the proposed method relies on directional variations in shear-wave speed to infer stress, the authors should discuss how tissue anisotropy may influence stress estimation and whether corrective modeling or calibration is required.

(d) Material damping is not included in the current formulation. However, the viscoelasticity of brain tissue can substantially affect shear-wave propagation. How does viscoelasticity contribute to the acoustoelastic response, and how might it affect the accuracy or robustness of the proposed method?

(2) What is the spatial resolution of the proposed method? Is the resolution fundamentally limited by the shear wavelength? This question is important because stress concentrations may arise in practical cases. Is the method capable of resolving stress fields with steep spatial gradients?

(3) In the simulation studies, how is the direction of the vector a defined, given that the stress state is multiaxial?

(4) In the inflation test of the hemispherical shell, the circumferential stress should be dominant, yet only the radial stress is evaluated. Why was the circumferential stress not considered?

(5) In Fig. 4d, the MRE wave field exhibits a longer wavelength compared with the simulated field. Can the authors explain this discrepancy? Additionally, how were the constitutive parameters chosen for the simulations? This point is particularly important because the ground-truth stress in the phantom experiments is also obtained through simulations.

(6) For non-invasive estimation of intracranial pressure (ICP), the hydrostatic pressure of the cerebrospinal fluid induces an isotropic stress in brain tissue. For incompressible materials, however, hydrostatic loading does not change shear-wave speed. Could the authors clarify this apparent contradiction?

(7) There is a substantial gap between the phantom and in vivo experiments due to their markedly different mechanical properties. The gelatin phantom is isotropic and elastic, whereas brain tissue is anisotropic and viscoelastic. Is it possible to validate the in vivo measurements using independent techniques? For example, in the ICP experiments, do the authors have access to data obtained using gold-standard measurement methods?

(Remarks on code availability)

Reviewer #2

(Remarks to the Author)
This manuscript presents an interesting framework for estimating tissue stress from reverberant shear wave fields measured with MRE. The analytical approach is clever and supported by simulations and phantom studies. It is also applied to in vivo image data with some interesting results. The topic is timely and of interest to elastography and tissue mechanics. However, the method relies on specific modeling assumptions which are not consistently and clearly presented. Further, the title and text overstate (3D stress) what is estimated (uniaxial stress). Also, the manuscript does not convincingly discuss how stress-induced anisotropy can be delineated from tissue anisotropy or heterogeneity. This is particularly relevant when moving to the in vivo conditions where the fundamental assumptions of isotropy are violated. Clarifying these issues and more fully characterizing methodological robustness would strengthen the work.

Primary Comments

Significance: The authors address an interesting inverse tissue mechanics problem in an interesting and clever new way. That said, the contribution would benefit from deeper context relative to prior work. Material anisotropy and structural heterogeneity would seem capable of generating directional wave signatures similar to those attributed to uniaxial stress. The manuscript does not demonstrate that this characterization is fundamentally more informative than providing an alternative characterization, e.g. wave fields based on material anisotropy.

Conclusions and Claims: The title, “Noninvasive mapping of 3D brain stress…”, is misleading. The method estimates a single axial stress component, not a full 3D stress tensor. This distinction should be reflected in the title and throughout the manuscript to avoid overstating the capability of the approach. Further it is not clear why ‘brain’ is in the title. Brain imaging seems to be more of a potential application than the focus of the paper.

Methodology: The inversion assumes near-incompressibility, isotropy, and uniaxial stress. When applied in vivo, there also seems to be a need to define the primary stress direction. These assumptions fundamentally affect the interpretation of both phantom and in vivo data and should be presented more clearly throughout the paper. It also seems that these assumptions do not hold in some of the situations studied. For example, pressure on a curved tissue induces stress in the lateral direction. The authors should more rigorously define what is meant by a dominant uniaxial stress component. Further, it seems that intrinsic anisotropy, microstructural organization, and boundary effects could produce similar polarization-dependent wave phenomena, and more rigorous discussion or testing of these alternative explanations would be helpful.

Robustness: Simulations and phantoms show internal consistency. However, the method’s sensitivity to realistic MRE error sources is not quantified. How does displacement noise and spatial resolution propagate into stress estimates? Without such analyses, it is difficult to determine whether observed clinical trends reflect physiology or algorithmic artifacts.

(Remarks on code availability)

Reviewer #3

(Remarks to the Author)
This manuscript proposes a physics-based inversion framework that allows direct stress estimation based on reverberant shear wave fields acquired by MRE. It does not rely on wave direction information or characterization. The authors demonstrated this framework could reconstruct the heterogeneous stress distribution accurately on a deformed 3D hemispherical shell model, a simulated sphere inflation embedded in a hyperelastic block, and two rigid hemispherical shells indented gelatin phantoms as well as corresponding COMSOL simulations. In addition, the authors examined this approach on healthy young adults, healthy elderly adults, mild cognitive impairment patients, elderly patients with normal pressure hydrocephalus, patients with intracranial meningioma, and patients with optic chiasm compression by pituitary adenoma. The findings are consistent with current neuropathology and neuroanatomy, and offer new understandings for brain mechanobiology.

Broad Comments

1. The manuscript would benefit from a sharper delineation of how the proposed method (operator-based separation of SV/SH anisotropy, direction-agnostic inversion, and 3D stress recovery from reverberant MRE fields) differs fundamentally and has advantages from prior acoustoelastic approaches (such as directional acoustoelasticity, orthogonal-wave methods, reverberant elastography). The novelty is present, but the text is a bit too broad. Related work worth citing: doi:10.1088/1361-6560/acbbb7.
2. The inversion assumes that brain tissue is modeled as an incompressible isotropic hyperelastic matieral. This assumption should be stated prominently in the methods and included in discussion, particularly given that brain regions such as white matter are often modeled as anisotropic.
3. As anisotropic MRE methods are emerging, it is worth mentioning how stress-induced anisotropy is separatable from intrinsic fiber anisotropy in brain tissue… While I agree that it is distinguishable, readers might appreciate the clarification
4. I believe that from a single frequency reverberant field, the full 3D stress tensor is not identifiable, and only the dominant principal stress component aligned with an assumed direction can be recovered. The manuscript clarify which tensor components are theoretically observable and which are not.
5. The differential operators are the key to the method, so I feel the explanation could be strengthened.. Please provide more rigorous reasoning showing how these operators act differently on SV and SH components, why they yield distinct angular weightings of μA, μB, μC, and how this allows for local inversion. Full derivation is definitely not necessary, but a bit more mathematical rationale would strengthen the paper.
6. The physiological link between surface-normal stress and ICP requires more mechanical justification. The relationship might be reasonable, but the manuscript never explains the mechanical link .. Please also 1) justify the choice of the 1.5 cm peritumoral shell, and 2) define where the quasi-local homogeneity assumption is expected to hold.

Specific Comments
1. In the second paragraph of the introduction, it seems like it would make more sense to start with the idea that there are both invasive and noninvasive categories and then highlight the fact that the noninvasive approaches are limited.
2. There appears to be a discrepancy in labeling the patients in the text vs. Figure 5… The authors referred to the tumor of patient #1 as the frontal lobe lesion, looks like parietal lobe. Similarly, the tumor of patient #2 in Figure 5b is stated as the parietal lobe tumor, but it is a lesion in the frontal lobe. Perhaps the images are swapped?
3. In the caption of Figure 5, the authors describe “mean stress in the top σtop and bottom σbottom hemispheres.” The top and bottom hemisphere definitions are confusing. The authors should either come up with new terminology, or mention the definition “the horizontal plane through the tumor centroid; σtop and σbottom are the average stresses above and below this plane” earlier in the caption. Same in the manuscript, it would be clearer if the authors could explain this numerical value before the first time mentioning it.
4. In the caption of Figure 5, the authors describe “Panels (a)-(d) correspond to four tumors situated at progressively lower levels within the cranial vault,” while Figure 5 (a) - (d) are not necessarily progressively lower as described. It would be better to describe panels (a) - (d) as tumors in different lobes or regions to make it more aligned with the manuscript.
5. In Figure 5 vi, provide the numeric values of each bar for clarity of interpretation, or even better, change to a type of different type of graph that better represents the spatial patterns you want to show.

(Remarks on code availability)
Code repository is empty.

Reviewer #4

(Remarks to the Author)
I co-reviewed this manuscript with one of the reviewers who provided the listed reports. This is part of the Nature Communications initiative to facilitate training in peer review and to provide appropriate recognition for Early Career Researchers who co-review manuscripts.

(Remarks on code availability)

Version 1:

Reviewer comments:

Reviewer #1

(Remarks to the Author)
I appreciate the authors’ responses to my previous comments. While the manuscript has been improved by these revisions, several major limitations remain in the current version.

(1) The assumption of a uniaxial stress state appears both incorrect and unnecessary. First, the stress in brain tissue is inherently three-dimensional. Second, as demonstrated in Fig. S3c, the stress identified by the proposed method corresponds to the deviatoric stress component, regardless of the actual stress state. Given this, it is unnecessary to assume that the underlying stress is uniaxial.
To more clearly convey the concept of stress mapping, I suggest formulating the method under a general three-dimensional stress state and explicitly stating in the Methods section that the proposed approach derives the deviatoric component of the

stress tensor.
A related issue concerns the stretch ratio derived from the proposed method. As indicated by Eq. (S8), a power of 1/3 is adopted, which implicitly depends on the assumed stress state. The identified stretch ratio is therefore not independent of this assumption. This point should be clarified and justified.

(2) The proposed method is inherently model-based, relying on an isotropic, incompressible M–N constitutive model. This represents an important limitation and should be explicitly acknowledged in the manuscript.

(3) In the revised manuscript, the authors claim that the proposed method is effective for heterogeneous media, yet no convincing evidence is provided. In the simulation involving an embedded sphere, the inversion is performed only in the region outside the inclusion, where the medium remains homogeneous. This does not demonstrate applicability within heterogeneous regions. Since tumors are intrinsically heterogeneous, clarification is needed regarding how the method performs under such realistic conditions.

(4) I respectfully disagree with the authors' argument regarding the negligible effect of material damping. It is unclear how viscosity was incorporated into the simulations. Was an imaginary component introduced into the instantaneous shear modulus? If so, the simulation would not properly capture stress-dependent viscoelastic behavior.
To rigorously account for viscosity, a visco-hyperelastic constitutive model should be employed in the simulations. Intuitively, in the high-frequency regime, the phase velocity in soft tissues is often dominated by viscosity rather than stress. Consequently, the predicted speed anisotropy—and thus the proposed stress estimation approach—may depend strongly on wave frequency.
Given that no ground-truth stress data are available for brain tissue experiments, caution must be exercised before concluding that "material damping has negligible impact on stress estimation." This could represent a major limitation of the proposed method.
Have the authors considered performing validation experiments using viscoelastic phantoms to better support the method's applicability to real brain tissues?

(Remarks on code availability)

Reviewer #2

(Remarks to the Author)
This manuscript presents an interesting framework for estimating tissue stress from reverberant shear wave fields measured with MRE. The analytical approach is clever and supported by simulations and phantom studies. It is also applied to in vivo image data with some interesting results. The additional analyses conducte since the original review have addressed some of the technical implementation questions I had.

(Remarks on code availability)

Reviewer #3

(Remarks to the Author)
My comments have been sufficiently addressed by the authors and I have no further comments.

(Remarks on code availability)

Reviewer #4

(Remarks to the Author)
I co-reviewed this manuscript with one of the reviewers who provided the listed reports. This is part of the Nature Communications initiative to facilitate training in peer review and to provide appropriate recognition for Early Career Researchers who co-review manuscripts.

(Remarks on code availability)

Version 2:

Reviewer comments:

Reviewer #1

(Remarks to the Author)
I appreciate the authors' responses to my previous comments. The manuscript has been further improved through these revisions. However, my major concern regarding this work remains unchanged: although the proposed method is interesting,

it may not yet be ready for clinical application because of the unrealistic assumptions adopted in the inversion framework. Consequently, the results should be interpreted and reported with substantial caution.

(1) The assumption of a uniaxial stress state is overly restrictive and not physiologically realistic. The authors argue that the physiological stress state is primarily normal to the boundary and therefore dominated by uniaxial stress. However, this reasoning is not convincing. A normal stress acting around a cavity or tumor boundary will inevitably generate stress components in other directions with comparable magnitudes. Therefore, the actual stress state is expected to be multiaxial rather than uniaxial.

(2) The authors acknowledge the influence of viscoelasticity and attempt to demonstrate through simulations that the associated errors are negligible. In my opinion, this validation is misleading. The simulations employ a stress-relaxation timescale on the order of milliseconds, corresponding to kHz frequencies, which are much higher than the 50 Hz shear waves used in MRE. This choice substantially suppresses viscoelastic effects and therefore minimizes the estimated errors. In practice, however, the relaxation timescale of brain tissues can be on the order of seconds, which could produce significantly larger viscoelastic effects at 50 Hz.

Given the multiple assumptions adopted in this work, including the uniaxial stress state, constitutive modeling choices, and the treatment of viscoelasticity, substantial caution is required when interpreting the reported brain tissue stress measurements. Without independent experimental validation or direct characterization of stress in real tissues, I do not believe the reported stress values can yet be considered reliable.

(Remarks on code availability)
I did not assess the code associated with this manuscript.

Reviewer #2

(Remarks to the Author)

(Remarks on code availability)

Reviewer #3

(Remarks to the Author)

(Remarks on code availability)

Open Access This Peer Review File is licensed under a Creative Commons Attribution 4.0 International License, which

permits use, sharing, adaptation, distribution and reproduction in any medium or format, as long as you give appropriate credit to the original author(s) and the source, provide a link to the Creative Commons license, and indicate if changes were made.
In cases where reviewers are anonymous, credit should be given to 'Anonymous Referee' and the source.
The images or other third party material in this Peer Review File are included in the article's Creative Commons license, unless indicated otherwise in a credit line to the material. If material is not included in the article's Creative Commons license and your intended use is not permitted by statutory regulation or exceeds the permitted use, you will need to obtain permission directly from the copyright holder.
To view a copy of this license, visit https://creativecommons.org/licenses/by/4.0/

Response to Reviewers

We sincerely thank the reviewers for their careful reading of our manuscript and for providing constructive critiques. We have addressed all questions and comments point-by-point below, indicating corresponding revisions in the manuscript. All changes in the revised manuscript have been marked in blue.

Reviewer #1:

The growth and pathology of soft tissues alter their internal stress state. Probing this stress is therefore clinically significant and fundamental for understanding the biomechanics underlying many physiological and pathological processes. The manuscript by Ma et al. describes a method that combines acoustoelasticity with magnetic resonance elastography (MRE). The method is validated using simulation data and phantom experiments, followed by preliminary patient studies that illustrate its potential clinical utility. Overall, the paper is interesting and well written, and the proposed method shows promise for clinical translation. Nevertheless, several issues need to be addressed before I can recommend the manuscript for publication.

Response: We sincerely thank the reviewer for the positive assessment of our work. We appreciate the constructive critiques, which have helped us significantly improve the rigor and clarity of the manuscript. We have addressed each of the concerns accordingly as follows.

(1) The working principle of the proposed method relies on acoustoelastic theory, which requires several assumptions regarding the mechanical properties and stress state of the medium. Some of these assumptions should be examined and validated.

Response: Thank you for pointing this out. In the revised manuscript, we have performed a comprehensive examination of these assumptions to ensure the reliability of our stress estimation. A detailed point-by-point response to each specific assumption and validation follows below. The corresponding changes in the manuscript are marked in blue.

(a) The stress in the sample is assumed to be uniaxial, and the stretch ratio is inferred based on this assumption. However, in most scenarios considered in this study, the stress state is likely multiaxial. Could the authors justify the assumption that the uniaxial component is dominant?

Response: We agree that in vivo stress fields are generally multiaxial. However, our inversion is designed to capture the primary deviatoric stress component, which is the

main contributor of wave speed differences. We have added a new multiaxial simulation study in **Supplementary Note 5** using a triaxial cube model (**Fig. S3**). The results demonstrate that even in a multiaxial stress state, where lateral stresses reach 1/3 of the primary stress, the estimation error for the primary stress remains below 10%. For explanation of the primary uniaxial stress component, we have added in **Supplementary Note 5**:

"**Supplementary Note 5: Assumption of uniaxial stress for *in vivo* tissue stress estimation**

In this study, stress inversion is formulated under a uniaxial-dominant assumption, where the local stress state is represented by an effective stretch ratio along a primary axis. Although multiaxial stress state may exist, this Note clarifies that the assumption can still provide satisfactory estimation. If brain tissue is described as a nearly incompressible soft solid, the Cauchy stress tensor is decomposed into a hydrostatic component and a deviatoric component. Classical nonlinear acoustoelastic theory demonstrates that shear waves are insensitive to hydrostatic pressure and respond exclusively to the deviatoric part, which governs stress-induced anisotropy[1]. Consequently, isotropic pressure, while potentially large in magnitude, does not contribute to measurable shear-wave speed variation. This property fundamentally limits what components of a multiaxial stress field can be observed.

To quantify the influences of multiaxiality and hydrostatic pressure, we performed numerical simulations in COMSOL using a cubic specimen with dimensions of 50×50×50 mm^3. The phantom was modelled as a Mooney-Rivlin material with material parameters $C_{01} = 400\text{Pa}$ and $C_{10} = 600\text{Pa}$. As illustrated in **Fig. S3**a, roller supports were set on three adjacent surfaces, while independent compressive stresses $(\sigma_x, \sigma_y, \sigma_z)$ were applied to the other three surfaces. Shear vibrations were then exerted on the remaining three surfaces at a frequency of 50 Hz. The wavefield analysis procedures adopted in this study were consistent with those detailed in **Fig. 2**. For the stress inversion, the z-axis was specified as the principal stress orientation. In the z-axis, the stretch ratio λ was derived from the simulation, while the ground-truth stress σ_{GT} was further obtained using Eq. S8.

- **2D Multiaxial Loading** $(\sigma_z > \sigma_x, \sigma_y = 0)$: We fixed $\sigma_y = 0$, $\sigma_z = 1500\text{Pa}$ and increased σ_x from 0 to 500 Pa (lateral stress ratio σ_x/σ_z up to 1/3). While the estimation error increases with multiaxiality, it remains below 5% even when the lateral stress is 1/3 of the primary stress (**Fig. S3**b). For complex scenarios, the state can be viewed as a superposition of a hydrostatic part and a uniaxial-dominant deviatoric part, which our method recovers with acceptable accuracy.

- **3D Symmetric Loading** ($\sigma_z > \sigma_y = \sigma_x$): We verified the system's insensitivity to hydrostatic pressure by fixing $\sigma_z = 1500\text{Pa}$ and increasing the lateral pressure $\sigma_y = \sigma_x$ from 0 to 500 Pa. In this configuration, the theoretically observable stress corresponds to the deviatoric difference $\sigma_{diff} = \sigma_z - \sigma_y$. The results show that the relative error between σ_{GT} and σ_{diff} is negligible (<4e-4), confirming that the stress derived from the deformation state is equivalent to the principal stress difference (Eq. S8). Furthermore, the estimated stress σ_{EST} obtained through our wave-based inversion remained highly consistent with σ_{diff}, with a relative error below 1% (**Fig. S3**c). This dual verification shows that the proposed framework effectively filters out hydrostatic pressure bias and specifically resolves the deviatoric stress components that reflect the actual compressive state of the tissue.
- **3D Asymmetric Loading**: We performed a parameter sweep where both σ_x and σ_y independently varied from 0 to 500Pa in steps of 50 Pa (fix $\sigma_z = 1500\text{Pa}$). As shown in **Fig. S3**d, our method consistently recovers the primary stress σ_z across all combinations, with a maximum absolute error rate below 8.6% (130 Pa).

Reconstructing a full stress tensor from clinical MRE is often ill-posed. By capturing the dominant deviatoric component, we are able to provide a stable and practical metric that preserves directional accuracy without the numerical instability of over-parameterized models.

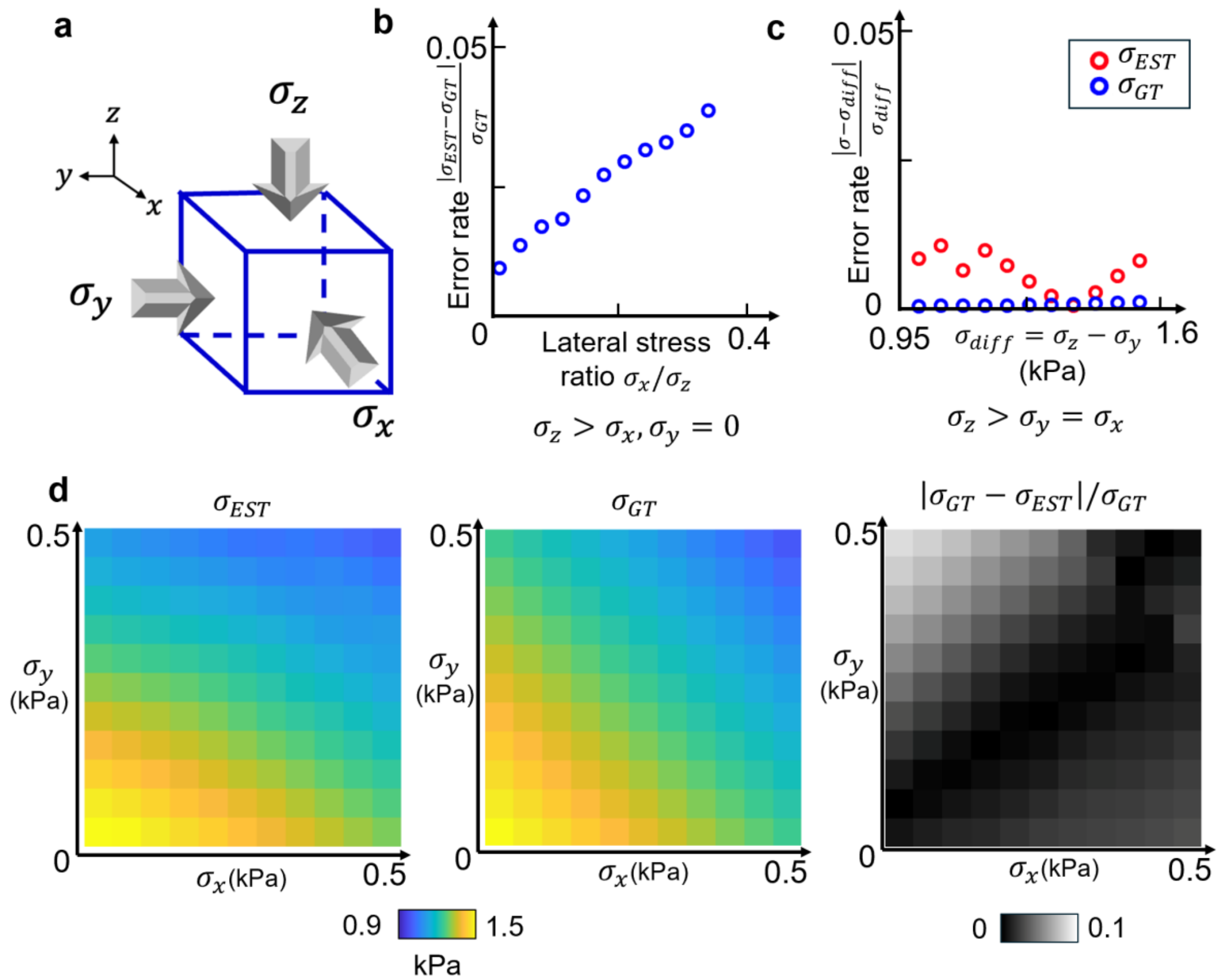

Fig. S3. Validation of the stress estimation under multiaxial stress conditions. **a** A 50×50×50 mm^3 cube with triaxial compressive loads ($\sigma_x, \sigma_y, \sigma_z$). A Mooney-Rivlin material model was used. **b** 2D Multiaxial Case**:** Estimation error for the stress as a function of lateral stress ratio σ_x/σ_z. **c** 3D Symmetric Case: Relative error of the estimated stress (σ_{EST}, red dots) and the stretch-derived stress (σ_{GT}, blue dots) compared to the theoretical deviatoric difference ($\sigma_{diff} = \sigma_{GT} - \sigma_y$). **d** Full 3D Asymmetric Case: Distribution of estimated stress across a comprehensive sweep of independent σ_x and σ_y (0–500 Pa).” (in Supplementary Information).

We have also added the following content in the Discussion section:

“While our method demonstrates promising capabilities, it is subject to certain limitations. The full stress state in tissue is described by a second-order tensor comprising six independent components, making the recovery of all components from a single shear-wave measurement impractical. Under the incompressibility assumption, shear-wave propagation is insensitive to hydrostatic pressure, leaving the full three-dimensional tensor theoretically underdetermined. To render the problem tractable, we reduced the degrees of freedom by adopting an isotropic hyperelastic material model and focusing our analysis on the dominant uniaxial stress component. This simplification aligns with physiological observations where loading is predominantly

normal to tissue boundaries[2,3].Although these assumptions may not fully capture stress complexity in regions subjected to multiaxial loading, we demonstrate that the inversion specifically extracts the deviatoric stress difference while remaining insensitive to hydrostatic bias (**Supplementary Note 5**)." (Lines 564-576).

(b) The authors claim that the proposed method is independent of constitutive parameters. However, a Mooney–Rivlin model is assumed for the medium, raising uncertainty about whether the method is intrinsically free from constitutive assumptions. Clarification is needed.

Response: Thank you for pointing this out. We apologize for the confusion. What we are trying to convey, is that the proposed method does not need a priori knowledge of the material parameters (e.g. C_{10} and C_{01}). However, a constitutive assumption of Mooney–Rivlin model is still needed. To clarify this point, the following revisions were also made at the beginning of the Results section:

"The brain is a soft, structurally complex tissue. While numerous models have been proposed to characterize its biomechanical behavior [4], isotropy and incompressibility remain standard simplifying assumptions[5]. Building on these assumptions, we propose a physics-informed traveling wave expansion (TWE) model. Derived from the hyperelastic Mooney–Rivlin constitutive model [5,6], the TWE framework describes the reverberant wavefield as a linear superposition of shear-vertical (SV) and shear-horizontal (SH) wave components[7,8] propagating along multiple directions (**Fig. 1c**). These components are governed by a stress-dependent constitutive model that links their velocities and polarizations to the underlying local stress state. To obtain this relationship from the measured displacement field (**Fig. 1d**), a structured set of spatial differential operators is applied. These operators are designed to enhance polarization contrast, suppress interfering components, and modulate angular sensitivity across directions (**Fig. 1e**). Operator-based filtering and analysis are performed locally within a sliding three-dimensional kernel (typically 4 × 4 × 4 voxels) (**Fig. 1d, f**), enabling voxel-wise computation of stretch ratio λ and uniaxial stress magnitude σ. Given either known or geometry-inferred principal stress directions, the processed wavefield is analytically inverted using acoustoelastic theory to directly yield local stress estimates. The inversion procedure simultaneously solves for local deformation and constitutive parameters, eliminating the need for prior knowledge of wave-propagation direction or material constants. This design enables robust stress reconstruction in heterogeneous and geometrically complex tissues. Importantly, the method does not rely on material-specific elastic constants or assumed propagation geometry, and it maintains stability in scattering or heterogeneous media. Full methodological details are provided in the Methods section." (Lines 119-142).

(c) Brain tissue is mechanically anisotropic. Since the proposed method relies on directional variations in shear-wave speed to infer stress, the authors should discuss how tissue anisotropy may influence stress estimation and whether corrective modeling or calibration is required.

Response: We thank the reviewer for this insightful comment. We agree that brain tissue, particularly white matter, exhibits mechanical anisotropy[9]. However, our results demonstrate that the anisotropy in wave speed induced by stress is the dominant contributor, exceeding the influence of mechanical anisotropy. To further substantiate this point, we have performed additional computational analyses, which are now presented in a new Supplementary Note:

"**Supplementary Note 6: Stress is the primary contributor for differences in wave speed**

The proposed stress-estimation framework leverages directional shear-wave speed variations. However, brain tissue—particularly white matter with its organized fiber tracts—exhibits mechanical anisotropy, which could confound stress estimation [10]. To isolate the effect of stress, it is necessary to distinguish and compare the influences of mechanical anisotropy against those of stress-induced changes. Accordingly, we compared profiles of the apparent shear modulus ($G_{app} = \rho c_{app}^2$) obtained from an anisotropic constitutive model[11] with those arising from stress-induced states. For this analysis, we adopted a Nearly Incompressible Transversely Isotropic (NITI) model to evaluate the influence of mechanical anisotropy.

As shown in **Fig. S4a,b**, with an applied stress of 1.5 kPa, the angular variation of the apparent shear modulus (G_{app}) for both SV and SH waves significantly exceeds the variation attributable to mechanical anisotropy (using representative brain anisotropic parameters: shear anisotropy $\varphi = 0.1$, tensile anisotropy $\zeta = 0.06$[11]). Under stresses of 1.5 kPa and 3 kPa, the ratio of maximum to minimum G_{app} for SH/SV waves reaches approximately 1.5/2.1 and 2.1/3.7, respectively. In contrast, for an NITI material without applied stress, this ratio remains relatively low (~1.1/1.03; **Fig. S4c**). For ICP estimation (e.g., ~500 Pa), we use the global spatial average of stress over the entire cortical surface rather than local pointwise measurements. This large-scale averaging mitigates direction-dependent biases arising from local fiber tracts, ensuring that the estimated ICP reflects systemic pressure rather than localized tissue microstructure. Collectively, these results demonstrate that the observed directional wave-speed differences are dominated by applied stress, with minimal contribution from the intrinsic mechanical anisotropy of brain tissue.

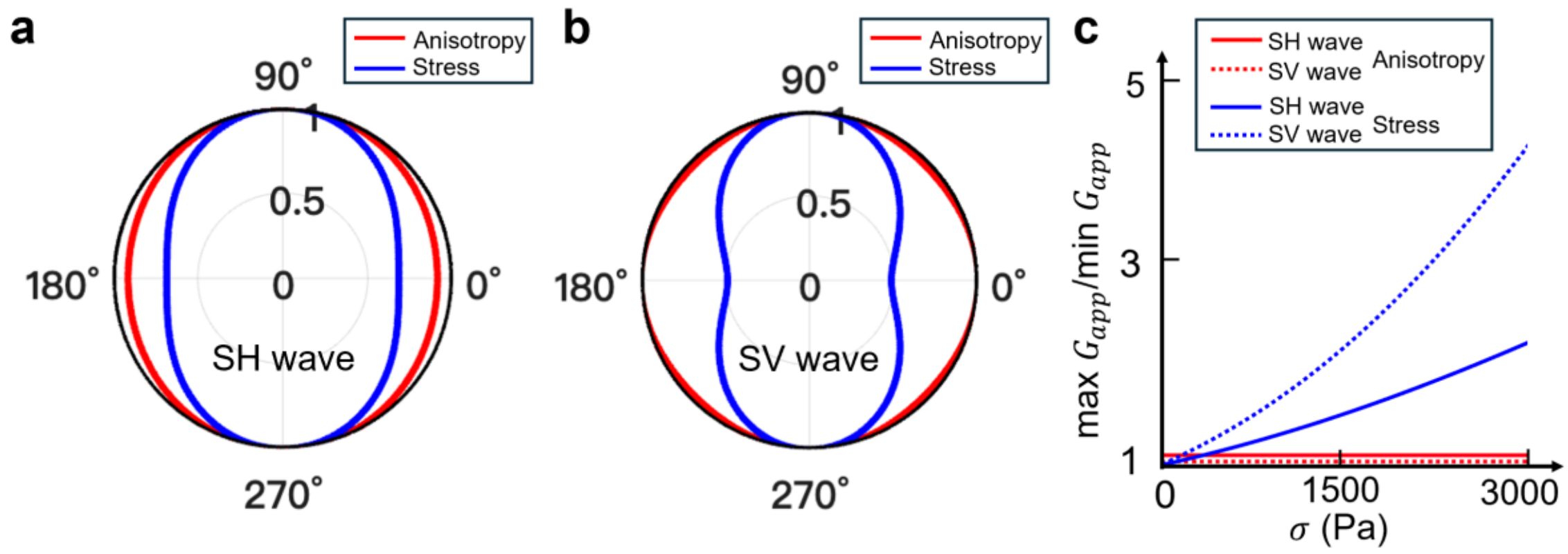

Fig. S4: Comparison of the apparent shear modulus ($G_{app} = \rho c_{app}^2$) due to applied stress versus intrinsic mechanical anisotropy. **a, b** Normalized apparent shear modulus as a function of the angle between wave-propagation direction and the fiber/principal-stress axis, shown for **(a)** SH and **(b)** SV waves. **c** Ratio of maximum to minimum G_{app} ($G_{app,max}$ / $G_{app,min}$) under increasing applied stress, using an anisotropic material without applied stress as the baseline. The pronounced increase in the ratio confirms that applied stress, rather than intrinsic mechanical anisotropy, is the dominant source of wave-speed variation.*"* (in Supplementary Information).

We have also added the following content in the Discussion section:

"Several potential sources of modeling bias are examined in the Supplementary Information. **Supplementary Note 6** compares wave-speed variations induced by stress with those arising from tissue mechanical anisotropy, confirming that acoustoelastic effects dominate directional wave-speed changes." (Lines 587-590).

(d) Material damping is not included in the current formulation. However, the viscoelasticity of brain tissue can substantially affect shear-wave propagation. How does viscoelasticity contribute to the acoustoelastic response, and how might it affect the accuracy or robustness of the proposed method?

Response: We thank the reviewer for this insightful comment. We fully agree that material damping is an important factor. To address this, we have added Supplementary Note 7, where we provide a comprehensive analysis of the influence of viscoelasticity from both theoretical and numerical perspectives:

"**Supplementary Note 7: The effect of tissue damping on stress estimation**

The acoustoelastic effect, which underpins our method, modulates the real part of the wave velocity by altering the incremental elastic modulus under prestress, thereby inducing directional anisotropy in the phase velocity. In contrast, material damping primarily influences the imaginary part of the wave velocity, manifesting as spatial

attenuation of the wave amplitude. Our inversion framework employs differential operators that extract local phase-gradient information from the acquired displacement-phase images. These operators are sensitive solely to phase variations and remain unaffected by amplitude. Consequently, in this study, we leverage phase-velocity anisotropy as the principal carrier of stress information.

To quantitatively evaluate the robustness of the proposed method to tissue damping, we performed sensitivity analyses over a range of damping ratios (defined as the ratio of the imaginary to the real part of the complex shear modulus). We tested damping ratio of 0.1, 0.2, and 0.3, covering the typical physiological range reported for brain tissue[12,13]. As shown in **Fig. S5**, stress reconstruction remains stable across all tested damping levels. The error in estimated stress-tensor components does not increase significantly with damping ratio, confirming that, provided sufficient wave energy and reverberation are maintained, material damping has a negligible impact on the accuracy of stress estimation.

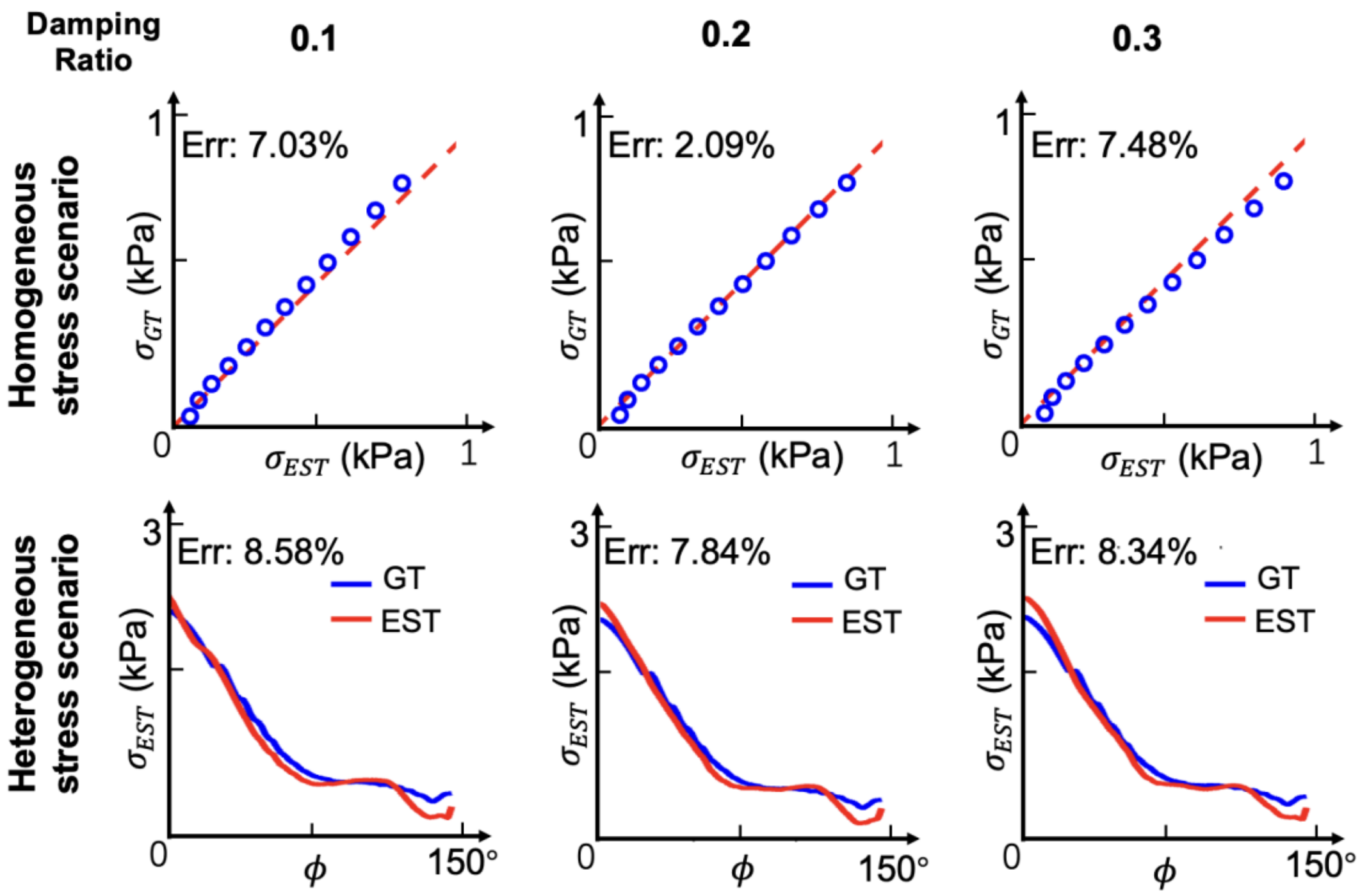

Fig.S5 Stress estimation under varying damping ratios (0.1, 0.2, 0.3). Top row (Fig. 2): Pressurized hemispherical shell simulation. Scatter plot compares ground-truth stress (x-axis) against estimated stress (y-axis). The red dashed line marks the ideal 1:1 correspondence (x = y). Bottom row (Fig. 3): Heterogeneous embedded-sphere inflation simulation. Results verify the influence of damping ratio on stress estimation in a heterogeneous medium. Notably, stress estimation accuracy remains robust despite a wide variation in damping ratio (0.1–0.3) in both simulation settings." (in

Supplementary Information).

We have also added the following content in the Discussion section:

"**Supplementary Note 7** shows that material damping has negligible impact on stress estimation, since the inversion relies primarily on phase-gradient information rather than wave amplitude." (Lines 590-592).

(2) What is the spatial resolution of the proposed method? Is the resolution fundamentally limited by the shear wavelength? This question is important because stress concentrations may arise in practical cases. Is the method capable of resolving stress fields with steep spatial gradients?

Response: Thank you for pointing this out. In the following, we provide point-by-point responses for the three questions:

The achievable spatial resolution of the proposed stress-mapping framework is primarily determined by the spatial resolution of the underlying MRE acquisition. In the current study, the displacement fields were acquired at a resolution of 3 × 3 × 3 mm^3, and accordingly, the reconstructed stress maps reflect heterogeneity at this voxel scale. Achieving finer spatial resolution would require higher-resolution MRE acquisitions, which are currently constrained by signal-to-noise ratio and scan time considerations in clinical settings.

Importantly, the spatial resolution of the proposed method is not fundamentally limited by the shear wavelength, in contrast to many conventional elastography approaches. Our method is based on a local analytical Traveling Wave Expansion (TWE) framework, which extracts stress-induced anisotropy from the directional structure of the reverberant wave field rather than from spatial wavelength fitting. The key requirement for robust inversion is that the local wave field contains sufficient directional richness (i.e., at least two independent wave components) within the analysis window, rather than a specific relationship between wavelength and spatial resolution. This property is explicitly validated in Supplementary **Fig. S2**, where we demonstrate that when only a single propagation direction is present (M = 1), the inversion becomes ill-conditioned, whereas the presence of two or more wave directions (M = 2–3) leads to highly accurate stress and parameter recovery, even within a small 3 × 3 × 3 mm^3 local patch and using a single excitation frequency.

Despite this limitation, our simulations and in vivo results demonstrate that the method is capable of resolving clinically relevant stress heterogeneity and spatial gradients. In particular, the heterogeneous embedded-sphere inflation simulation (Fig. 3) contains pronounced, spatially varying prestress distributions, which are accurately

recovered after down-sampling the displacement field to a clinically realistic 3 mm resolution. The same configuration remains robust when additional displacement noise is introduced, as shown in the Supplementary Information. Furthermore, in vivo results (e.g., **Fig. 5**) reveal heterogeneous stress patterns that are spatially consistent with anatomical constraints imposed by tumors and cortical geometry.

However, we do acknowledge that extremely localized stress concentrations at sub-voxel scales cannot be fully resolved under current MRE resolution limits. We have added a description of limitation to the Discussion section:

"The achievable spatial resolution of the proposed stress-mapping framework is primarily determined by the spatial resolution of the underlying MRE acquisition, therefore, extremely localized stress concentrations at sub-voxel scales cannot be fully resolved under current MRE resolution limits." (Lines 577-580).

(3) In the simulation studies, how is the direction of the vector a defined, given that the stress state is multiaxial?

Response: Thank you for pointing this out. We apologize for the confusion. The vector $\boldsymbol{a}$ is defined as the normalized eigenvector associated with the third primary principal stretch λ_3. As demonstrated in our new multiaxial simulations (**Supplementary Note 5 and Fig. S3**), even when secondary stresses are present (up to 33% of the primary stress), the direction of this principal axis remains stable and carries the leading-order acoustoelastic signature. By aligning $\boldsymbol{a}$ with this dominant eigenvector, our framework can capture the primary deviatoric stress. This approach ensures that the $\boldsymbol{a}$ is uniquely and consistently determined from the intrinsic deformation structure, even under complex multiaxial loading. We have added explanations of the direction accordingly.

The supplementary description of the principal stress directions for the simulation setup of hemispherical shell and embedded sphere models is provided as follows:

"In all cases, the stress field was first established during a quasi-static loading stage, where the vector $\boldsymbol{a}$ is defined as the normalized eigenvector corresponding to the third principal stretch λ_3. This stress field was then probed via low-amplitude dynamic shear excitations, surface displacements were recorded subsequently and used for inversion calculations based on the analytical relations given in Eqs. (3–4)." (Lines 681-686).

The supplementary description of the principal stress directions for the experimental setup for spherical indentation in FEM modelling and MRE measurements is provided as follows:

"For the inversion of both FEM and MRE data, the vector $\boldsymbol{a}$ is uniformly defined as the normalized eigenvector corresponding to the third principal stretch λ_3 from FEM simulations." (Lines 714-716).

(4) In the inflation test of the hemispherical shell, the circumferential stress should be dominant, yet only the radial stress is evaluated. Why was the circumferential stress not considered?

Response: Thank you for pointing this out. We did not explicitly calculate the circumferential stress, because they are not independent parameters and can be derived based on the radial stress. For an incompressible material, the product of the principal stretches is 1 ($\lambda_1 \cdot \lambda_2 \cdot \lambda_3 = 1$). The inflated shell undergoes equi-biaxial stretching in the circumferential plane ($\lambda_{circ1} = \lambda_{circ2} > 1$) and a corresponding radial compression ($\lambda_{radial} < 1$). Mathematically, $\lambda_{circ1} = \lambda_{circ2} = \frac{1}{\sqrt{\lambda_{radial}}}$. Therefore, once the radial principal stretch is obtained, the circumferential stress can be derived. In addition, the anisotropy of wave speed is fundamentally driven by the principal stretch differences; since $\lambda_1 = \lambda_2$, the radial direction is the only axis that defines this anisotropy of wave speed.

Based on the hemispherical shell inflation simulation (now shown in **Fig. 2**), we plotted the principal stretches (λ_1, λ_2 and λ_3) (**Fig. R1**), and observed:

- Symmetry: $\lambda_1 = \lambda_2$, confirming the equi-biaxial circumferential stretch.
- Eigenvector alignment: The direction corresponding to the third principal stretch λ_3 is perfectly aligned with the radial direction, as illustrated in Fig. 2.

This confirms that the radial direction is the unique principal axis leading to the loss of material isotropy, and evaluating the radial component can provide a complete characterization of the stress-induced anisotropy of wave speed for inversion.

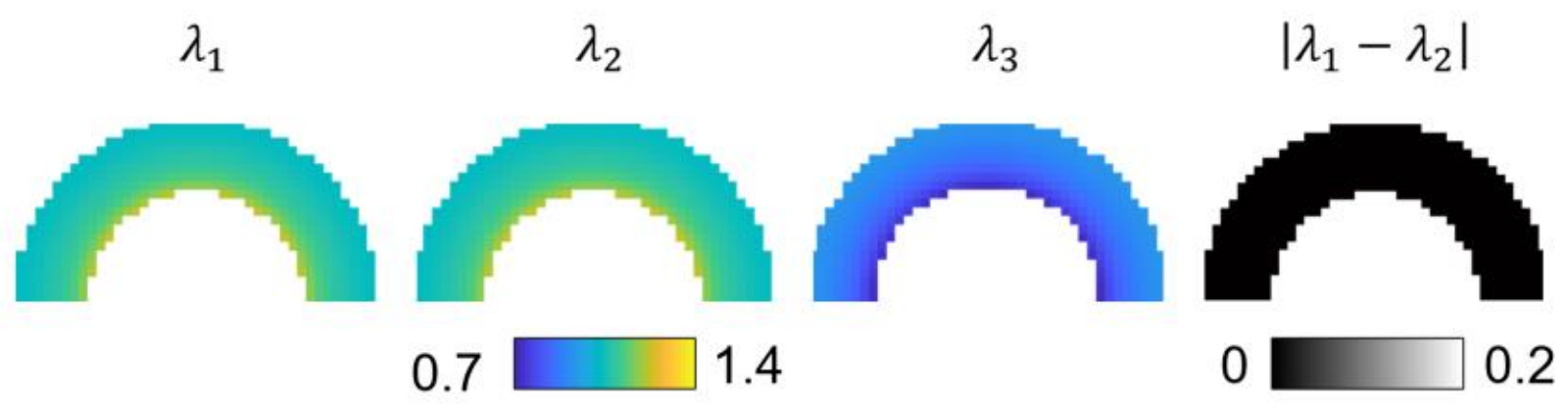

Fig. R1: Distribution of the three principal stretches under large deformation in the hemispherical shell inflation simulation, and the absolute difference between the first and second principal stretches.

(5) In Fig. 4d, the MRE wave field exhibits a longer wavelength compared with the simulated field. Can the authors explain this discrepancy? Additionally, how were the

constitutive parameters chosen for the simulations? This point is particularly important because the ground-truth stress in the phantom experiments is also obtained through simulations.

Response: We appreciate the reviewer's careful observation. The wavelength discrepancy arises from a slight difference in the shear modulus (μ) between the physical phantom and the FEM model. With a predefined modulus of 500 Pa, the FEM simulation had a reconstructed mean of ~517 Pa, the MRE experiment yielded a slightly higher modulus of ~554 Pa (**Fig. R2**). These induced minor variations of wavelength.

In addition, the deformation field (stretch ratio λ) is primarily governed by the boundary conditions (indentation depth and geometry) rather than the absolute magnitude of the modulus. Based on the FEM-derived λ, we effectively decouple the validation from potential errors in material parameter calibration. This ensures that our algorithm is strictly evaluated on its ability to recover the deformation state from the wavefield, regardless of baseline stiffness mismatches.

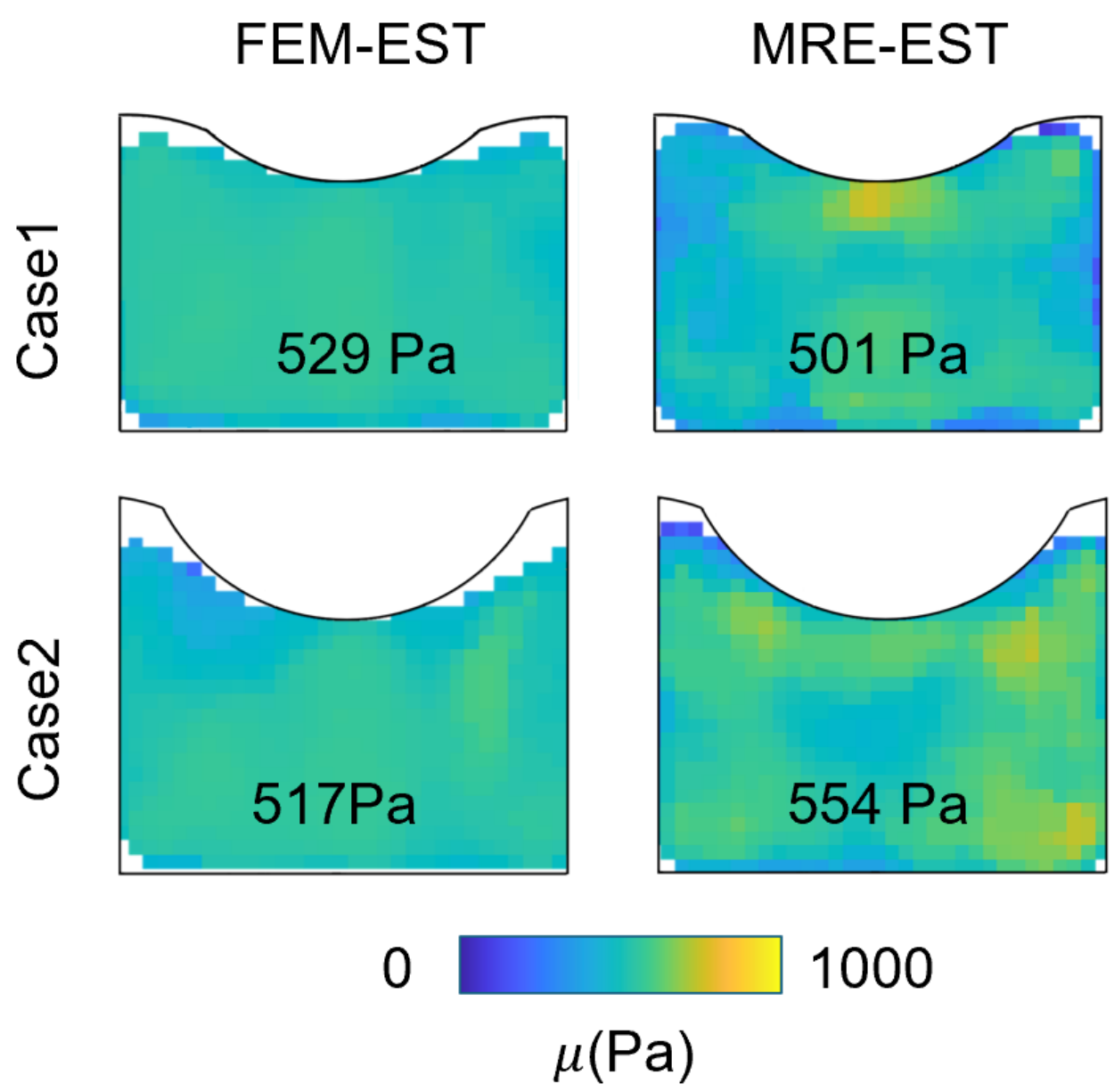

Fig. R2: The mean shear modulus for FEM and MRE experiments.

(6) For non-invasive estimation of intracranial pressure (ICP), the hydrostatic pressure of the cerebrospinal fluid induces an isotropic stress in brain tissue. For incompressible materials, however, hydrostatic loading does not change shear-wave speed. Could the authors clarify this apparent contradiction?

Response: Thank you for pointing this out. The brain tissue is anchored in intracranial cavity and constrained by rigid structures such as the skull, the tentorium cerebelli, and

the falx cerebri. The interaction between the CSF pressure and these anatomical constraints can produce deformation of the brain tissue as illustrated in **Fig. 2**b. This will generate deviatoric stress components and finite local strains. These deviatoric stresses can cause the changes in shear-wave velocity captured by MRE.

To clarify this mechanism and resolve the apparent contradiction, we have added a dedicated section in **Supplementary Note 5** with a new multiaxial simulation validation (**Fig. S3**). In this simulation, we fixed the axial stress σ_z and increased the isotropic lateral pressure ($\sigma_x = \sigma_y$). The results (**Fig. S3**b) confirm that our inversion specifically filters out the hydrostatic (isotropic) bias and accurately extracts the deviatoric normal stress. This demonstrates that by measuring the pre-stress state of the tissue induced by the ICP, we used the normal stress components at the brain surface as an indicator for ICP.

We have also added explanations to the ICP methodology section:

"In the ICP measurement cohort, we defined a 3-voxel (9 mm) inward shell from the cortical surface to capture the stress distribution across the brain's outer layer. This depth was optimized to focus on the brain-CSF interface, which can be analysed using the proposed model. Brain masks were obtained via FSL skull-stripping[14], and the cortex was treated as an elastic boundary. At this interface, stress continuity links the brain tissue stress state to ICP: the hydrostatic pressure of the CSF is balanced by the normal stress of the adjacent brain tissue. As demonstrated numerically in **Supplementary Note 5**, our inversion scheme is insensitive to the hydrostatic component and specifically recovers the deviatoric normal stress, which provides a direct estimate of global ICP. Stress directions were assumed to follow the inward surface normals, and the mean stress magnitude within this cortical shell served as a surrogate for global ICP[15], thereby minimizing the influence of local modelling mismatches. " (Lines 795-807).

(7) There is a substantial gap between the phantom and in vivo experiments due to their markedly different mechanical properties. The gelatin phantom is isotropic and elastic, whereas brain tissue is anisotropic and viscoelastic. Is it possible to validate the in vivo measurements using independent techniques? For example, in the ICP experiments, do the authors have access to data obtained using gold-standard measurement methods?

Response: Thank you for pointing this out. We acknowledge that brain parenchyma exhibits complex anisotropic and viscoelastic properties. While numerous sophisticated anisotropic and viscoelastic models have been proposed, simplified isotropic models capable of capturing its primary biomechanical behavior remain

widely adopted in both scientific research and clinical practice[16–18]. Balancing model realism with analytical and computational feasibility is an ongoing pursuit. As an initial step, we employed the assumption of isotropy and validated the proposed stress estimation method using phantom experiments and simulations, which yielded satisfactory results. Preliminary clinical applications further demonstrate the method's potential. Importantly, acquiring a gold-standard *in vivo* stress field non-invasively in patients remains a significant technical challenge. Given this constraint, we adopted a pragmatic and reliable strategy by performing indirect clinical and physiological validation across three independent clinical scenarios. This multi-scenario approach was designed to enhance the credibility and accuracy of our stress measurements.

We analyzed meningiomas at different locations and found that the calculated stress distribution patterns were highly consistent with the known spatial constraints and geometric deformations imposed by tumors on surrounding tissues, confirming the physical rationality of our results. For patients with pituitary adenomas, the estimated stress magnitude showed a clear correlation with the severity of visual impairment, effectively linking the quantitative stress biomarker proposed in this study to clinical outcomes. In the exploratory ICP estimation experiment, we included populations with varying ages and cognitive statuses, as well as a cohort of patients with NPH. The results showed significant differences in estimated ICP among different groups, which were highly consistent with established physiological and pathological prior knowledge.

Regarding the reviewer's insightful question on the validation of *in vivo* ICP measurements against a gold standard, we would like to clarify that current gold-standard ICP assessment relies on invasive methods, such as lumbar puncture (LP) or intracranial catheter monitoring. These procedures are typically indicated for patients with suspected or confirmed elevated ICP and are not routinely performed on individuals with normal or low ICP. Given these clinical constraints, a direct, invasive comparison in vivo is not ethically or practically feasible for the population in this study. To overcome this limitation, we adopted a multidimensional validation strategy across several independent clinical scenarios. This approach establishes correlative links between our non-invasive measurements and relevant clinical/physiological indicators, thereby providing robust indirect validation of the method's reliability and accuracy.

Reviewer #2:

This manuscript presents an interesting framework for estimating tissue stress from reverberant shear wave fields measured with MRE. The analytical approach is clever and supported by simulations and phantom studies. It is also applied to in vivo image data with some interesting results. The topic is timely and of interest to elastography and tissue mechanics. However, the method relies on specific modeling assumptions which are not consistently and clearly presented. Further, the title and text overstate (3D stress) what is estimated (uniaxial stress). Also, the manuscript does not convincingly discuss how stress-induced anisotropy can be delineated from tissue anisotropy or heterogeneity. This is particularly relevant when moving to the in vivo conditions where the fundamental assumptions of isotropy are violated. Clarifying these issues and more fully characterizing methodological robustness would strengthen the work.

Response: We sincerely thank the reviewer for the constructive and thoughtful feedback. To address the concerns raised, we have significantly revised the manuscript to clarify our modeling framework and its underlying assumptions. A detailed point-by-point response to each specific issue follows below. The corresponding changes in the manuscript are marked in blue.

Primary Comments

Significance: The authors address an interesting inverse tissue mechanics problem in an interesting and clever new way. That said, the contribution would benefit from deeper context relative to prior work. Material anisotropy and structural heterogeneity would seem capable of generating directional wave signatures similar to those attributed to uniaxial stress. The manuscript does not demonstrate that this characterization is fundamentally more informative than providing an alternative characterization, e.g. wave fields based on material anisotropy.

Response: We thank the reviewer for this insightful comment. We agree that brain tissue, particularly white matter, exhibits mechanical anisotropy[9]. However, our results demonstrate that the anisotropy in wave speed induced by stress is the dominant contributor, exceeding the influence of mechanical anisotropy. To further substantiate this point, we have performed additional computational analyses, which are now presented in a new Supplementary Note:

"**Supplementary Note 6: Stress is the primary contributor for differences in wave speed**

The proposed stress-estimation framework leverages directional shear-wave

speed variations. However, brain tissue—particularly white matter with its organized fiber tracts—exhibits mechanical anisotropy, which could confound stress estimation [10]. To isolate the effect of stress, it is necessary to distinguish and compare the influences of mechanical anisotropy against those of stress-induced changes. Accordingly, we compared profiles of the apparent shear modulus ($G_{app} = \rho c_{app}^2$) obtained from an anisotropic constitutive model[11] with those arising from stress-induced states. For this analysis, we adopted a Nearly Incompressible Transversely Isotropic (NITI) model to evaluate the influence of mechanical anisotropy.

As shown in **Fig. S4a,b**, with an applied stress of 1.5 kPa, the angular variation of the apparent shear modulus (G_{app}) for both SV and SH waves significantly exceeds the variation attributable to mechanical anisotropy (using representative brain anisotropic parameters: shear anisotropy $\varphi = 0.1$, tensile anisotropy $\zeta = 0.06$[11]). Under stresses of 1.5 kPa and 3 kPa, the ratio of maximum to minimum G_{app} for SH/SV waves reaches approximately 1.5/2.1 and 2.1/3.7, respectively. In contrast, for an NITI material without applied stress, this ratio remains relatively low (~1.1/1.03; **Fig. S4c**). For ICP estimation (e.g., ~500 Pa), we use the global spatial average of stress over the entire cortical surface rather than local pointwise measurements. This large-scale averaging mitigates direction-dependent biases arising from local fiber tracts, ensuring that the estimated ICP reflects systemic pressure rather than localized tissue microstructure. Collectively, these results demonstrate that the observed directional wave-speed differences are dominated by applied stress, with minimal contribution from the intrinsic mechanical anisotropy of brain tissue.

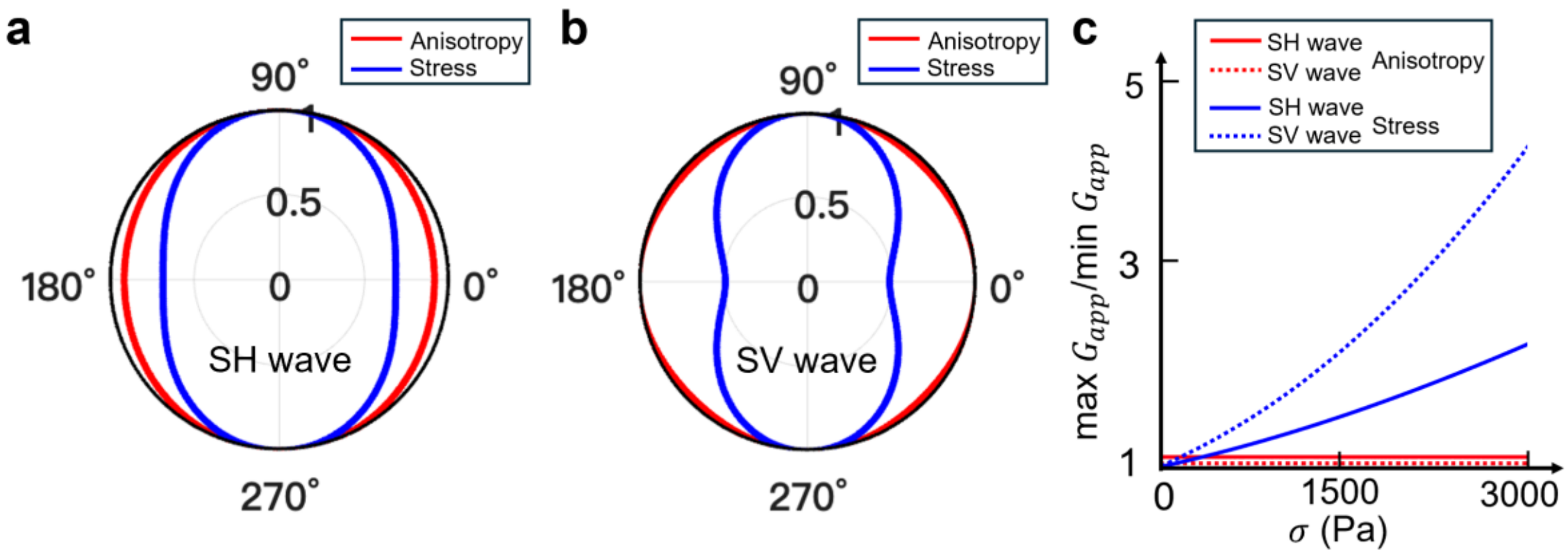

Fig. S4: Comparison of the apparent shear modulus ($G_{app} = \rho c_{app}^2$) due to applied stress versus intrinsic mechanical anisotropy. **a, b** Normalized apparent shear modulus as a function of the angle between wave-propagation direction and the fiber/principal-stress axis, shown for **(a)** SH and **(b)** SV waves. **c** Ratio of maximum to minimum G_{app} ($G_{app,max}$ / $G_{app,min}$) under increasing applied stress, using an anisotropic material without applied stress as the baseline. The pronounced increase

in the ratio confirms that applied stress, rather than intrinsic mechanical anisotropy, is the dominant source of wave-speed variation.*"* (in Supplementary Information).

We have also added the following content in the Discussion section:

"Several potential sources of modeling bias are examined in the Supplementary Information. **Supplementary Note 6** compares wave-speed variations induced by stress with those arising from tissue mechanical anisotropy, confirming that acoustoelastic effects dominate directional wave-speed changes." (Lines 587-590).

Conclusions and Claims: The title, "Noninvasive mapping of 3D brain stress…", is misleading. The method estimates a single axial stress component, not a full 3D stress tensor. This distinction should be reflected in the title and throughout the manuscript to avoid overstating the capability of the approach. Further it is not clear why 'brain' is in the title. Brain imaging seems to be more of a potential application than the focus of the paper.

Response: Thank you for pointing this out. To be more precise and highlight the proposed method's broader applicability beyond brain, we have revised the title to:

"Noninvasive 3D mapping of tissue stress using reverberant shear wave",

which emphasizes the 3D spatial mapping of stress distribution while being more prudent regarding tensor components.

Accordingly, we have revised the manuscript to ensure that we do not claim to reconstruct the 3D stress tensor. For the Discussion section:

"While our method demonstrates promising capabilities, it is subject to certain limitations. The full stress state in tissue is described by a second-order tensor comprising six independent components, making the recovery of all components from a single shear-wave measurement impractical. Under the incompressibility assumption, shear-wave propagation is insensitive to hydrostatic pressure, leaving the full three-dimensional tensor theoretically underdetermined. To render the problem tractable, we reduced the degrees of freedom by adopting an isotropic hyperelastic material model and focusing our analysis on the dominant uniaxial stress component. This simplification aligns with physiological observations where loading is predominantly normal to tissue boundaries[2,3].Although these assumptions may not fully capture stress complexity in regions subjected to multiaxial loading, we demonstrate that the inversion specifically extracts the deviatoric stress difference while remaining insensitive to hydrostatic bias (**Supplementary Note 5**)." (Lines 564-576).

For the Abstract:

"Employing a physics-informed model, we analyze reverberant shear wave fields recorded by MRE to capture the 3D mapping of tissue stress." (Lines 40-41).

For the Results section:

"Here, we present the first high-resolution 3D mapping of normal stress within the optic chiasm in four patients with pituitary adenoma, acquired by combining MRE with wave-based inversion." (Lines 369-371).

Figure Captions:

"**Fig. 5**: In vivo measurement of normal stress induced by meningiomas." (Line 349).

"**Fig. 6:** Normal stress measurement results of optic chiasm compression by pituitary adenoma." (Lines 391-392).

Subtitles:

"In vivo normal stress measurement of meningioma" (Line 294).

"Quantitative mapping of optic chiasm normal stress in pituitary adenomas" (Line 359).

Methodology: The inversion assumes near-incompressibility, isotropy, and uniaxial stress. When applied in vivo, there also seems to be a need to define the primary stress direction. These assumptions fundamentally affect the interpretation of both phantom and in vivo data and should be presented more clearly throughout the paper. It also seems that these assumptions do not hold in some of the situations studied. For example, pressure on a curved tissue induces stress in the lateral direction. The authors should more rigorously define what is meant by a dominant uniaxial stress component. Further, it seems that intrinsic anisotropy, microstructural organization, and boundary effects could produce similar polarization-dependent wave phenomena, and more rigorous discussion or testing of these alternative explanations would be helpful.

Response: We agree that in vivo stress fields are generally multiaxial. However, our inversion is designed to capture the primary deviatoric stress component, which is the main contributor of wave speed differences. We have added a new multiaxial simulation study in **Supplementary Note 5** using a triaxial cube model (**Fig. S3**). The results demonstrate that even in a multiaxial stress state, where lateral stresses reach 1/3 of the primary stress, the estimation error for the primary stress remains below

10%. For explanation of the primary uniaxial stress component, we have added in **Supplementary Note 5**:

"**Supplementary Note 5: Assumption of uniaxial stress for *in vivo* tissue stress estimation**

In this study, stress inversion is formulated under a uniaxial-dominant assumption, where the local stress state is represented by an effective stretch ratio along a primary axis. Although multiaxial stress state may exist, this Note clarifies that the assumption can still provide satisfactory estimation. If brain tissue is described as a nearly incompressible soft solid, the Cauchy stress tensor is decomposed into a hydrostatic component and a deviatoric component. Classical nonlinear acoustoelastic theory demonstrates that shear waves are insensitive to hydrostatic pressure and respond exclusively to the deviatoric part, which governs stress-induced anisotropy[1]. Consequently, isotropic pressure, while potentially large in magnitude, does not contribute to measurable shear-wave speed variation. This property fundamentally limits what components of a multiaxial stress field can be observed.

To quantify the influences of multiaxiality and hydrostatic pressure, we performed numerical simulations in COMSOL using a cubic specimen with dimensions of 50×50×50 mm^3. The phantom was modelled as a Mooney-Rivlin material with material parameters $C_{01} = 400\text{Pa}$ and $C_{10} = 600\text{Pa}$. As illustrated in **Fig. S3**a, roller supports were set on three adjacent surfaces, while independent compressive stresses $(\sigma_x, \sigma_y, \sigma_z)$ were applied to the other three surfaces. Shear vibrations were then exerted on the remaining three surfaces at a frequency of 50 Hz. The wavefield analysis procedures adopted in this study were consistent with those detailed in **Fig. 2**. For the stress inversion, the z-axis was specified as the principal stress orientation. In the z-axis, the stretch ratio λ was derived from the simulation, while the ground-truth stress σ_{GT} was further obtained using Eq. S8.

- **2D Multiaxial Loading** $(\sigma_z > \sigma_x, \sigma_y = 0)$: We fixed $\sigma_y = 0$, $\sigma_z = 1500\text{Pa}$ and increased σ_x from 0 to 500 Pa (lateral stress ratio σ_x/σ_z up to 1/3). While the estimation error increases with multiaxiality, it remains below 5% even when the lateral stress is 1/3 of the primary stress (**Fig. S3**b). For complex scenarios, the state can be viewed as a superposition of a hydrostatic part and a uniaxial-dominant deviatoric part, which our method recovers with acceptable accuracy.
- **3D Symmetric Loading** $(\sigma_z > \sigma_y = \sigma_x)$: We verified the system's insensitivity to hydrostatic pressure by fixing $\sigma_z = 1500\text{Pa}$ and increasing the lateral pressure $\sigma_y = \sigma_x$ from 0 to 500 Pa. In this configuration, the theoretically observable

stress corresponds to the deviatoric difference $\sigma_{diff} = \sigma_z - \sigma_y$. The results show that the relative error between σ_{GT} and σ_{diff} is negligible (<4e-4), confirming that the stress derived from the deformation state is equivalent to the principal stress difference (Eq. S8). Furthermore, the estimated stress σ_{EST} obtained through our wave-based inversion remained highly consistent with σ_{diff}, with a relative error below 1% (**Fig. S3**c). This dual verification shows that the proposed framework effectively filters out hydrostatic pressure bias and specifically resolves the deviatoric stress components that reflect the actual compressive state of the tissue.

- **3D Asymmetric Loading**: We performed a parameter sweep where both σ_x and σ_y independently varied from 0 to 500Pa in steps of 50 Pa (fix $\sigma_z = 1500\text{Pa}$). As shown in **Fig. S3**d, our method consistently recovers the primary stress σ_z across all combinations, with a maximum absolute error rate below 8.6% (130 Pa).

Reconstructing a full stress tensor from clinical MRE is often ill-posed. By capturing the dominant deviatoric component, we are able to provide a stable and practical metric that preserves directional accuracy without the numerical instability of over-parameterized models.

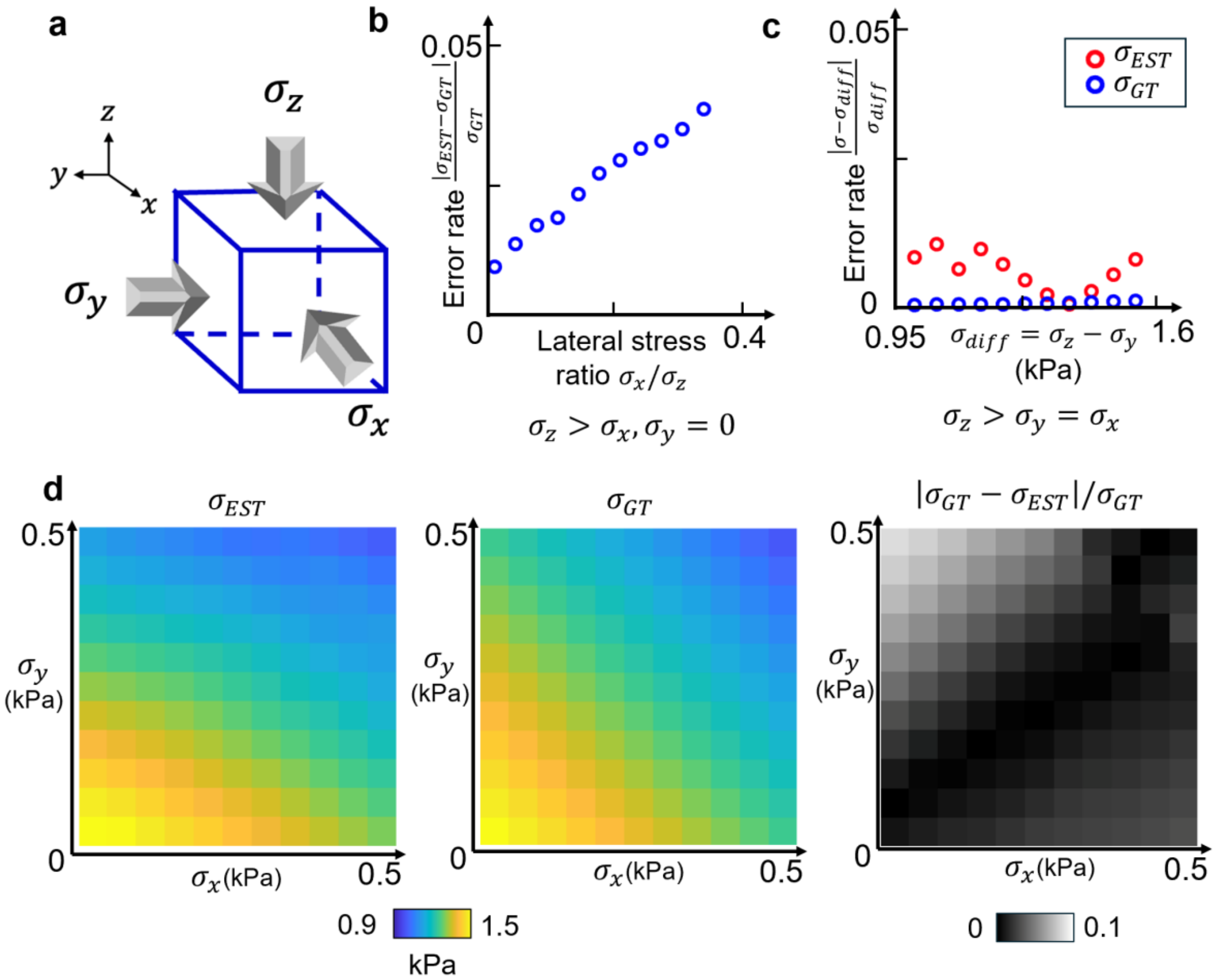

Fig. S3. Validation of the stress estimation under multiaxial stress conditions. **a** A 50×50×50 mm^3 cube with triaxial compressive loads ($\sigma_x, \sigma_y, \sigma_z$). A Mooney-Rivlin material model was used. **b** 2D Multiaxial Case**:** Estimation error for the stress as a function of lateral stress ratio σ_x/σ_z. **c** 3D Symmetric Case: Relative error of the estimated stress (σ_{EST}, red dots) and the stretch-derived stress (σ_{GT}, blue dots) compared to the theoretical deviatoric difference ($\sigma_{diff} = \sigma_{GT} - \sigma_y$). **d** Full 3D Asymmetric Case: Distribution of estimated stress across a comprehensive sweep of independent σ_x and σ_y (0–500 Pa)." (in Supplementary Information).

We have also added the following content in the Discussion section:

"While our method demonstrates promising capabilities, it is subject to certain limitations. The full stress state in tissue is described by a second-order tensor comprising six independent components, making the recovery of all components from a single shear-wave measurement impractical. Under the incompressibility assumption, shear-wave propagation is insensitive to hydrostatic pressure, leaving the full three-dimensional tensor theoretically underdetermined. To render the problem tractable, we reduced the degrees of freedom by adopting an isotropic hyperelastic material model and focusing our analysis on the dominant uniaxial stress component. This simplification aligns with physiological observations where loading is predominantly normal to tissue boundaries[2,3].Although these assumptions may not fully capture stress complexity in regions subjected to multiaxial loading, we demonstrate that the inversion specifically extracts the deviatoric stress difference while remaining insensitive to hydrostatic bias (**Supplementary Note 5**)." (Lines 564-576).

Regarding brain anisotropy, we have previously shown in **Supplementary Note 6** that stress-induced anisotropy (anisotropy ratio: max/min apparent modulus ~1.5–3.7) is much higher than the mechanical anisotropy of brain (anisotropy ratio ~1.03–1.1).

Regarding the potential interference from microstructural organization and boundary effects, we clarify as follows:

1) Microstructure: We consider wave anisotropy arising from microstructural organization to be functionally equivalent to the mechanical anisotropy discussed in **Supplementary Note 6**.
2) Boundary effects: While complex structures like the skull cause extensive reflection and refraction, our framework is specifically built upon reverberant shear wave fields. Rather than being a limitation, these complex wave interactions provide the diffuse, multi-directional wave information that our inversion relies on to resolve stress components.

Robustness: Simulations and phantoms show internal consistency. However, the method's sensitivity to realistic MRE error sources is not quantified. How does displacement noise and spatial resolution propagate into stress estimates? Without such analyses, it is difficult to determine whether observed clinical trends reflect physiology or algorithmic artifacts.

Response: Thank you for pointing this out. For the potential noise pollution, we conducted comprehensive simulations with noise using both homogeneous and heterogeneous models. By introducing 2% to 6% Gaussian noise into the displacement wave fields, we quantified the estimation errors. Results show that the inversion framework is resilient to noise, with errors remaining within approximately 8%–15%, even at high noise levels. We have added a detailed analysis of robustness, including simulation methods and error quantification, in the Supplementary Notes:

"**Supplementary Note 8: Robustness of stress estimation to noise**

To evaluate the sensitivity of the proposed stress-inversion framework to realistic MRE error sources, we performed systematic noise propagation analyses. Gaussian noise at levels of 2%, 4%, and 6% was incorporated into the simulated displacement wave fields of the pressurized hemispherical shell and the heterogeneous embedded-sphere inflation models. For simulations with noisy wave fields, 3D Gaussian filtering (kernel=3x3x3, variance = 1) was applied prior to inversion.

The results demonstrate that the algorithm maintains high accuracy and stability even under significant noise interference (**Fig. S6**). For the homogeneous shell model, the relative estimation errors were 7.92%, 10.8%, and 15.5% for noise levels of 2%, 4%, and 6%, respectively, compared to a baseline error of 2.09% in the noise-free case. In the more complex heterogeneous scenario, the errors remained stable at 8.17%, 8.74%, and 10.1% (baseline error: 7.84%). A localized error increase observed near 150° in the heterogeneous model may be attributed to edge effects of the Gaussian kernel and data truncation at the boundary. These findings indicate that the stress patterns and clinical trends observed in this study are mainly driven by underlying physiological conditions rather than noise or algorithmic artifacts.

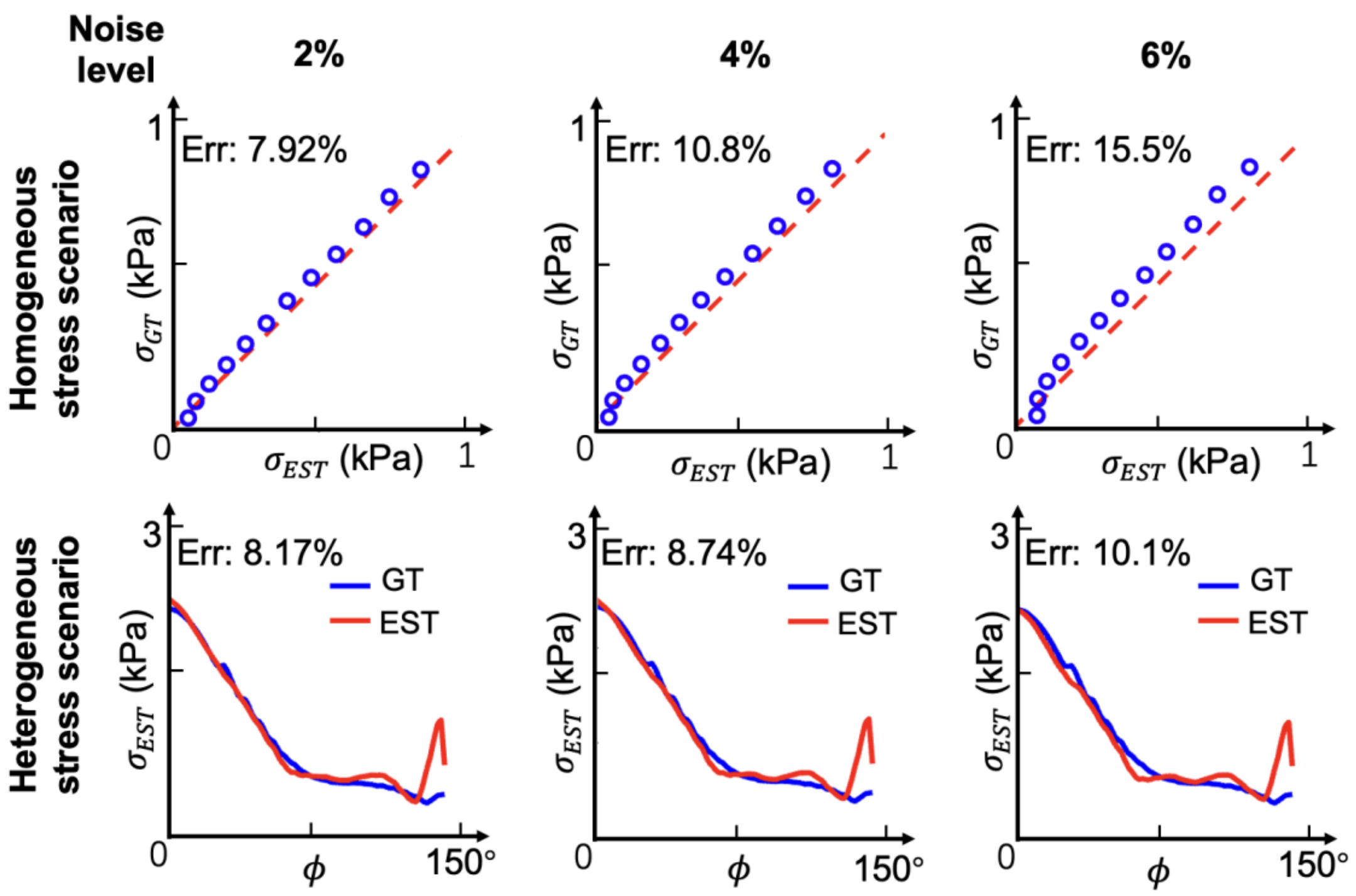

Fig.S6 Stress estimation under increasing measurement noise. Top row (Fig. 2): Pressurized hemispherical shell simulation. Scatter plots compare ground-truth stress (x-axis) with estimated stress (y-axis). The red dashed line indicates the ideal 1:1 correspondence (x = y). Despite rising noise, estimation error increases only moderately (7.92% to 15.5%), while the linear correlation with ground truth remains strong. **Bottom row (Fig. 3):** Heterogeneous embedded-sphere inflation simulation. Stress estimation error remains highly stable, even under 6% noise. Together, these results demonstrate the robustness of the inversion algorithm to realistic levels of measurement noise." (in Supplementary Information).

We have also added the following content in the Discussion section:

"Several potential sources of modeling bias are examined in the Supplementary Information. **Supplementary Note 6** compares wave-speed variations induced by stress with those arising from tissue mechanical anisotropy, confirming that acoustoelastic effects dominate directional wave-speed changes. **Supplementary Note 7** shows that material damping has negligible impact on stress estimation, since the inversion relies primarily on phase-gradient information rather than wave amplitude. **Supplementary Note 8** further demonstrates, through noise-propagation simulations, that the proposed framework remains stable under realistic noise levels." (Lines 587-594).

The influence of spatial sampling is implicitly addressed in our heterogeneous simulation (**Fig. 3**), which was specifically designed to mimic realistic clinical MRE resolution. In this simulation, the original high-resolution displacement fields generated

by finite-element modeling were down-sampled to a 3 mm isotropic spacing prior to stress inversion. Despite this resolution reduction, the proposed framework successfully recovered the heterogeneous prestress distribution on the embedded spherical surface, including its systematic variation along the elevation angle.

Furthermore, as presented in the Supplementary Information, this same heterogeneous configuration was subjected to additional noise, but the heterogeneity of angular stress was still obtained. These results indicate that, although higher spatial resolution is generally advantageous, the commonly used 3 mm resolution in current clinical MRE is sufficient for robust stress estimation. We note that resolving stress variations in substantially smaller targets would indeed benefit from higher spatial resolution; however, such resolution is currently constrained by clinical MRE acquisition capabilities.

Reviewer #3:

This manuscript proposes a physics-based inversion framework that allows direct stress estimation based on reverberant shear wave fields acquired by MRE. It does not rely on wave direction information or characterization. The authors demonstrated this framework could reconstruct the heterogeneous stress distribution accurately on a deformed 3D hemispherical shell model, a simulated sphere inflation embedded in a hyperelastic block, and two rigid hemispherical shells indented gelatin phantoms as well as corresponding COMSOL simulations. In addition, the authors examined this approach on healthy young adults, healthy elderly adults, mild cognitive impairment patients, elderly patients with normal pressure hydrocephalus, patients with intracranial meningioma, and patients with optic chiasm compression by pituitary adenoma. The findings are consistent with current neuropathology and neuroanatomy, and offer new understandings for brain mechanobiology.

Response: We sincerely thank the reviewer for the positive and comprehensive summary of our work. We are encouraged by the recognition of our physics-based stress inversion framework. In the revised manuscript, we have further strengthened the work by addressing the specific points raised to ensure the method's potential for clinical translation is fully supported by rigorous analysis. A detailed point-by-point response to each of your constructive comments follows below. The corresponding changes in the manuscript are marked in blue.

Broad Comments

1. The manuscript would benefit from a sharper delineation of how the proposed method (operator-based separation of SV/SH anisotropy, direction-agnostic inversion, and 3D stress recovery from reverberant MRE fields) differs fundamentally and has advantages from prior acoustoelastic approaches (such as directional acoustoelasticity, orthogonal-wave methods, reverberant elastography). The novelty is present, but the text is a bit too broad. Related work worth citing: doi:10.1088/1361-6560/acbbb7.

Response: We thank the reviewer for the insightful suggestion. We have revised the Discussion to discuss our approach with directional acoustoelasticity, orthogonal-wave methods, and traditional reverberant elastography. The recommended work (doi:10.1088/1361-6560/acbbb7) was cited as well as other relevant studies on reverberant autocorrelation inversion. A paragraph was added to the Discussion section:

"In comparison to previous stress estimation methods, the proposed method

overcomes the physical bottlenecks of conventional acoustoelasticity and model-based elastography. Acoustoelasticity typically requires prescribed wave propagation modes or known wave directions[19–21], which are challenging for brain due to cranial reflections and tissue heterogeneity. While recent orthogonal-wave methods[22] elegantly demonstrate stress recovery in soft materials, the required precise wavefield steering is also challenging for deep and complex tissues. Furthermore, existing reverberant elastography frameworks[23–25] generally assume a statistically isotropic, diffuse wavefield, relying on the expectation over all propagation directions to estimate properties. However, the presence of significant tissue damping and limited excitation sources often results in a "finite" or incomplete reverberance that may not satisfy the statistical assumptions. The proposed method differs fundamentally by embracing these uncontrolled, "finite" reverberant fields. Instead of imposing physical wave control or relying on perfect wavefield isotropy, we utilize an operator-based inversion that mathematically untangles the complex wavefield[11]. Much like a series of calibrated rotations to restore a Rubik's Cube, our tailored sequence of differential operators acts on the diverse wavelets in the reverberant field, isolating stress-induced signatures. Unlike prior methods that attempt physical wavefield pre-conditioning, the proposed method focuses on mathematically de-conditioning the naturally complex wavefield to extract acoustoelastic signatures. This enables us to establish an analytical link between non-cooperative wavefields and 3D stress distributions, without the need for perfect reverberance or material constant calibration. By avoiding the directional filtering required by earlier methods[26], our approach preserves the full anisotropy of the wavefield, enabling robust, high-resolution stress mapping across the entire field of view." (Lines 483-507).

2. The inversion assumes that brain tissue is modeled as an incompressible isotropic hyperelastic matieral. This assumption should be stated prominently in the methods and included in discussion, particularly given that brain regions such as white matter are often modeled as anisotropic.

Response: We thank the reviewer for this insightful comment. We agree that modeling brain tissue as an isotropic, hyperelastic medium is a fundamental simplification. The following revisions were also made to the methodology overview in the Results section:

"The brain is a soft, structurally complex tissue. While numerous models have been proposed to characterize its biomechanical behavior [4], isotropy and incompressibility remain standard simplifying assumptions[5]. Building on these assumptions, we propose a physics-informed traveling wave expansion (TWE) model. Derived from the hyperelastic Mooney–Rivlin constitutive model [5,6], the TWE

framework describes the reverberant wavefield as a linear superposition of shear-vertical (SV) and shear-horizontal (SH) wave components[7,8] propagating along multiple directions (**Fig. 1c**)." (Lines 119-126).

The following revisions were also made to the discussion:

"Several potential sources of modeling bias are examined in the Supplementary Information. **Supplementary Note 6** compares wave-speed variations induced by stress with those arising from tissue mechanical anisotropy, confirming that acoustoelastic effects dominate directional wave-speed changes." (Lines 587-590).

As the reviewer correctly pointed out, brain tissues such as white matter are mechanically anisotropic. From the perspective of an inverse problem, the choice of model complexity must be balanced against the quality of available clinical data and the stability of the solution. If we were to simultaneously incorporate both mechanical anisotropy and prestress, the parameter space would expand drastically[27]: Two independent symmetry axes would need to be defined or resolved (one for the fiber tracts and one for the principal stress direction); A combined model would require estimating at least three anisotropic parameters, the stress tensor components, and the hyperelastic material constants simultaneously. Given the present image acquisition condition, such an over-parameterized inversion would be highly ill-posed, making it nearly impossible to achieve a stable or unique solution. In this work, we focused on the stress-induced changes which, in the pathological scenarios studied, are the dominant mechanical drivers. We believe that providing robust, clinically actionable information on tissue stress is of greater value than pursuing a theoretically "complete" but numerically unstable model.

Admittedly, the mechanical anisotropy of brain tissue exists and could potentially influence stress inversion. We have addressed this by performing a quantitative analysis to clarify the extent of this bias. As detailed in our response to your next question and in **Supplementary Note 6**, we demonstrate that in high-stress pathological regimes, the acoustoelastic effect significantly outweighs the influence of mechanical anisotropy.

3. As anisotropic MRE methods are emerging, it is worth mentioning how stress-induced anisotropy is separatable from intrinsic fiber anisotropy in brain tissue. While I agree that it is distinguishable, readers might appreciate the clarification.

Response: We thank the reviewer for this insightful comment. We agree that brain tissue, particularly white matter, exhibits mechanical anisotropy[9]. However, our results

demonstrate that the anisotropy in wave speed induced by stress is the dominant contributor, exceeding the influence of mechanical anisotropy. To further substantiate this point, we have performed additional computational analyses, which are now presented in a new Supplementary Note:

"**Supplementary Note 6: Stress is the primary contributor for differences in wave speed**

The proposed stress-estimation framework leverages directional shear-wave speed variations. However, brain tissue—particularly white matter with its organized fiber tracts—exhibits mechanical anisotropy, which could confound stress estimation [10]. To isolate the effect of stress, it is necessary to distinguish and compare the influences of mechanical anisotropy against those of stress-induced changes. Accordingly, we compared profiles of the apparent shear modulus ($G_{app} = \rho c_{app}^2$) obtained from an anisotropic constitutive model[11] with those arising from stress-induced states. For this analysis, we adopted a Nearly Incompressible Transversely Isotropic (NITI) model to evaluate the influence of mechanical anisotropy.

As shown in **Fig. S4a,b**, with an applied stress of 1.5 kPa, the angular variation of the apparent shear modulus (G_{app}) for both SV and SH waves significantly exceeds the variation attributable to mechanical anisotropy (using representative brain anisotropic parameters: shear anisotropy φ = 0.1, tensile anisotropy ζ = 0.06[11]). Under stresses of 1.5 kPa and 3 kPa, the ratio of maximum to minimum G_{app} for SH/SV waves reaches approximately 1.5/2.1 and 2.1/3.7, respectively. In contrast, for an NITI material without applied stress, this ratio remains relatively low (~1.1/1.03; **Fig. S4c**). For ICP estimation (e.g., ~500 Pa), we use the global spatial average of stress over the entire cortical surface rather than local pointwise measurements. This large-scale averaging mitigates direction-dependent biases arising from local fiber tracts, ensuring that the estimated ICP reflects systemic pressure rather than localized tissue microstructure. Collectively, these results demonstrate that the observed directional wave-speed differences are dominated by applied stress, with minimal contribution from the intrinsic mechanical anisotropy of brain tissue.

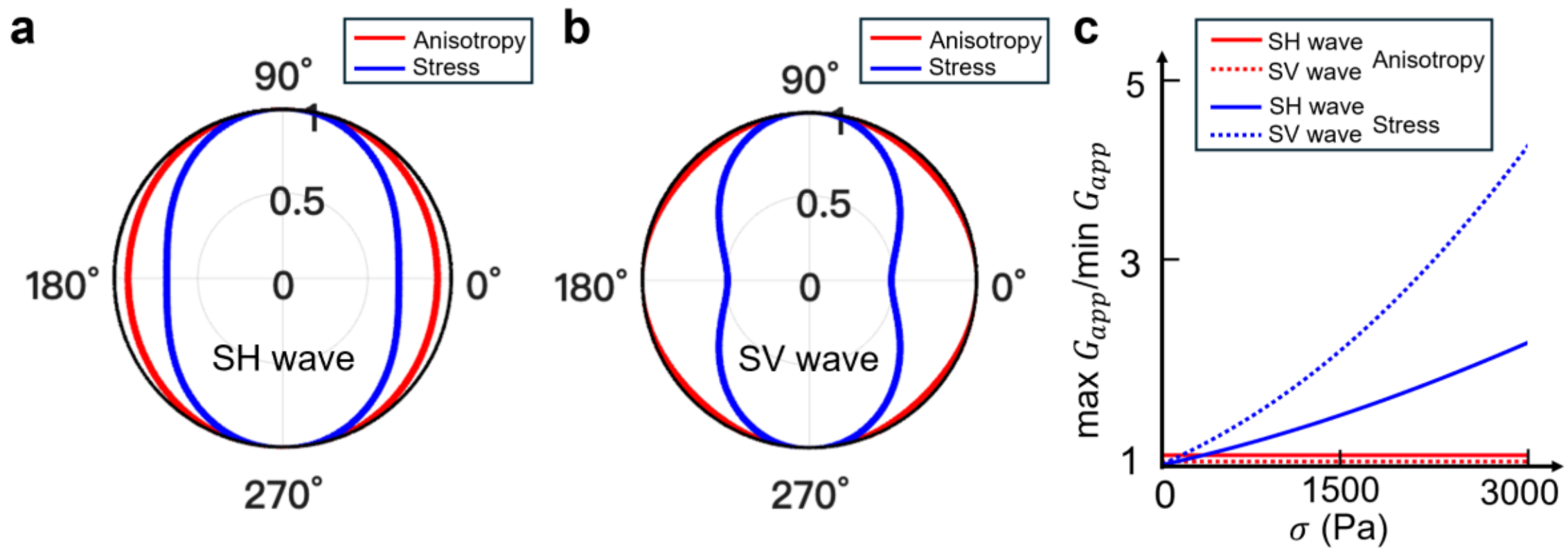

Fig. S4: Comparison of the apparent shear modulus ($G_{app} = \rho c_{app}^2$) due to applied stress versus intrinsic mechanical anisotropy. **a, b** Normalized apparent shear modulus as a function of the angle between wave-propagation direction and the fiber/principal-stress axis, shown for **(a)** SH and **(b)** SV waves. **c** Ratio of maximum to minimum G_{app} ($G_{app,max}$ / $G_{app,min}$) under increasing applied stress, using an anisotropic material without applied stress as the baseline. The pronounced increase in the ratio confirms that applied stress, rather than intrinsic mechanical anisotropy, is the dominant source of wave-speed variation.*"* (in Supplementary Information).

We have also added the following content in the Discussion section:

"Several potential sources of modeling bias are examined in the Supplementary Information. **Supplementary Note 6** compares wave-speed variations induced by stress with those arising from tissue mechanical anisotropy, confirming that acoustoelastic effects dominate directional wave-speed changes." (Lines 587-590).

4. I believe that from a single frequency reverberant field, the full 3D stress tensor is not identifiable, and only the dominant principal stress component aligned with an assumed direction can be recovered. The manuscript clarify which tensor components are theoretically observable and which are not.

Response: Thank you for pointing this out. To be more precise and highlight the proposed method's broader applicability beyond brain, we have revised the title to:

"Noninvasive 3D mapping of tissue stress using reverberant shear wave",

which emphasizes the 3D spatial mapping of stress distribution while being more prudent regarding tensor components.

Accordingly, we have revised the manuscript to ensure that we do not claim to reconstruct the 3D stress tensor. For the Discussion section:

"While our method demonstrates promising capabilities, it is subject to certain limitations. The full stress state in tissue is described by a second-order tensor comprising six independent components, making the recovery of all components from a single shear-wave measurement impractical. Under the incompressibility assumption, shear-wave propagation is insensitive to hydrostatic pressure, leaving the full three-dimensional tensor theoretically underdetermined. To render the problem tractable, we reduced the degrees of freedom by adopting an isotropic hyperelastic material model and focusing our analysis on the dominant uniaxial stress component. This simplification aligns with physiological observations where loading is predominantly normal to tissue boundaries[2,3].Although these assumptions may not fully capture stress complexity in regions subjected to multiaxial loading, we demonstrate that the inversion specifically extracts the deviatoric stress difference while remaining insensitive to hydrostatic bias (**Supplementary Note 5**)." (Lines 564-576).

For the Abstract:

"Employing a physics-informed model, we analyze reverberant shear wave fields recorded by MRE to capture the 3D mapping of tissue stress." (Lines 40-41).

For the Results section:

"Here, we present the first high-resolution 3D mapping of normal stress within the optic chiasm in four patients with pituitary adenoma, acquired by combining MRE with wave-based inversion." (Lines 369-371).

Figure Captions:

"**Fig. 5**: In vivo measurement of normal stress induced by meningiomas." (Line 349).

"**Fig. 6:** Normal stress measurement results of optic chiasm compression by pituitary adenoma." (Line 391).

Subtitles:

"In vivo normal stress measurement of meningioma" (Line 294).

"Quantitative mapping of optic chiasm normal stress in pituitary adenomas" (Line 359).

5. The differential operators are the key to the method, so I feel the explanation could be strengthened. Please provide more rigorous reasoning showing how these operators act differently on SV and SH components, why they yield distinct angular

weightings of μA, μB, μC, and how this allows for local inversion. Full derivation is definitely not necessary, but a bit more mathematical rationale would strengthen the paper.

Response: We appreciate the reviewer's request for a more rigorous explanation. We have strengthened the explanation of differential operators in the Methods section to provide a clearer mathematical and physical picture for the readers:

"To overcome these challenges, a differential-operator-based inversion framework was formulated to directly extract stress information from the composite wavefield. The fundamental mathematical rationale of this framework is to avoid solving the complex, nonlinear wave equations under large deformation. Instead, we leverage the known analytical characteristics of wave propagation, e.g. polarization and velocity modulation. In addition, a specific set of operators are also used to analytically "substitute" the unknown terms, such as local propagation directions and modulation angles, within the reverberant field. Physically, these operators exploit the vector identities between the wavefield $\boldsymbol{u}$ and the stress axis $\boldsymbol{a}$. Since SH polarization $\boldsymbol{m}_{SH}$ is inherently orthogonal to $\boldsymbol{a}$ ($\boldsymbol{a} \cdot \boldsymbol{m}_{SH} = 0$), whereas SV polarization $\boldsymbol{m}_{SV}$ has a projection onto $\boldsymbol{a}$ that varies with the propagation direction, operators involving ($\boldsymbol{a} \cdot \boldsymbol{U}$) effectively act as polarization filters to differentiate the two modes. Furthermore, directional derivatives of the form $(\boldsymbol{\nabla} \cdot \boldsymbol{a})^2$ incorporate angular weighting factors equivalent to $\cos^2\theta$ for subwave components. By applying different orders of these derivatives, the operators generate distinct, linearly independent angular kernels for the effective moduli μ_A, μ_B and μ_C.

Three kinds of key operators, as detailed in **Supplementary note 2**, were constructed to address the wavefield complexity: 1) Polarization Manipulation $\boldsymbol{\nabla} \times \boldsymbol{U}$: Enabling SV-SH polarization conversion to isolate stress-dependent interactions. 2) Component-Selective Filtering $\boldsymbol{a} \cdot \boldsymbol{U}$: Specifically suppressing SH-wave components and enhancing SV-wave fidelity. 3) Angle-Domain Weighting Modulation $(\boldsymbol{\nabla} \cdot \boldsymbol{a})^2$ and $|(\boldsymbol{\nabla} \times \boldsymbol{a})|^2$: Performing weighted wavefield reconstruction via the θ parameter to modulate sensitivity directionally. Conceptually, these operators act on the reverberant field similar to calibrated rotations used to solve a Rubik's Cube: while each operator induces distinct, synchronized changes across all wavelets based on their specific orientations, their structured combination mathematically untangles the chaotic interference into a solvable analytical relationship. This allows for the local decoupling of the intermediate variables μ_A, μ_B and μ_C, which serve as essential mathematical bridges, to yield a concise, closed-form expression for the uniaxial stress magnitude without requiring explicit wave-mode separation or global wave steering." (Lines 628-656).

6. The physiological link between surface-normal stress and ICP requires more mechanical justification. The relationship might be reasonable, but the manuscript never explains the mechanical link. Please also 1) justify the choice of the 1.5 cm peritumoral shell, and 2) define where the quasi-local homogeneity assumption is expected to hold.

Response: We thank the reviewer for these insightful comments. For the mechanical link between surface-normal stress and ICP, force equilibrium at the fluid-solid interface plays a major role. According to the traction continuity boundary condition, at the interface between the cerebrospinal fluid (CSF) and the brain parenchyma, the normal stress σ_n of the solid tissue must equilibrate with the hydrostatic pressure P_{ICP} of the CSF, such that $\sigma_n + P_{ICP} = 0$ on the cortical surface. As ICP increases, this fluid pressure is transmitted as a compressive pre-stress into the constrained brain matrix. Consequently, by measuring the normal stress distribution at the cortical surface via acoustoelastic MRE, we are able to measure ICP.

To clarify this mechanism and resolve the apparent contradiction, we have added a dedicated section in **Supplementary Note 5** with a new multiaxial simulation validation (**Fig. S3**). In this simulation, we fixed the axial stress σ_z and increased the isotropic lateral pressure ($\sigma_x = \sigma_y$). The results (**Fig. S3**b) confirm that our inversion specifically filters out the hydrostatic (isotropic) bias and accurately extracts the deviatoric normal stress. This demonstrates that by measuring the pre-stress state of the tissue induced by the ICP, we used the normal stress components at the brain surface as an indicator for ICP.

We have added the following description to the Methods section:

"In the ICP measurement cohort, we defined a 3-voxel (9 mm) inward shell from the cortical surface to capture the stress distribution across the brain's outer layer. This depth was optimized to focus on the brain-CSF interface, which can be analysed using the proposed model. Brain masks were obtained via FSL skull–stripping[14], and the cortex was treated as an elastic boundary. At this interface, stress continuity links the brain tissue stress state to ICP: the hydrostatic pressure of the CSF is balanced by the normal stress of the adjacent brain tissue. As demonstrated numerically in **Supplementary Note 5**, our inversion scheme is insensitive to the hydrostatic component and specifically recovers the deviatoric normal stress, which provides a direct estimate of global ICP. Stress directions were assumed to follow the inward surface normals, and the mean stress magnitude within this cortical shell served as a surrogate for global ICP[15], thereby minimizing the influence of local modelling mismatches." (Lines 795-807).

We apologize for any ambiguity regarding the choice of ROI dimensions. The shell thicknesses were meticulously selected to match the physical scale of the pathology and the specific anatomical constraints of each cohort. We have clarified these points at the Methods section:

"A 1.5 cm (5 pixels) isotropic peritumoral shell was defined as the region of interest (ROI), within which the local stress direction was assumed to be normal to the tumor surface (**Fig. S8**). This relatively broad shell was chosen to encompass both immediate peritumoral tissue and the surrounding mechanically perturbed zones, ensuring inclusion of stress-transmitting interfaces while avoiding tumor-core artifacts. This width was specifically chosen to match the large physical scale of the meningiomas in our cohort (diameters 3.96–6.06 cm), ensuring the inclusion of the broader mechanically perturbed zones while maintaining statistical robustness for the inversion." (Lines 769-777).

"For pituitary adenomas, additional emphasis was placed on the biomechanical impact on the optic chiasm. A conservative 6 mm (2-pixel) peritumoral shell was delineated around the superior tumor margin in the coronal plane to ensure coverage of the optic chiasm region. The reduced shell thickness is specifically tailored to the compact anatomy of the optic chiasm, minimizing partial volume effects from non-visual structures." (Lines 782-787).

"In the ICP measurement cohort, we defined a 3-voxel (9 mm) inward shell from the cortical surface to capture the stress distribution across the brain's outer layer. This depth was optimized to focus on the brain-CSF interface, which can be analysed using the proposed model." (Lines 799-802).

Regarding the theoretical boundaries of our inversion. In wave-operator-based MRE, a quasi-local homogeneity assumption is a standard prerequisite. In our framework, this assumption is expected to hold within a local spatial window comparable to the shear wavelength, where material property gradients are small relative to the wave numbers. We have added the following clarifying statement to the Discussion section:

"The achievable spatial resolution of the proposed stress-mapping framework is primarily determined by the spatial resolution of the underlying MRE acquisition, therefore, extremely localized stress concentrations at sub-voxel scales cannot be fully resolved under current MRE resolution limits. Furthermore, our quasi-local homogeneity assumption is predicated on the local shear wavelength; while a standard simplification in elastography, its capability to resolve clinically relevant gradients is supported by our numerical simulations (**Fig. 3, Fig. 4**) and in vivo results (**Fig. 5**).

Although the sliding window approach optimizes the balance between local sensitivity and inversion stability, the current resolution may still pose challenges for mapping stress in fine-scale anatomical structures." (Lines 581-590).

Specific Comments

1. In the second paragraph of the introduction, it seems like it would make more sense to start with the idea that there are both invasive and noninvasive categories and then highlight the fact that the noninvasive approaches are limited.

Response: We sincerely appreciate the reviewer's insightful suggestion regarding the logical flow of the second paragraph in the introduction. As recommended, we have revised the second paragraph of the Introduction as follows:

"Existing approaches fall into two categories: invasive and noninvasive. Invasive techniques, such as microdroplet deformation[28], or biopsy-based mechanical testing[29–31], can directly probe stress but are inherently destructive, unsuitable for delicate tissues like the brain, and incapable of longitudinal monitoring. However, the noninvasive quantification of stress in soft tissues, despite its profound clinical and research significance, remains a challenge. Noninvasive elastography methods detect acoustoelastic effects of stress-induced stiffness variations[19–21], but typically infer stress indirectly through wave speed changes, facing key technical limitations." (Lines 73-80).

2. There appears to be a discrepancy in labeling the patients in the text vs. Figure 5. The authors referred to the tumor of patient #1 as the frontal lobe lesion, looks like parietal lobe. Similarly, the tumor of patient #2 in Figure 5b is stated as the parietal lobe tumor, but it is a lesion in the frontal lobe. Perhaps the images are swapped?

Response: We apologize for the error in labeling. The reviewer is correct that the lesion described as Patient #1 (Fig. 5a) is located in the parietal lobe, and the one described as Patient #2 (Fig. 5b) is located in the frontal lobe. We confirm that the images themselves (Fig. 5a and 5b) are not swapped, but our accompanying text description mistakenly interchanged the anatomical labels for these two patients. We have now corrected this error throughout the manuscript to accurately reflect the true location of the lesions shown in Figure 5a and 5b. And we have corrected the paragraph as follows (changes are marked in blue in the manuscript):

"The parietal lobe lesion (#1, 58.3 cm^3, Ø 4.81 cm, Fig. 5a) exhibited a modest

inferior bias, with apex stress of 0.69 kPa rising to 1.02 kPa at the base. This pattern reflects expansion against the falx cerebri and downward compression of the adjacent cortex, in line with mechanical constraints imposed by the interhemispheric fissure.

In the frontal lobe tumor (#2, 32.4 cm³, Ø 3.96 cm, Fig. 5b), we observed pronounced superior polarization, with stress peaking at 1.83 kPa at the apex and falling to 0.82 kPa inferiorly. Upward growth beneath the convex parietal vault, where firm dural attachments and calvarial curvature limit downward bulging, likely concentrates compressive load at the upper boundary." (Lines 316-324).

3. In the caption of Figure 5, the authors describe "mean stress in the top σtop and bottom σbottom hemispheres." The top and bottom hemisphere definitions are confusing. The authors should either come up with new terminology, or mention the definition "the horizontal plane through the tumor centroid; σtop and σbottom are the average stresses above and below this plane" earlier in the caption. Same in the manuscript, it would be clearer if the authors could explain this numerical value before the first time mentioning it.

Response: Thank you for pointing this out. We have revised the caption of Figure 5:

"**Fig. 1: In vivo measurement of normal stress induced by meningiomas**. Panels **a-d** correspond to four tumors situated in different anatomical regions (e.g., parietal, frontal, occipital, and temporal regions). In each panel, subpanels **i–iii** show the x, y and z components of the MRE shear wave displacement field mapped onto the 3D tumor–brain interface, subpanel **iv** depicts the resulting stress distribution on the same surface, subpanel **v** presents a sagittal T1-weighted slice overlaid with the 1.5 cm peritumoral stress map, and subpanel **vi** plots mean stress in the top σ_{top} and bottom σ_{bottom} hemispheres. Stress was sampled within a 1.5 cm radial margin around each tumor. σ_{top} and σ_{bottom} are the average stresses measured in the superior and inferior regions, respectively, defined by the horizontal plane passing through the tumor centroid." (Lines 350-359).

Furthermore, we have added the definition to the main text at the point where the parameters are first introduced to ensure clarity before presenting the results in Figure 5. This insertion is placed after the general description of the stress inversion process:

"To evaluate the feasibility and robustness of our wave-based stress inversion framework under physiologically realistic conditions, we retrospectively analyzed MRE datasets from four patients with intracranial meningioma (Fig. 2). Shear wave displacement fields were acquired on a 3 T system, and tumor boundaries were segmented from co-registered T1-weighted images. Assuming that stress propagates along the local surface normal, we sampled wave information from a 1.5 cm

peritumoral shell (5 pixels) to constrain the inversion. This produced high resolution, 3D maps of stress at the tumor–brain interface, revealing spatially heterogeneous mechanical loads in vivo (Fig. 5; Fig. S4). The reconstructed stress fields exhibited strong anatomical correspondence with calvarial constraints, providing biomechanical plausibility for the inferred spatial patterns. For quantitative assessment, we define the "top" and "bottom" hemispheres by the horizontal plane passing through the tumor centroid, where σ_{top} and σ_{bottom} are the average stresses measured in the superior and inferior regions relative to this plane, respectively." (Lines 296-309).

4. In the caption of Figure 5, the authors describe "Panels (a)-(d) correspond to four tumors situated at progressively lower levels within the cranial vault," while Figure 5 (a) - (d) are not necessarily progressively lower as described. It would be better to describe panels (a) - (d) as tumors in different lobes or regions to make it more aligned with the manuscript.

Response: Thank you for pointing this out. We apologize for the confusion and have revised accordingly. In the main manuscript text, we have revised the general description regarding the anatomical positioning:

"Situated in various anatomical regions of the cranial vault (e.g., parietal, frontal, occipital, and temporal regions), the tumor volumes ranged from 32.4 to 116.5 cm³ (diameters 3.96–6.06 cm), with reconstructed stress magnitudes lay predominantly in the kPa regime[32]." (Lines 310-313).

In the caption of Figure 5, we have replaced the imprecise positional description with one that details the specific anatomical lobes/regions:

"**Fig. 2: In vivo measurement of normal stress induced by meningiomas**. Panels **a-d** correspond to four tumors situated in different anatomical regions (e.g., parietal, frontal, occipital, and temporal regions)." (Lines 350-352).

5. In Figure 5 vi, provide the numeric values of each bar for clarity of interpretation, or even better, change to a type of different type of graph that better represents the spatial patterns you want to show.

Response: Thank you for pointing this out. To enhance the clarity and interpretability of the results, we have added the corresponding numerical values next to each bar in the histogram of Figure 5 (vi). We considered alternative visualization strategies to further emphasize spatial stress patterns; however, because the primary goal of panel

(vi) is to provide a concise quantitative comparison between superior and inferior hemispheric stress (σ_{top} vs. σ_{bottom}), we elected to retain the bar representation while augmenting it with explicit numerical annotations. This preserves consistency with the summary metrics discussed in the text and avoids overcomplicating the figure. The revised Figure 5 is shown as follows:

“

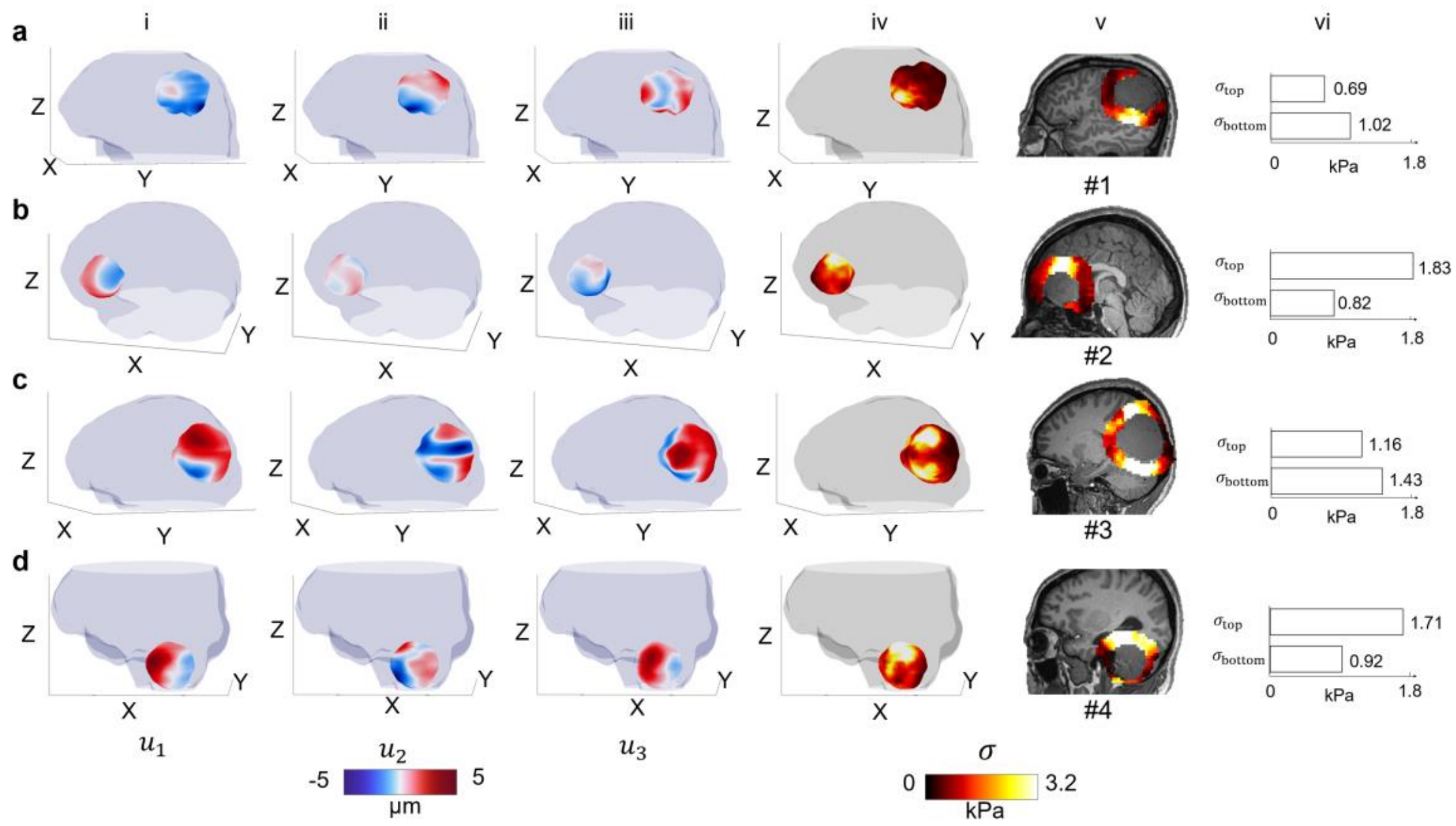

Fig. 3: In vivo measurement of normal stress induced by meningiomas. Panels **a-d** correspond to four tumors situated in different anatomical regions (e.g., parietal, frontal, occipital, and temporal regions). In each panel, subpanels **i–iii** show the x, y and z components of the MRE shear wave displacement field mapped onto the 3D tumor–brain interface, subpanel **iv** depicts the resulting stress distribution on the same surface, subpanel **v** presents a sagittal T1-weighted slice overlaid with the 1.5 cm peritumoral stress map, and subpanel **vi** plots mean stress in the top σ_{top} and bottom σ_{bottom} hemispheres. Stress was sampled within a 1.5 cm radial margin around each tumor. σ_{top} and σ_{bottom} are the average stresses measured in the superior and inferior regions, respectively, defined by the horizontal plane passing through the tumor centroid.” (Lines 349-359).

Code repository is empty.

Response: We sincerely apologize for this oversight. We have immediately rectified this issue. The core code used for data analysis and stress inversion, along with necessary associated data files, has been uploaded to the designated public code repository (https://github.com/aaronfeng369/MRE_stress) and is now accessible for verification and reproduction of the results.

Reviewer #4:

I co-reviewed this manuscript with one of the reviewers who provided the listed reports. This is part of the Nature Communications initiative to facilitate training in peer review and to provide appropriate recognition for Early Career Researchers who co-review manuscripts.

Response: We extend our sincere gratitude to both the reviewer and the co-reviewer for their valuable time and insightful feedback. We are especially thankful to Nature Communications for pioneering this initiative. The provided comments have significantly strengthened our manuscript.

Reference

1. Destrade, M. & Ogden, R. W. On the third- and fourth-order constants of incompressible isotropic elasticity. *J. Acoust. Soc. Am.* **128**, 3334–3343 (2010).
2. Nia, H. T. *et al.* Solid stress and elastic energy as measures of tumour mechanopathology. *Nat. Biomed. Eng.* **1**, 0004 (2016).
3. Jain, R. K., Martin, J. D. & Stylianopoulos, T. The Role of Mechanical Forces in Tumor Growth and Therapy. in *ANNUAL REVIEW OF BIOMEDICAL ENGINEERING, VOL 16* (ed. Yarmush, M.) vol. 16 321–346 (2014).
4. Budday, S., Ovaert, T. C., Holzapfel, G. A., Steinmann, P. & Kuhl, E. Fifty Shades of Brain: A Review on the Mechanical Testing and Modeling of Brain Tissue. *Arch. Comput. METHODS Eng.* **27**, 1187–1230 (2020).
5. Destrade, M., Gilchrist, M. D., Murphy, J. G., Rashid, B. & Saccomandi, G. Extreme softness of brain matter in simple shear. *Int. J. Non-Linear Mech.* **75**, 54–58 (2015).
6. Balbi, V., Trotta, A., Destrade, M. & Ní Annaidh, A. Poynting effect of brain matter in torsion. *Soft Matter* **15**, 5147–5153 (2019).
7. Tweten, D. J., Okamoto, R. J. & Bayly, P. V. Requirements for accurate estimation of anisotropic material parameters by magnetic resonance elastography: A computational study. *Magn. Reson. Med.* **78**, 2360–2372 (2017).
8. Smith, D. R. *et al.* Anisotropic mechanical properties in the healthy human brain estimated with multi-excitation transversely isotropic MR elastography. *Brain Multiphysics* **3**, (2022).
9. Feng, Y., Okamoto, R. J., Namani, R., Genin, G. M. & Bayly, P. V. Measurements of mechanical anisotropy in brain tissue and implications for transversely isotropic material models of white matter. *J. Mech. Behav. Biomed. Mater.* **23**, 117–132 (2013).
10. Tweten, D. J., Okamoto, R. J., Schmidt, J. L., Garbow, J. R. & Bayly, P. V. Estimation of material parameters from slow and fast shear waves in an incompressible, transversely isotropic material. *J. Biomech.* **48**, 4002–4009 (2015).
11. Ma, S. *et al.* Measurement of biomechanical properties of transversely isotropic biological tissue using traveling wave expansion. *Med. Image Anal.* **101**, 103457 (2025).
12. Burman Ingeberg, M., Van Houten, E. & Zwanenburg, J. J. M. Estimating the viscoelastic properties of the human brain at 7 T MRI using intrinsic MRE and nonlinear inversion. *Hum. Brain Mapp.* **44**, 6575–6591 (2023).
13. Jyoti, D. *et al.* Transversely-isotropic brain in vivo MR elastography with anisotropic damping. *J. Mech. Behav. Biomed. Mater.* **141**, (2023).

14. Jenkinson, M., Beckmann, C. F., Behrens, T. E. J., Woolrich, M. W. & Smith, S. M. FSL. *NeuroImage* **62**, 782–790 (2012).
15. Steiner, L. A. & Andrews, P. J. D. Monitoring the injured brain: ICP and CBF. *Br. J. Anaesth.* **97**, 26–38 (2006).
16. Sack, I. Magnetic resonance elastography from fundamental soft-tissue mechanics to diagnostic imaging. *Nat. Rev. Phys.* **5**, 25–42 (2022).
17. Pavuluri, K. *et al.* Differential effect of dementia etiology on cortical stiffness as assessed by MR elastography. *NeuroImage Clin.* **37**, 103328 (2023).
18. Svensson, S. F. *et al.* Robustness of MR Elastography in the Healthy Brain: Repeatability, Reliability, and Effect of Different Reconstruction Methods. *J. Magn. Reson. IMAGING* **53**, 1510–1521 (2021).
19. Smith, D. R. *et al.* Individual Muscle Force Estimation in the Human Forearm Using Multi-Muscle MR Elastography (MM-MRE). *IEEE Trans. Biomed. Eng.* **70**, 3206–3215 (2023).
20. Gennisson, J.-L. *et al.* Acoustoelasticity in soft solids: Assessment of the nonlinear shear modulus with the acoustic radiation force. *J. Acoust. Soc. Am.* **122**, 3211–3219 (2007).
21. Martin, J. A. *et al.* Gauging force by tapping tendons. *Nat. Commun.* **9**, 1592 (2018).
22. Zhang, Z. *et al.* Noninvasive measurement of local stress inside soft materials with programmed shear waves. *Sci. Adv.* **9**, eadd4082 (2023).
23. Parker, K. J., Ormachea, J., Zvietcovich, F. & Castaneda, B. Reverberant shear wave fields and estimation of tissue properties. *Phys. Med. Biol.* **62**, 1046–1061 (2017).
24. Zvietcovich, F., Pongchalee, P., Meemon, P., Rolland, J. P. & Parker, K. J. Reverberant 3D optical coherence elastography maps the elasticity of individual corneal layers. *Nat. Commun.* **10**, 4895 (2019).
25. Kabir, I. E. *et al.* Reverberant magnetic resonance elastographic imaging using a single mechanical driver. *Phys. Med. Biol.* **68**, 055015 (2023).
26. Pagé, G. *et al.* Comparison of ultrasound elastography, magnetic resonance elastography and finite element model to quantify nonlinear shear modulus. *Phys. Med. Biol.* **68**, 205003 (2023).
27. Crutison, J., Sun, M. & Royston, T. J. The combined importance of finite dimensions, anisotropy, and pre-stress in acoustoelastography. *J. Acoust. Soc. Am.* **151**, 2403–2413 (2022).
28. Bi, Y. *et al.* Mechanical models and measurement methods of solid stress in tumors. *Appl. Microbiol. Biotechnol.* **108**, 363 (2024).
29. Nia, H. T. *et al.* In vivo compression and imaging in mouse brain to measure the

effects of solid stress. *Nat. Protoc.* **15**, 2321–2340 (2020).

30. Nia, H. T. *et al.* Solid stress and elastic energy as measures of tumour mechanopathology. *Nat. Biomed. Eng.* **1**, 0004 (2016).
31. Nia, H. T. *et al.* Quantifying solid stress and elastic energy from excised or in situ tumors. *Nat. Protoc.* **13**, 1091–1105 (2018).
32. Feng, Y., Murphy, M. C. C., Hojo, E., Li, F. & Roberts, N. Magnetic Resonance Elastography in the Study of Neurodegenerative Diseases. *J. Magn. Reson. IMAGING* https://doi.org/10.1002/jmri.28747 (2023) doi:10.1002/jmri.28747.

Response to Reviewers

We sincerely thank the reviewers for their careful reading of our manuscript and for providing constructive critiques. We have addressed all questions and comments point-by-point below, indicating corresponding revisions in the manuscript. All changes in the revised manuscript have been marked in blue.

Reviewer #1:

I appreciate the authors' responses to my previous comments. While the manuscript has been improved by these revisions, several major limitations remain in the current version.

Response: We sincerely thank the reviewer for the positive assessment of our work. We appreciate the constructive critiques, which have helped us significantly improve the rigor and clarity of the manuscript. We have addressed each of the concerns accordingly as follows.

(1) The assumption of a uniaxial stress state appears both incorrect and unnecessary. First, the stress in brain tissue is inherently three-dimensional. Second, as demonstrated in Fig. S3c, the stress identified by the proposed method corresponds to the deviatoric stress component, regardless of the actual stress state. Given this, it is unnecessary to assume that the underlying stress is uniaxial.

To more clearly convey the concept of stress mapping, I suggest formulating the method under a general three-dimensional stress state and explicitly stating in the Methods section that the proposed approach derives the deviatoric component of the stress tensor.

A related issue concerns the stretch ratio derived from the proposed method. As indicated by Eq. (S8), a power of 1/3 is adopted, which implicitly depends on the assumed stress state. The identified stretch ratio is therefore not independent of this assumption. This point should be clarified and justified.

Response: We sincerely thank the Reviewer for this insightful suggestion. We fully agree that the stress state in brain tissue is inherently three-dimensional, and that, as demonstrated in Fig. S3c, our method specifically extracts the deviatoric stress component regardless of the actual stress state. Following the Reviewer's suggestion, we have revised the Methods section to clarify that the output of the inversion

corresponds to the deviatoric stress difference:

"Although the stress state in biological tissue is generally multi-axial, shear wave propagation in an incompressible medium is insensitive to the hydrostatic component and responds to the deviatoric part of the stress tensor (**Supplementary Note 5**). The proposed inversion recovers the deviatoric stress difference along the dominant loading direction under a uniaxial-dominant assumption, which provides both analytical tractability and a well-posed inverse problem." (Lines 629-634).

We also wish to clarify that, despite this general physical picture, the uniaxial-dominant assumption is still needed for the inversion framework. Under uniaxial loading of a Mooney–Rivlin material, the SH and SV modes possess closed-form polarizations and wave speeds governed by only three effective moduli (μ_A, μ_B, μ_C), which enables the construction of the operator-based inversion (Eqs. 3–4). Otherwise, the wave equations become a coupled 3D eigenvalue problem, whose polarization structure no longer admits a simple geometric decomposition, making it impossible to obtain an analytical solution. In addition, without this assumption, the inverse problem would be severely underdetermined: three measurable effective moduli versus five independent unknowns $(\lambda_1, \lambda_2, \lambda_3, C_{10}, C_{01})$. The uniaxial incompressibility constraint reduces the deformation to a single stretch ratio λ, making the system exactly determined. Finally, a general multiaxial inversion would additionally require knowledge of all three principal stress directions at every voxel, which cannot be obtained.

Therefore, with regard to the stretch ratio $\lambda = \left(\frac{\mu_A}{\mu_C}\right)^{\frac{1}{3}}$: this expression indeed derives from the uniaxial incompressibility relation and is not independent of that assumption. However, as demonstrated in Supplementary Note 5, when lateral stresses do not exceed one-third of the dominant stress, the estimation error remains below 10%

We believe that this uniaxial-dominant framework represents a principled balance between mathematical rigor and practical feasibility. In all clinical scenarios studied, peritumoral compression, optic chiasm loading, and cortical surface ICP, the dominant loading is approximately normal to the relevant anatomical boundary, providing direct physiological support for the assumption. We have revised the Discussion to make both the scope and the limitations of this assumption fully transparent:

"While our method demonstrates promising capabilities, it is subject to certain limitations. The full stress state in tissue is described by a second-order tensor comprising six independent components, making the recovery of all components from

a single shear-wave measurement impractical. Under the incompressibility assumption, shear-wave propagation is insensitive to hydrostatic pressure, leaving the full three-dimensional tensor theoretically underdetermined. To render the problem tractable, we adopted an isotropic hyperelastic model and focused on the dominant uniaxial stress component in a 3D formulation. Under a general triaxial state, the inverse problem is severely underdetermined and all three principal directions would need to be known at every voxel, information unavailable from a standard MRE acquisition. The uniaxial assumption reduces the system to a single stretch ratio, yields an determined inverse problem, and requires only one anatomically inferable direction. This simplification aligns with physiological observations where loading is predominantly normal to tissue boundaries." (Lines 566-579).

(2) The proposed method is inherently model-based, relying on an isotropic, incompressible M–N constitutive model. This represents an important limitation and should be explicitly acknowledged in the manuscript.

Response: We thank the Reviewer for this comment. While the Mooney-Rivlin model is widely adopted for brain tissue and has been validated in our simulation and phantom studies, it does constrain the generalizability of the framework to tissues or conditions where these assumptions hold. We have added explicit acknowledgments in the Results and Discussion sections of the revised manuscript:

"The brain is a soft, structurally complex tissue. While numerous models have been proposed to characterize its biomechanical behavior[1], isotropy and incompressibility remain standard simplifying assumptions[5]. Building on these assumptions, we propose a physics-informed traveling wave expansion (TWE) model. Derived from the hyperelastic Mooney–Rivlin constitutive model [2,3], the TWE framework describes the reverberant wavefield as a linear superposition of shear-vertical (SV) and shear-horizontal (SH) wave components[4,5] propagating along multiple directions (**Fig. 1c**)." (Lines 119-126).

"Future work should relax these simplifying assumptions, such as compressible, viscoelastic, or mechanically anisotropic tissues, and validate the method in large multi-center clinical studies, including dedicated viscoelastic phantom experiments, to test its robustness across pathologies, MRI scanners, and imaging protocols." (Lines 608-611).

(3) In the revised manuscript, the authors claim that the proposed method is effective

for heterogeneous media, yet no convincing evidence is provided. In the simulation involving an embedded sphere, the inversion is performed only in the region outside the inclusion, where the medium remains homogeneous. This does not demonstrate applicability within heterogeneous regions. Since tumors are intrinsically heterogeneous, clarification is needed regarding how the method performs under such realistic conditions.

Response: Thank you for pointing this out. We acknowledge that our original use of "heterogeneous" referred primarily to spatially varying stress distributions rather than to material-property heterogeneity, and we have revised the manuscript to distinguish these two concepts explicitly.

Regarding the clinical context, the stress inversion is not performed inside the tumor itself, but within the peritumoral shell, a region of brain parenchyma that, while mechanically perturbed, retains the constitutive character of normal brain tissue. The clinically relevant question is therefore whether the method can handle gradual spatial variations in tissue stiffness within this parenchymal region, rather than within the intrinsically heterogeneous tumor mass.

To clarify this issue, we performed new simulations in which the material properties vary linearly along the z-axis, producing a continuously graded shear modulus field. Results showed that the material heterogeneity did not significantly affect the stress estimation, as detailed in the new Supplementary Note 9:

"**Supplementary Note 9: Stress estimation in spatially heterogeneous media**

To evaluate the robustness of the proposed inversion framework in media with spatially varying material properties, we extended the embedded-sphere inflation simulation (Fig. 3) by introducing a linear gradient in the Mooney–Rivlin constants C_{10} and C_{01} along the z-axis. This produces a continuously graded shear modulus field $\mu(z) = 2[C_{10}(z) + C_{01}(z)]$ (with a fixed ratio of $C_{10}\colon C_{01} = 5\colon 7$) that varies smoothly across the computational domain, mimicking the possible mechanical heterogeneity encountered in peritumoral brain parenchyma.

Three modulus gradient magnitudes were tested, 3.4, 6.9, and 10 kPa/m, under both positive-gradient (μ increasing along +z) and negative-gradient (μ increasing along −z) configurations, yielding six independent test cases. In all cases, the heterogeneous expansion-induced stress field was first established quasi-statically, followed by 50 Hz shear wave excitation on the deformed configuration. Stress inversion was performed using the same operator-based framework with geometric surface normals as the assumed stress direction.

As shown in Fig. S7, the inversion accurately recovers the heterogeneous stress distribution across all tested gradient magnitudes and orientations. Despite local shear modulus variations spanning up to ~30% across the field of view, the mean estimation

errors remain between 10.76% and 14.43%, with no systematic degradation as the gradient steepens. Notably, the method correctly tracks the spatial stress profile under both positive and negative gradients, confirming that the quasi-local sliding-kernel inversion naturally adapts to gradual spatial variations in constitutive properties without requiring prior knowledge of the local modulus distribution. These results demonstrate that the proposed framework is applicable to mechanically heterogeneous media.

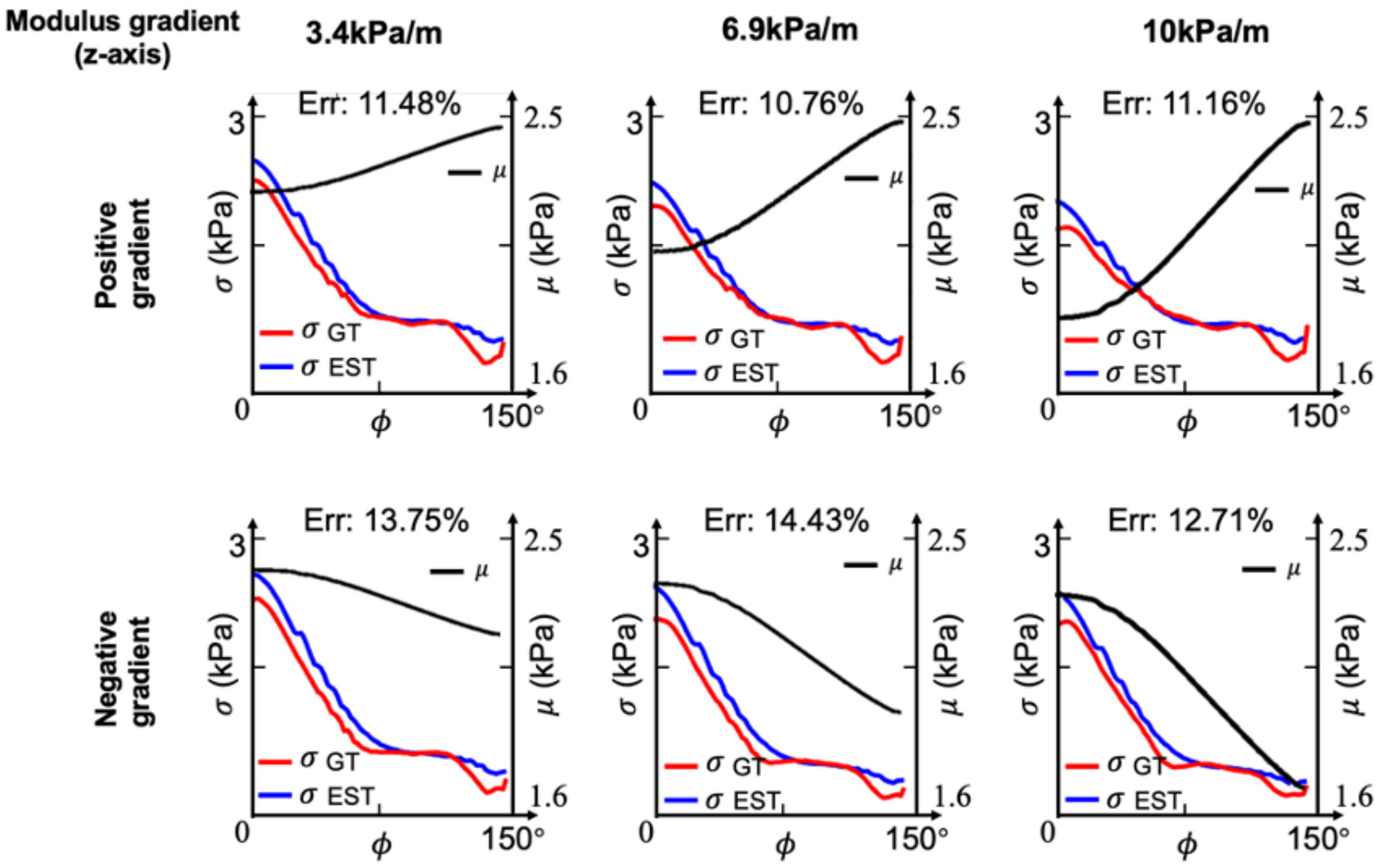

Fig. S7. Stress estimation accuracy in spatially heterogeneous media with linearly graded material properties. The simulation is based on the embedded-sphere inflation model (Fig. 3), with the shear modulus $\mu(z) = 2[C_{10}(z) + C_{01}(z)]$ varying linearly along the z-axis. Three modulus gradients (3.4, 6.9, and 10 kPa/m) are tested under positive-gradient (top row) and negative-gradient (bottom row) conditions. In each panel, σ_{GT} (red) and σ_{EST} (blue) denote the ground-truth and estimated stress as functions of elevation angle φ; the black curve shows the local shear modulus μ (right axis)." (in Supplementary Information).

We have also added clarifying statements in the Discussion.

"Furthermore, our quasi-local homogeneity assumption is predicated on the local shear wavelength; while a standard simplification in elastography, its capability to resolve clinically relevant gradients is supported by our numerical simulations (Fig. 3, Fig. 4) and in vivo results (Fig. 5). **Supplementary Note 9** demonstrates that the framework accurately recovers stress in media with spatially graded material properties (modulus gradients up to 10 kPa/m), with maximum estimation errors remaining below 15%." (Lines 586-592).

(4) I respectfully disagree with the authors' argument regarding the negligible effect of material damping. It is unclear how viscosity was incorporated into the simulations. Was an imaginary component introduced into the instantaneous shear modulus? If so, the simulation would not properly capture stress-dependent viscoelastic behavior.

To rigorously account for viscosity, a visco-hyperelastic constitutive model should be employed in the simulations. Intuitively, in the high-frequency regime, the phase velocity in soft tissues is often dominated by viscosity rather than stress. Consequently, the predicted speed anisotropy—and thus the proposed stress estimation approach—may depend strongly on wave frequency.

Given that no ground-truth stress data are available for brain tissue experiments, caution must be exercised before concluding that "material damping has negligible impact on stress estimation." This could represent a major limitation of the proposed method.

Have the authors considered performing validation experiments using viscoelastic phantoms to better support the method's applicability to real brain tissues?

Response: Thank you for pointing this out. We apologize for the overstatement of the previous claim of "negligible impact". In our previous damping treatment, an imaginary component was introduced into the complex shear modulus $G^* = G(1 + i\eta)$. Following your recommendation, we have used a visco-hyperelastic constitutive model for the validation simulation, as shown in the revised Supplementary Note 7 and Fig. S5:

"**Supplementary Note 7: The effect of visco-hyperelastic material behavior on stress estimation**

To evaluate the sensitivity of the proposed stress-inversion framework to viscoelastic material behavior, we performed systematic simulations using a visco-hyperelastic constitutive model. The Mooney–Rivlin hyperelastic model was coupled with a generalized Maxwell viscoelastic model (single branch, relaxation time τ =3 ms) in COMSOL Multiphysics. Three energy factors ($\alpha =$ 0.18, 0.33, 0.46) were selected to produce damping ratio $G'/G''=$0.1, 0.2, and 0.3 at 50 Hz, spanning the physiologically reported range for in vivo brain tissue at MRE frequencies.

In a prestressed visco-hyperelastic medium, the effective stiffness is direction-dependent; viscoelastic relaxation acting on these anisotropic stiffnesses may introduce additional directional variations in phase velocity beyond those caused by the acoustoelastic effect alone, potentially biasing the stress estimate. The visco-hyperelastic simulations performed here inherently capture this coupling, as the complex dynamic modulus self-consistently reflects both the prestress-induced anisotropy and the viscoelastic response.

Computation results demonstrate that, despite the potential for viscosity-induced phase-velocity bias, stress estimation accuracy degrades gradually and remains robust across the tested damping range (Fig. S5). For the homogeneous shell model, the relative estimation errors were 5.13%, 7.67%, and 17.06% for loss tangent values of 0.1, 0.2, and 0.3, respectively. In the heterogeneous embedded-sphere scenario, the corresponding errors were 9.99%, 15.63%, and 23.19%. In both scenarios, the spatial stress distribution is preserved and the dominant error is a systematic magnitude bias rather than a distortion of the stress pattern. These findings indicate that, within the physiological damping range at 50 Hz, the acoustoelastic effect remains the dominant source of directional phase-velocity anisotropy, and the additional bias introduced by viscoelastic dispersion does not substantially confound the clinical stress patterns reported in this study.

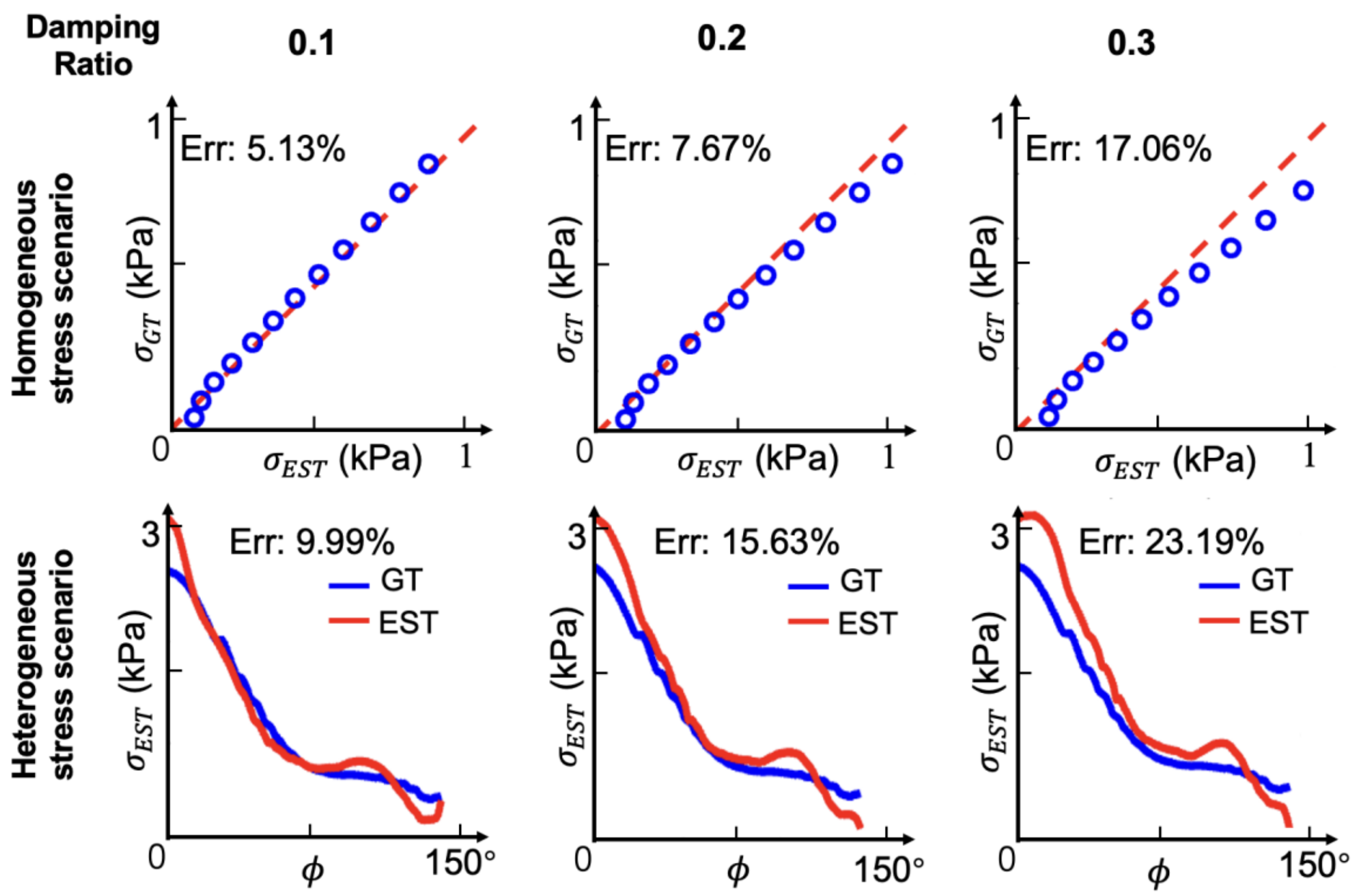

Fig.S5 Stress estimation under visco-hyperelastic material modeling. A generalized Maxwell viscoelastic model (single branch, τ = 3 ms) is coupled with the Mooney-Rivlin hyperelastic framework. Three loss tangent values (damping ratio G'/G''= 0.1, 0.2, 0.3) are tested. **Top row** (Fig. 2): Pressurized hemispherical shell simulation. Scatter plots compare ground-truth stress (y-axis) with estimated stress (x-axis). The red dashed line indicates the ideal 1:1 correspondence. **Bottom row** (Fig. 3): Heterogeneous embedded-sphere inflation simulation. Stress estimation error increases monotonically (9.99% to 23.19%), while the spatial stress pattern is well preserved. Together, these results confirm the robustness of the inversion framework to physiologically relevant levels of viscoelastic damping." (in Supplementary Information).

The results show that estimation errors increase monotonically with damping, confirming that viscoelastic effects do introduce a measurable bias. However, the spatial stress pattern is well preserved across all tested levels, and the dominant error is a systematic magnitude shift rather than a distortion of the stress distribution. We apologize for the overstatement of the previous claim of "negligible impact" and have revised the manuscript to provide a more balanced assessment:

"Supplementary Note 7 examines the effect of viscoelastic damping using a visco-hyperelastic constitutive model. Across the physiologically relevant damping range at 50 Hz, estimation errors increase monotonically but remain moderate, and the spatial stress pattern is well preserved, indicating that stress-induced acoustoelastic anisotropy remains the dominant contributor to directional phase-velocity variations at the operating frequency of this study." (Lines 599-605).

In this study, the gel phantom made of gelatin, glycerol and water is viscoelastic[6]. The viscoelastic properties of the gel phantom has been studied and modeled in many studies[7,8]. Since the focus of this study is to estimate the tissue stress, assumptions of models and stress state were adopted to achieve this in vivo estimation noninvasively. The focus of damping effect, its characterization and modeling will merit another separate study. Therefore, future studies of the damping effect have been added to the discussion section:

"Future work should relax these simplifying assumptions, such as compressible, viscoelastic, or mechanically anisotropic tissues, and validate the method in large multi-center clinical studies, including dedicated viscoelastic phantom experiments, to test its robustness across pathologies, MRI scanners, and imaging protocols." (Lines 608-611).

References

1. Budday, S., Ovaert, T. C., Holzapfel, G. A., Steinmann, P. & Kuhl, E. Fifty Shades of Brain: A Review on the Mechanical Testing and Modeling of Brain Tissue. *Arch. Comput. METHODS Eng.* **27**, 1187–1230 (2020).

2. Destrade, M., Gilchrist, M. D., Murphy, J. G., Rashid, B. & Saccomandi, G. Extreme softness of brain matter in simple shear. *Int. J. Non-Linear Mech.* **75**, 54–

58 (2015).

3. Balbi, V., Trotta, A., Destrade, M. & Ní Annaidh, A. Poynting effect of brain matter in torsion. *Soft Matter* **15**, 5147–5153 (2019).
4. Tweten, D. J., Okamoto, R. J. & Bayly, P. V. Requirements for accurate estimation of anisotropic material parameters by magnetic resonance elastography: A computational study. *Magn. Reson. Med.* **78**, 2360–2372 (2017).
5. Smith, D. R. *et al.* Anisotropic mechanical properties in the healthy human brain estimated with multi-excitation transversely isotropic MR elastography. *Brain Multiphysics* **3**, (2022).
6. Okamoto, R. J., Clayton, E. H. & Bayly, P. V. Viscoelastic properties of soft gels: comparison of magnetic resonance elastography and dynamic shear testing in the shear wave regime. *Phys. Med. Biol.* **56**, 6379–6400 (2011).
7. Feng, Y. *et al.* Viscoelastic Characterization of Soft Tissue-Mimicking Gelatin Phantoms using Indentation and Magnetic Resonance Elastography. *JOVE-J. Vis. Exp.* https://doi.org/10.3791/63770 (2022) doi:10.3791/63770.
8. Nguyen, M. M., Zhou, S., Robert, J., Shamdasani, V. & Xie, H. Development of Oil-in-Gelatin Phantoms for Viscoelasticity Measurement in Ultrasound Shear Wave Elastography. *Ultrasound Med Biol* **40**, 168–176 (2014).

Response to Reviewers

We sincerely thank the reviewers for their careful reading of our manuscript and for providing constructive critiques. We have addressed all questions and comments point-by-point below, indicating corresponding revisions in the manuscript. All changes in the revised manuscript have been marked in blue.

Reviewer #1:

I appreciate the authors' responses to my previous comments. The manuscript has been further improved through these revisions. However, my major concern regarding this work remains unchanged: although the proposed method is interesting, it may not yet be ready for clinical application because of the unrealistic assumptions adopted in the inversion framework. Consequently, the results should be interpreted and reported with substantial caution.

Response: We thank the reviewer for recognizing the improvements made in our previous revision. We also appreciate the continued critical scrutiny, which helps us further strengthen the manuscript. We have addressed the two specific concerns below.

(1) The assumption of a uniaxial stress state is overly restrictive and not physiologically realistic. The authors argue that the physiological stress state is primarily normal to the boundary and therefore dominated by uniaxial stress. However, this reasoning is not convincing. A normal stress acting around a cavity or tumor boundary will inevitably generate stress components in other directions with comparable magnitudes. Therefore, the actual stress state is expected to be multiaxial rather than uniaxial.

Response: We agree with the reviewer that the true in vivo stress state around tumors or cavities is inherently multiaxial. However, reconstructing a full 3D stress tensor from a single MRE acquisition is underdetermined. Our method was specifically designed to filter out hydrostatic pressure bias and extract the dominant deviatoric stress difference.

To demonstrate the validity of this approach, we performed extensive multiaxial loading simulations in Supplementary Note 5. The results clearly show that even when the lateral stress reaches 1/3 of the primary stress, the estimation error for the primary deviatoric stress remains below 5%. This confirms that our inversion remains highly accurate and robust for the dominant stress component, even under moderate multiaxial interference.

Nevertheless, we concede that this does not capture the complete 3D tensor complexity. To align with your recommendation, we have explicitly noted this simplification in the revised Discussion, advising readers to interpret the results with caution in highly multiaxial environments:

"Although these assumptions may not fully capture the complete tensor complexity in highly multiaxial environments, numerical simulations (Supplementary Note 5) demonstrate that the framework robustly recovers the primary deviatoric stress with less than 5% error under moderate lateral loading (lateral ratio up to 1/3)." (Lines 486-489).

(2) The authors acknowledge the influence of viscoelasticity and attempt to demonstrate through simulations that the associated errors are negligible. In my opinion, this validation is misleading. The simulations employ a stressrelaxation timescale on the order of milliseconds, corresponding to kHz frequencies, which are much higher than the 50 Hz shear waves used in MRE. This choice substantially suppresses viscoelastic effects and therefore minimizes the estimated errors. In practice, however, the relaxation timescale of brain tissues can be on the order of seconds, which could produce significantly larger viscoelastic effects at 50 Hz.

Given the multiple assumptions adopted in this work, including the uniaxial stress state, constitutive modeling choices, and the treatment of viscoelasticity, substantial caution is required when interpreting the reported brain tissue stress measurements. Without independent experimental validation or direct characterization of stress in real tissues, I do not believe the reported stress values can yet be considered reliable.

Response: We thank the reviewer for raising this important point. We agree that brain tissue exhibits long-term relaxation timescales on the order of seconds. However, for a time-harmonic wavefield at a fixed operating frequency (50 Hz), the key parameter governing viscous effects is the damping ratio (loss tangent, G''/G'). In our generalized Maxwell model, we specifically selected τ=3 ms and tuned the energy factors to produce damping ratios of 0.1, 0.2, and 0.3 at 50 Hz. These values directly correspond to the physiologically reported range for in vivo brain tissue at MRE frequencies[1,2]. If we were to use a 1-second relaxation time in a single-branch model at 50 Hz, it would yield an unphysiologically high loss tangent, failing to represent realistic in vivo damping.

As detailed in **Supplementary Note 7**, even at the upper physiological limit (damping ratio = 0.3), the stress estimation error remains bounded (17%–23%), taking the form of a systematic magnitude bias rather than a distortion of the spatial stress pattern.

While our parameters correctly capture the 50 Hz damping, we acknowledge the reviewer's broader point: a single-branch model may not fully represent the multi-scale, broadband viscoelasticity of brain tissue. However, our method provides bulk quantitative estimates of stress. While the overall magnitude and trends are consistent with expected values, the estimates may be refined with more complex models or additional constraints. However, the general magnitude is expected to remain stable. Therefore, we have revised the Discussion to emphasize this limitation and caution against overinterpreting the absolute numerical values:

"Across the physiologically relevant damping ratio range at 50 Hz (0.1~0.3), evaluations in Supplementary Note 7 show that estimation errors increase monotonically but remain bounded (up to 23%), and the spatial pattern is well preserved. The proposed method provides bulk quantitative estimates of stress. While the overall magnitude and trends are consistent with expected values, the estimates may be refined with more complex models or additional constraints. However, the general magnitude is expected to remain stable." (Lines 506-512).

Reviewer #1 (Remarks on code availability):

I did not assess the code associated with this manuscript.

Response: The code and data are publicly available at the GitHub repository (DOI provided in Code Availability statement) for future independent assessment.

Reference

1. Hiscox, L. V. et al. Magnetic resonance elastography (MRE) of the human brain: technique, findings and clinical applications. Phys. Med. Biol. 61, R401–R437 (2016).

2. Manduca, A. et al. MR elastography: Principles, guidelines, and terminology. Magn. Reson. Med. 85, 2377–2390 (2021).
